# RNF220 safeguards meiotic synapsis and male fertility by antagonizing SYCE1 ubiquitination

**DOI:** 10.1038/s41419-026-09128-8

**Published:** 2026-07-24

**Authors:** Jingmei Cha, Huijuan Lin, Yingqian Xia, Jianlin Xu, Pengcheng Ma, Shuhua Zhao, Bingyu Mao

**Affiliations:** 1 State Key Laboratory of Genetic Evolution and Animal Models, Kunming Institute of Zoology, Chinese Academy of Sciences, Kunming, 650201 China; 2 Kunming College of Life Science, University of Chinese Academy of Sciences, Kunming, 650223 China; 3 National Research Facility for Phenotypic & Genetic Analysis of Model Animals (Primate Facility), Kunming Institute of Zoology, Chinese Academy of Sciences, Kunming, 650201 China; 4 Department of reproduction and genetics, the First Affiliated Hospital of Kunming Medical University, Kunming, 650032 China

**Keywords:** Spermatogenesis, Infertility

## Abstract

A homozygous mutation in *RNF220*, encoding a RING-type E3 ubiquitin ligase, is associated with male infertility featuring small-headed sperm, but the precise roles and mechanisms of *RNF220* in spermatogenesis remain elusive. Here, we explored the function of *RNF220* in spermatogenesis using a Stra8-Cre-mediated germ cell-specific conditional knockout mouse model. *RNF220* was highly expressed in mouse spermatocytes, and its germ cell-specific deletion caused male subfertility, accompanied by testicular atrophy, oligozoospermia, and abnormal sperm morphology. Spermatocyte nuclear spreading assay revealed severe defects in homologous chromosome synapsis and DNA damage repair, as well as elevated apoptosis in pachytene spermatocytes. Mechanistically, SYCE1, a core component of the synaptonemal complex central element essential for synapsis, was identified as a testicular target of RNF220. Notably, RNF220 stabilized SYCE1 by antagonizing its ubiquitination mediated by other ubiquitin ligases. *RNF220* deficiency led to reduced SYCE1 protein level and marked increase in its K48-linked polyubiquitination. Furthermore, RNF220-mediated SYCE1 stabilization was independent of its intrinsic ubiquitin ligase activity. Our findings define the essential role of *RNF220* in spermatogenesis and provide novel molecular insights into the pathogenesis of male infertility.

## Introduction

Spermatogenesis in mammals is a multi-stage, precisely regulated biological process [1]. Meiosis serves as a pivotal bridging step in this process, being not only an essential pathway for generating haploid gametes but also a critical quality control phase that ensures the accurate transmission and recombination of genetic information [2, 3]. During the extended prophase of meiosis I, spermatocytes undergo dramatic alterations in chromatin structure and conformation. From the leptotene, zygotene to pachytene stages, homologous chromosomes sequentially complete pairing, synapsis, and recombination [4, 5]. This series of key events is accompanied by global transcriptional reprogramming and the establishment of unique epigenetic modifications [6, 7]. Any dysregulation in this process can directly activate cell cycle checkpoints, leading to meiotic abnormalities and subsequently triggering germ cell apoptosis and male infertility [8].

Synapsis is the process by which homologous chromosomes are closely aligned laterally via the synaptonemal complex (SC), which provides the essential physical scaffold for DNA double-strand break repair and homologous recombination [9]. The SC is an evolutionarily conserved protein complex that appears as a zipper-like structure, primarily composed of lateral elements, a central element, and transverse filaments connecting them [10, 11]. In mammals, the lateral elements mainly include synaptonemal complex proteins 2 and 3 (SYCP2 and SYCP3), the transverse filaments are primarily constituted by synaptonemal complex protein 1 (SYCP1), while the central region contains proteins such as synaptonemal complex central elements 1, 2, and 3 (SYCE1, SYCE2, SYCE3), and testis-expressed protein 12 (TEX12) [12]. Among these, the central element protein SYCE1 is considered the “cornerstone” for initiating SC assembly. *Syce1* knockout mice exhibit complete synapsis failure, meiotic arrest at the pachytene stage, leading to male and female infertility [13, 14]. Transcriptionally, the expression of *Syce1* in the testis is regulated by *Sox30*, the loss of which disrupts meiotic synapsis and homologous recombination [15]. However, whether SYCE1 is regulated post-translationally and its potential roles during spermatogenesis remains poorly understood.

Ring finger protein 220 (RNF220), a RING-type E3 ubiquitin ligase, has been shown to be widely involved in neural development and human diseases through different target proteins [16]. RNF220 functions through mediating either K48-linked polyubiquitination and proteasomal degradation of its target or K63-linked ubiquitination or monoubiquitination [17–22] of the substrates to regulate their localization or activity. In Wnt signaling, RNF220 serves as a bridging molecule for the deubiquitinating enzyme USP7 to deubiquitinate and stabilize β-catenin [23]. In addition, a *RNF220* mutation has been shown to be associated with human infertility with small-headed sperm [24]. Although it has been suggested to work through regulating the stability of Sin3B during spermiogenesis, the exact roles and mechanisms of RNF220 during spermatogenesis remain elusive.

In this study, we generated a germ cell-specific conditional knockout mouse model to investigate the physiological function and potential molecular target of *RNF220* during spermatogenesis.

## Materials and methods

### Mice

*RNF220* floxed mice were generated as previously described [19]. The Stra8-GFPCre transgenic mouse line [25, 26] was generously provided by Professor Minghan Tong (Center for Excellence in Molecular Cell Science, Chinese Academy of Sciences).

All mice were on a C57BL/6 genetic background, and only male mice at 2 months of age were used unless otherwise indicated.

### Genotyping

All genotypes were identified by PCR using genomic DNA extracted from mouse tails, following the procedures described in previous studies [27]. The following primers were used: *RNF220 1*#: forward, 5′- CTGTTGATGAAGGTCCTGGTT-3′ and reverse, 5′- CAGGAAAATCAATAGACAACTT-3′; *RNF220 3*#: forward, 5′- CTGTTGATGAAGGTCCTGGTT-3′ and reverse, 5′-CTGATTTCCAGCAACCTAAA-3′; *Stra8-Cre*: forward, 5′- TTATGGATGCACAGAAACTCCAAG-3′ and reverse, 5′- TGAACTTGTGGCCGTTTACGT-3′.

### Cell culture and transfection

Human HEK293T cells were obtained from the Kunming Cell Bank, Chinese Academy of Sciences. The cell line was authenticated and tested negative for mycoplasma contamination.

Human HEK293T cells were cultured in DMEM (Vivacell, C3113-0500) supplemented with 10% fetal bovine serum (Gibco, A5669701) and 1% penicillin-streptomycin (Vivacell, C3420-0100). All siRNA and plasmid transfections were performed using Lipofectamine 3000 (Invitrogen, L3000015). siRNAs targeting *RNF220* were purchased from RiboBio: siG000055182A/B, and *siRNF220-3:* GCGACUUAAGAACGCCAUGA.

### Fertility test and sperm count

Fertility testing: 8-week-old RNF220^fl/-^, Stra8-GFPCre (RNF220 cKO) male mice or RNF220^fl/fl^ (WT) male mice were individually caged with 8-week-old RNF220^fl/fl^ (WT) female mice in a single cage (one male : one female) for a continuous 5-month mating period. The morning when a vaginal plug was detected was designated as embryonic day 0.5 (E0.5). The average litter size within the 5-month period was calculated (total pups / total litters).

Epididymal sperm count: 8-week-old male mice were used for sperm collection. Both caudae epididymides were dissected using surgical scissors, placed in 2 mL of PBS and gently minced to reduce somatic cell release. The tissue was incubated at 37 °C for 10 min to allow sufficient sperm release. The sperm‑containing supernatant was transferred to a 15 mL centrifuge tube, and the culture dish was rinsed with 2 mL PBS, which was then added to the same tube. The pooled suspension was centrifuged at 300 × g for 5 min to sediment somatic cells, and the sperm‑enriched supernatant was carefully collected. Sperm were then fixed by adding 2 mL of 4% paraformaldehyde (PFA) in PBS and incubated for 10 min at room temperature. Finally, 100 μL of the fixed sperm suspension was mixed with 900 μL of 1×PBS, and sperm numbers were counted using a hemocytometer under an optical microscope [28, 29]. During counting, sperm morphology and purity were assessed to ensure minimal contamination by somatic cells or tissue debris.

Biological replicates were obtained from individual mice, with n representing the number of mice. The exact sample size is provided in the figure legends.

### Quantitative real-time PCR

Total RNA was extracted from mouse tissues using TRNzol Universal Total RNA Extraction Reagent (Tiangen, DP424). Biological replicates were obtained from individual mice, with n representing the number of mice. The exact sample size is provided in the figure legends. One microgram of total RNA was reverse-transcribed into cDNA using the Thermo Scientific RevertAid First Strand cDNA Synthesis Kit (Thermo, K1622). Real-time quantitative PCR (RT-qPCR) was performed using a Bio-Rad CFX96 real-time PCR system with 2× TSINGKE® Master qPCR Mix (SYBR Green I) (Tsingke Biotechnology, TSE201).

For primer design, the *RNF220*-E2 primer (located in exon 2), which targets the floxed allele, was used to assess *RNF220* knockout efficiency. The *RNF220*-E15 primer (located in exon 15), common to all transcripts, was employed to evaluate total *RNF220* expression levels. All primers used are listed below:

mouse *Actin*: forward, 5′-GCCAACCGTGAAAAGATGAC-3′ and reverse, 5′-GAGGCATACAGGGACAGCAC-3′; mouse *RNF220*-E2: forward, 5′-GTCTCAGTAGACAAGGACGTTCACA-3′ and reverse, 5′-GGGGTGGAGGTGTAGTAAGGAAG-3′; mouse *RNF220*-E15: forward, 5′-GAACAGCGACATTGAGAAAA-3′ and reverse, 5′-GTCAGAGGCATCGAGTAAGA-3′; mouse *Sycp1*: forward, 5′-TGAGGGGAAGCTCACGGTT-3′ and reverse, 5′-CGAACAGTGTGAAGGGCTTTTG-3′; mouse *Sycp2*: forward, 5′-GACACTGAAACCGAATGTGGA-3′ and reverse, 5′-TGTGGGTCTTGGTTGTCCTTT-3′; mouse *Sycp3*: forward, 5′-AGCCAGTAACCAGAAAATTGAGC-3′ and reverse, 5′-CCACTGCTGCAACACATTCATA-3′; mouse *Sox30*: forward, 5′-ACGGTGGGGAAGAGTACTTG-3′ and reverse, 5′-TTGACTTGCTGGACACCAGA-3′; mouse *Tex11*: forward, 5′- GGATCAAGGACTACTGATTGCAC -3′ and reverse, 5′- CACTTTGTGGACACCTGCTCA -3′; mouse *Casp3*: forward, 5′-GTCATCTCGCTCTGGTACGG-3′ and reverse, 5′-CACACACACAAAGCTGCTCC-3′.

Relative expression was calculated by the 2^−ΔΔCt method using Actin as the internal control.

### Histology: Hematoxylin and eosin (H&E) staining and periodic acid-Schiff (PAS) staining

Mouse testes and epididymides were collected and fixed in Bouin’s solution overnight at 4 °C. The fixed tissues were dehydrated, embedded in paraffin, and sectioned. The sections were deparaffinized and rehydrated for subsequent staining. Hematoxylin and eosin (H&E) staining was performed following the manufacturer’s instructions (Servicebio, G1005). Periodic acid–Schiff (PAS) staining was carried out using a PAS staining kit (Servicebio, G1008).

After PAS staining, images were acquired and the outer diameter of seminiferous tubules was measured using an Olympus digital slide microscope (VS120). We grouped the 12 stages of the spermatogenic cycle into four phases for analysis.

Data were obtained from three mice per genotype, the total number of seminiferous tubules analyzed is indicated on the statistical plots.

### Immunofluorescence staining

Frozen section preparation and immunofluorescence staining were performed as previously described [19]. Testicular frozen sections were cut at a thickness of 10 μm. Sections were blocked with 5% bovine serum albumin (BSA) in PBS containing 0.1% Tween-20 for 1 h at room temperature. Sections were incubated with primary antibodies diluted in 2% BSA at 4 °C overnight, followed by incubation with corresponding fluorescent secondary antibodies, DAPI, and Rhodamine-conjugated Peanut Agglutinin (PNA) (1:1000; Ruitaibio, RT6328) at room temperature for 2 h. PNA was used to label acrosomes for staging of the seminiferous epithelial cycle. Based on the developmental characteristics of spermatocytes and spermatids, the seminiferous epithelial cycle was classified into 12 stages (I–XII) [30]. Images were acquired using a spinning disk super-resolution confocal microscope (Olympus Ixplore SpinSR10).

For quantification, ImageJ software was used to count positive signals in cross-sections of seminiferous tubules. Positive signals were defined as clear green fluorescent signals distinct from background. We grouped the 12 stages of the spermatogenic cycle into four to five phases for analysis. Data were obtained from three mice per genotype, the total number of seminiferous tubules analyzed is indicated on the statistical plots.

Antibodies used in this study are listed in Supplementary Table S1.

### TUNEL staining

TUNEL staining was performed using the Promega TUNEL Apoptosis Detection Kit (Promega, G3250) strictly according to the manufacturer’s instructions. All testicular sections were deparaffinized, rehydrated, and permeabilized with 20 μg/ml Proteinase K.

Positive and negative control sections were prepared from wild-type (WT) mouse testes. For the positive control, sections were treated with DNase I (10 U/ml) in DNase I buffer at room temperature for 10 min prior to TdT incubation. For the negative control, sections were incubated in TdT reaction mixture lacking rTdT enzyme.

For experimental samples, sections were directly subjected to TUNEL labeling without additional treatment. After equilibration, sections were incubated with TdT reaction mixture at 37 °C for 60 min in the dark.

After termination of the reaction, the slides were washed with 2×SSC. Nuclei were counterstained with DAPI, and acrosomes were labeled with Rhodamine-conjugated Peanut Agglutinin (1:1000; Ruitaibio: RT6328) to assist in staging of the seminiferous epithelial cycle and identification of apoptotic cell types [30]. Finally, images were acquired using a spinning disk super-resolution confocal microscope (Olympus Ixplore SpinSR10).

For quantification, ImageJ software was used to count TUNEL-positive signals in cross-sections of seminiferous tubules. TUNEL-positive cells were defined as those showing clear green nuclear fluorescence distinct from background signals. Data were obtained from three mice per genotype. The total number of seminiferous tubules analyzed is provided in the figure legend.

### Spermatocyte spreading and immunofluorescence staining

Testes from 2-month-old mice were collected, and seminiferous tubules were washed in PBS and incubated in hypotonic solution for 30 min. The tubules were then transferred to a clean glass slide, minced thoroughly in 0.1 M sucrose solution (pH 8.2), and pipetted repeatedly to prepare a single-cell suspension. The cell suspension was dropped onto slides pre-treated with fixative containing 1% PFA and 0.15% Triton X-100 (pH 9.2), and dried in a humidified chamber for 4–6 h. Subsequently, the slides were washed twice with 0.4% Photo-Flo 200 (Kodak, 1464570), air-dried at room temperature, and stored at −80 °C until use.

Prior to immunofluorescence staining, slides were equilibrated to room temperature, washed twice with PBS for 5 min each with gentle agitation, and blocked with 10% donkey serum in a humidified chamber for 2 h. Subsequent staining was performed as described in the immunofluorescence staining method. Images were acquired using a spinning disk super-resolution confocal microscope (Olympus Ixplore SpinSR10).

For quantification, ImageJ software was used to determine the proportion of normal and abnormal pachytene spermatocytes. Abnormal pachytene spermatocytes were defined as those showing punctate, non-linear SYCP1 signals or diffuse γH2AX distribution on autosomes. Data were obtained from three mice per genotype. Individual cell counts from each animal are listed in the figure legend.

Antibodies used in this study are listed in Supplementary Table S1.

### WB, co-IP, and ubiquitination assays

For sperm proteins: Sperm protein extracts were prepared and subjected to Western blot analysis according to previously described methods [28, 29]. Briefly, mouse cauda epididymides were minced and incubated in PBS at 37 °C for 10 min to facilitate sperm release. Sperm concentration was determined using a hemocytometer, and sperm pellets were collected by centrifugation at 800 × g for 5 min at room temperature. Pellets were resuspended in 100 μL SDS-EDTA solution (1% SDS, 24 mM EDTA, 75 mM NaCl, pH 6.0), homogenized, and centrifuged at 5000 × g for 30 min at room temperature. Supernatants were collected as sperm protein samples.

For total tissue samples: For Western blot analysis, proteins were extracted using SDS lysis buffer (0.5 M Tris, 30% SDS, 5% 2-mercaptoethanol, 10% glycerol, pH 6.8) at 500 μL per 100 mg tissue.

For immunoprecipitation (IP) and ubiquitination (UB) assays, proteins were extracted using RIPA lysis buffer (Beyotime, P0013D).Western blot, in vitro and in vivo ubiquitination assays, and co-immunoprecipitation (co-IP) analyses were performed as previously described [19, 22, 23].

Band intensities were quantified using ImageJ. For each sample, the intensity of each target protein band was first normalized to that of the internal control. The resulting values were then further normalized to the wild-type (WT) group, setting the WT average as 1.0; statistical analyses were performed using data from three mice per group (relevant quantification results are presented in the corresponding figure panels).

Antibodies used in this study are listed in Supplementary Table S1.

### Flow cytometry sorting

Distinct spermatogenic cell populations were isolated from mouse testes by flow cytometry as previously described [25, 26, 31]. Initially, a single-cell suspension was prepared through sequential digestion of testicular tissues with Collagenase I (Sigma, 2674) and trypsin. Cells were stained with Hoechst 33342 (Sangon Biotech, E607302) for DNA content analysis, and propidium iodide (PI) (Sigma, P4170) was added prior to sorting to exclude dead cells.

Using a flow cytometer, cells were sorted based on differences in DNA content (Hoechst signal) and cell size (forward scatter, FSC): the diploid population was enriched for spermatogonia; the tetraploid population with high Hoechst signal was further subdivided by cell size into leptotene/zygotene, pachytene, and diplotene spermatocytes, while the haploid population with low Hoechst signal corresponded to spermatids.

The sorted cells were used for total RNA extraction and subsequent RT-qPCR analysis. Cell purity and identity were validated by nuclear spreading combined with immunofluorescence staining using specific antibodies such as SYCP3 and PNA. The purity of each sorted population typically exceeded 70%.

### In situ hybridization

For probe preparation: To detect the deletion of exon 2 in *RNF220* knockout mice, an *RNF220* probe specifically targeting exon 2 was designed. The probe was cloned into the pGEM-T vector and transcribed using an SP6 in vitro transcription kit [19]. Testicular tissues were sectioned at 15 μm thickness, and subsequent hybridization was performed according to a previously reported method [32, 33]. Sections were observed and images were acquired using an IX3-SVR microscope (Olympus).

### RNA-seq analysis

Testicular tissues from postnatal day 14 (P14) and 2-month-old mice were collected and immediately flash-frozen in liquid nitrogen. Sample processing and sequencing analysis for P14 samples were performed by Shanghai Majorbio Bio-pharm Technology Co., Ltd., while those for 2-month-old samples were conducted by Shanghai Applied Protein Technology Co., Ltd.

Differential expression analysis was performed using the *edgeR* software package based on gene read counts. Significantly differentially expressed genes were identified using the criteria of false discovery rate (FDR) < 0.05 and |log₂(fold change)| ≥ 1.

The raw RNA-seq data have been deposited in the NCBI Sequence Read Archive (SRA) under the BioProject accession number PRJNA1456370.

### Electron microscopy analysis

Sperm samples were collected following the procedure described in the aforementioned sperm counting experiment. The collected sperm suspension was centrifuged at 800 × g for 5 min, and the supernatant was discarded. The resulting pellet was fixed overnight at 4 °C with 2.5% glutaraldehyde. Samples were then post-fixed with 1% osmium tetroxide, dehydrated through an ethanol gradient, transitioned with acetone, and infiltrated and embedded in epoxy resin. Ultrathin sections (50 nm) were prepared and double-stained with uranyl acetate and lead citrate. Sperm flagellar ultrastructure was observed using a JEM-1400 Plus transmission electron microscope.

### Statistical analysis

No animals or samples were excluded from the analysis. No randomization was performed; mice were grouped based on genotype. The investigators were not blinded to group allocation during experiments and outcome assessment.

Biological replicates were obtained from individual mice. The definition of sample size (n) for each assay is specified in the relevant method sections and indicated in the corresponding figures or figure legends.

Wild-type and RNF220 cKO mice were age-matched within 7 days. Unless otherwise specified, data in the figures are expressed as mean ± SD. Statistical analyses were performed using GraphPad Prism 8 software. Comparisons between two groups were conducted using an unpaired two-tailed t-test. For average litter size, the Mann–Whitney U test was used due to non-normal distribution. Statistical significance was defined as *P < 0.05, **P < 0.01, ***P < 0.001.

## Results

### RNF220 is highly expressed in testis and peaks at the pachytene stage of meiosis

**Fig. 1:**
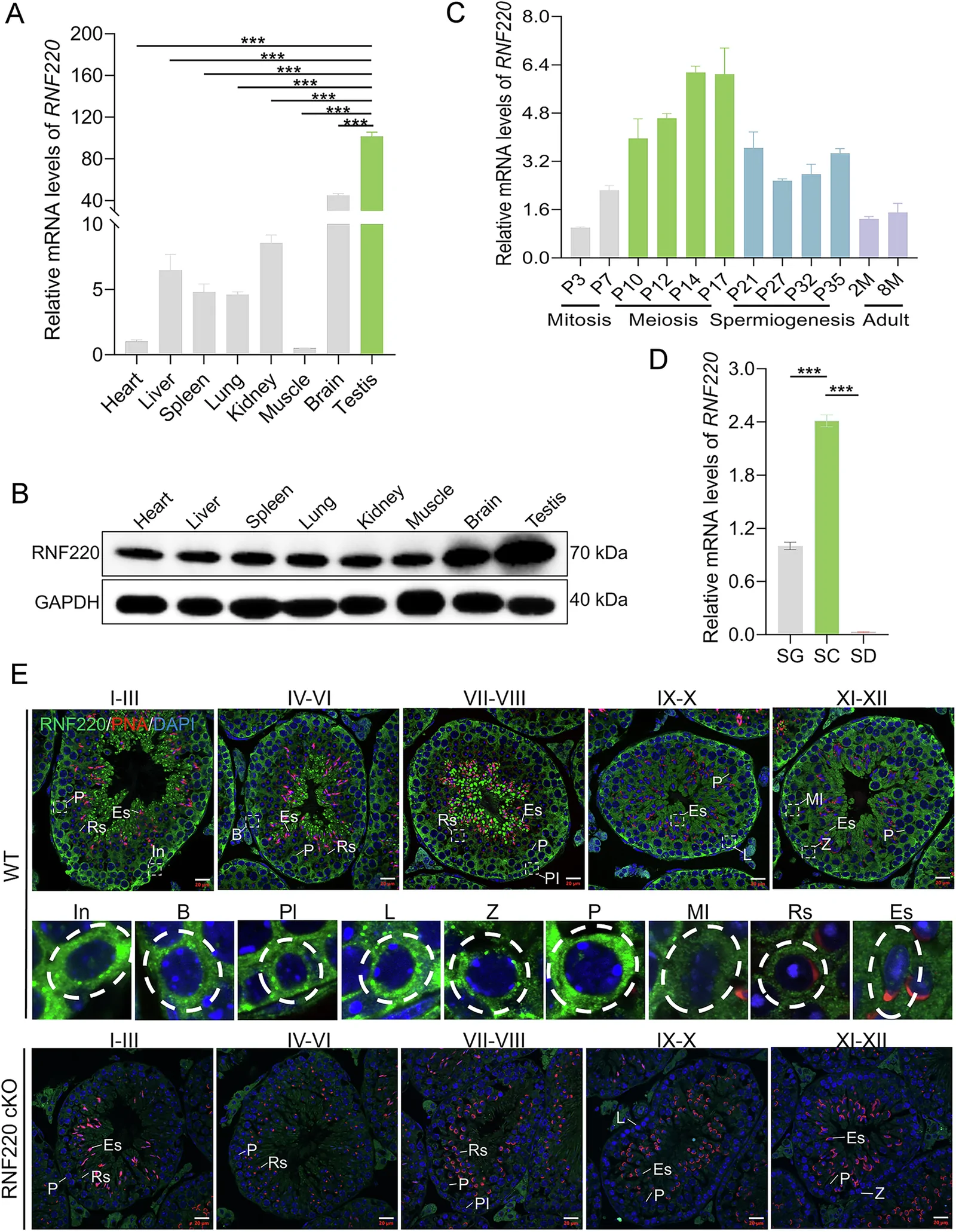
RNF220 expression in mouse testis. **A** Relative mRNA expression of *RNF220* in various tissues by RT‑qPCR, with heart set as 1 (*n* = 3 mice). ***P < 0.001 vs. testis. **B** Protein expression of RNF220 in various tissues by Western blot. GAPDH was used as the loading control. **C** Relative mRNA expression of *RNF220* in mouse testes at the indicated postnatal days by RT‑qPCR, with the P3 sample set as 1. Spermatogenic stages: mitotic phase (P3, P7), meiotic phase (P10–P17), spermiogenesis phase (P21–P32), and adulthood (2 M, 8 M) (*n* = 3 mice). **D** Relative mRNA expression of *RNF220* in flow-sorted testicular cell populations by RT‑qPCR, with SG set as 1. Cell types: diploid spermatogonia (SG), tetraploid spermatocytes (SC), and haploid spermatids (SD) (*n* = 3 mice). ***P < 0.001 vs. SC. **E** Representative immunofluorescence staining of RNF220 protein in mouse seminiferous tubules at distinct stages of the spermatogenic cycle. Upper panel: wild-type (WT) seminiferous tubules; middle panel: high-magnification views of WT cells; lower panel: RNF220 cKO seminiferous tubules. Triple staining: PNA (acrosome, red), RNF220 (green), DAPI (nuclei, blue). Intermediate spermatogonia (In), type B spermatogonia **B**, preleptotene spermatocytes (Pl), leptotene spermatocytes (L), zygotene spermatocytes (Z), pachytene spermatocytes (P), metaphase I spermatocytes (MI), round spermatids (Rs), and elongating spermatids (Es) are indicated. Scale bars: 20 μm. **Note:** The bright green signal in the lumen of wild-type (WT) tubules represents non-specific staining from sperm flagella, which is absent in RNF220 cKO tubules due to the significantly reduced sperm count in cKO mice.

We first analyzed the expression profile of RNF220 in various mouse tissues. Both RT-qPCR and Western Blot results confirmed that RNF220 is widely expressed in multiple tissues, with higher mRNA and protein levels in the testes and brain than in other tissues (Fig. 1A, B).

Given the high synchrony of the first postnatal spermatogenic wave in mice, we collected testicular samples at defined time points (P3, P7, P10, P12, P14, P17, P21, P27, P32, P35, 2 M, 8 M) to isolate stage-matched germ cell populations. RT-qPCR analysis revealed that the mRNA level of *RNF220* gradually increased from postnatal day 3 (P3) to P14, peaking at the pachytene stage of meiosis, and then remained stable. Following the onset of spermiogenesis (starting at P21), its expression level slightly decreased (Fig. 1C).

To validate these findings at the cellular level, we performed fluorescence-activated cell sorting (FACS) to isolate haploid (round and elongating spermatids), diploid (spermatogonia), and tetraploid (prophase I spermatocytes) germ cell populations from adult mouse testes (Supplementary Fig. S1A–C). RT-qPCR analysis demonstrated that *RNF220* expression was highest in the tetraploid cell populations, while it was barely detectable in spermatids (Fig. 1D). Mouse sperm morphogenesis is divided into 16 steps, corresponding to 12 stages of the seminiferous wave, each with distinct cellular associations [34, 35]. Peanut agglutinin (PNA) binds to the acrosome and can be used to identify the stages of sperm morphogenesis and classify the corresponding cell types during the seminiferous epithelial cycle. We performed co-immunofluorescence staining of RNF220 and PNA on frozen sections of adult mouse testes to determine the localization of RNF220 (Fig. 1E).

In the upper panel (stages I–VIII), RNF220 was highly expressed in spermatogonia and spermatocytes in the basal compartment of seminiferous tubules. In contrast, its expression was markedly lower in round spermatids (stages I–VIII) and elongating spermatids (stages IX–XII) in the adluminal compartment (Fig. 1E, upper panel).

High-magnification views of the different cell types further delineated the localization of RNF220 across the seminiferous cycle: RNF220 was detected in the cytoplasm of intermediate spermatogonia (In), type B spermatogonia (B), preleptotene/leptotene spermatocytes (Pl/L), zygotene spermatocytes (Z), and pachytene spermatocytes (P), with the strongest signal observed in pachytene spermatocytes. Only weak staining was present in metaphase I spermatocytes (MI), round spermatids (Rs), and elongating spermatids (Es) (Fig. 1E, middle panel). As a control, these signals were largely absent in RNF220-deficient (RNF220^f/-^, Stra8-Cre, see below) spermatogonia and spermatocytes in the lower panel, validating the specificity of the RNF220 antibody (Fig. 1E, lower panel).

### *RNF220* knockout impairs spermatogenesis in mice

**Fig. 2:**
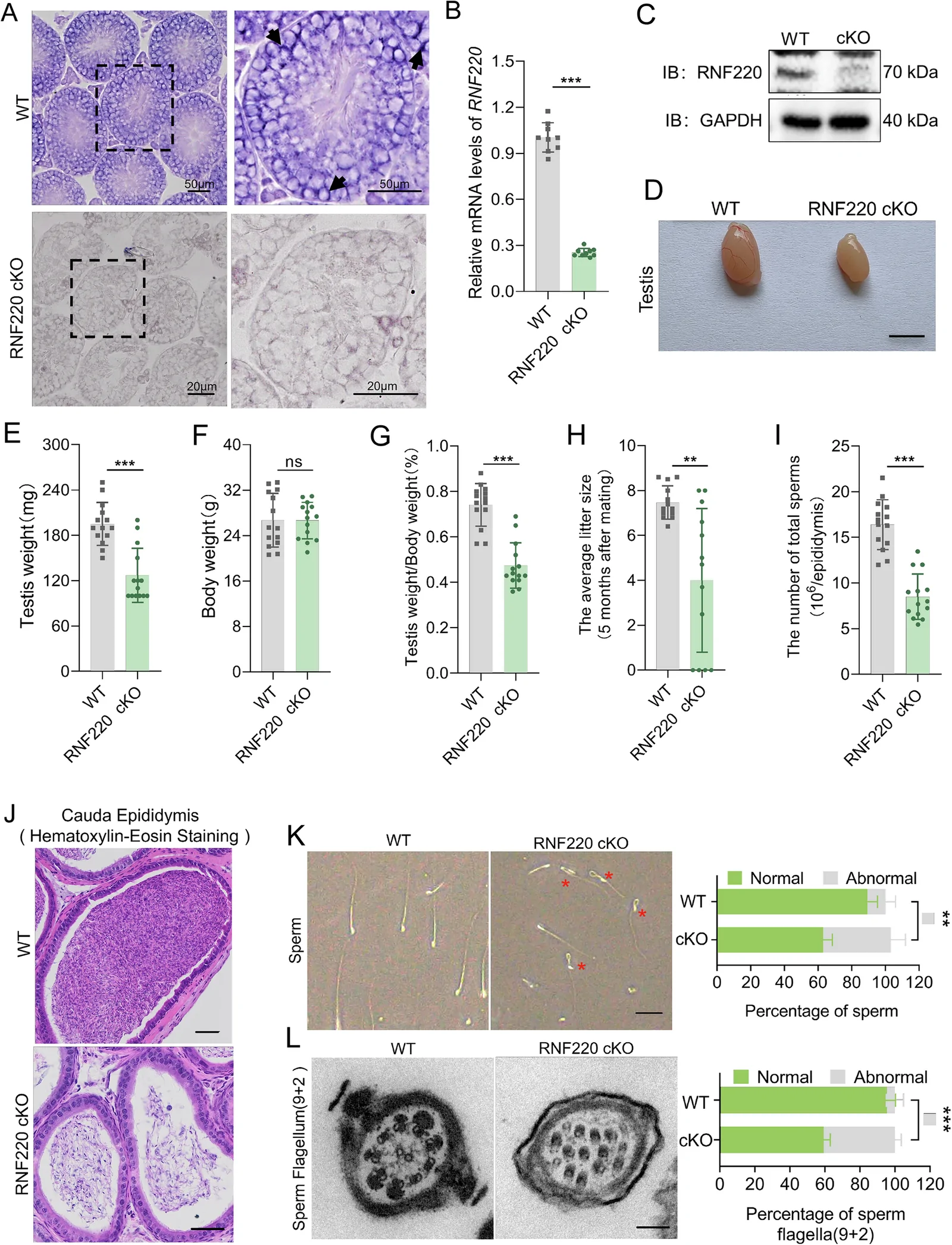
Conditional knockout of *RNF220* impairs male fertility and testicular development in mice. **A** Representative in situ hybridization images of *RNF220* mRNA (purple-blue signals) in testicular tissues from wild-type (WT) and RNF220 cKO mice. Positive signals are indicated by black arrows. Scale bars: 20 μm for WT, 50 μm for RNF220 cKO. **B** Relative mRNA expression of *RNF220* in testes from WT and RNF220 cKO mice by RT‑qPCR, with WT set as 1 (*n* = 9 mice). ***P < 0.001. **C** Protein expression of RNF220 in testes from WT and RNF220 cKO mice by Western blot. GAPDH was used as the loading control. **D** Representative macroscopic images of testes from WT and RNF220 cKO mice. Scale bar: 5 mm. **E** Testis weights of WT and RNF220 cKO mice (*n* = 14 mice). ***P < 0.001. **F** Body weights of WT and RNF220 cKO mice (*n* = 14 mice). ns not significant. **G** Testis-to-body weight ratio of WT and RNF220 cKO mice (*n* = 14 mice). ***P < 0.001. **H** Average litter size from matings between WT or RNF220 cKO male mice and WT female mice over a 5-month period (*n* = 13 males). **P < 0.01. **I** Sperm counts from the cauda epididymis of WT and RNF220 cKO mice (*n* = 14 mice). ***P < 0.001. **J** Representative H&E staining of the cauda epididymidis from WT and RNF220 cKO mice. Scale bars: 50 μm. **K** Left: Representative morphological images of cauda epididymal sperm from WT and RNF220 cKO mice. Red asterisks indicate sperm with head-tail folding malformation. Scale bar: 50 μm. Right: Quantitative statistics for the percentages of morphologically normal (green) and abnormal (gray) sperm. (*n* = 3 mice; counted sperm (*N*): WT1 = 259, cKO1 = 120; WT2 = 246, cKO2 = 114; WT3 = 236, cKO3 = 93). **P < 0.01 (WT vs. CKO, % abnormal sperm). **L** Left: Representative transmission electron microscopy (TEM) images of sperm flagellum cross-sections from WT and RNF220 cKO mice. Scale bar: 50 nm. Right: Quantification of the proportions of sperm flagella with intact (green) and aberrant (gray) 9 + 2 axonemal structure. (*n* = 3 mice; counted structures (*N*): WT1 = 20, cKO1 = 86; WT2 = 24, cKO2 = 63; WT3 = 30, cKO3 = 71). ***P < 0.001 (WT vs. cKO, % aberrant 9 + 2 axonemal structure).

To investigate the physiological function of *RNF220* in mouse spermatogenesis, we generated a germ cell-specific *RNF220* conditional knockout (cKO) mouse model using the Stra8-Cre (Supplementary Fig. S2A, B). As the floxed allele would be deleted in the sperm of the RNF220^f/wt^, Stra8-Cre mice, the conditional knockout mice were RNF220^f/-^, Stra8-Cre in genotype. Successful knockout of RNF220 *i*n mouse testis was confirmed by in situ hybridization, RT-qPCR, Western blot (Fig. 2A–C), and immunofluorescence staining (Fig. 1E).

Following effective *RNF220* knockout (Fig. 2A–C), the cKO mice exhibited markedly reduced testis size (Fig. 2D) and weight (Fig. 2E). However, body weight remained unchanged in the cKO mice (Fig. 2F), leading to a significant decrease in the testis-to-body weight ratio (Fig. 2G). As reported previously, some of the cKO mice showed progressively decreasing mobility to different extents due to RNF220 haploinsufficiency [36]. Nevertheless, they showed much reduced fertility with fewer pups per litter(Fig. 2H; Supplementary Fig. S2C). Sperm count analysis demonstrated a significant reduction in mature sperm number within the cauda epididymis of cKO mice (Fig. 2I). H&E staining of the cauda epididymidis revealed significantly reduced sperm density in RNF220 cKO mice (Fig. 2J). In humans, *RNF220* mutation has been reported to lead to small-headed sperm. We examined whether loss of RNF220 would affect the sperm morphology. Indeed, approximately 40% of spermatozoa from RNF220 cKO mice exhibited prominent head-tail folding malformations, significantly higher than WT controls (Fig. 2K). Meanwhile, transmission electron microscopy revealed that approximately 41% of RNF220 cKO sperm exhibited disorganized flagellar “9 + 2” axonemal microtubule architecture, a proportion significantly higher than WT controls (Fig. 2L). These findings collectively indicate that RNF220 plays an indispensable role in maintaining normal spermatogenesis and male fertility in mice.

### *RNF220* deficiency disrupts meiotic synapsis and DNA damage repair

**Fig. 3:**
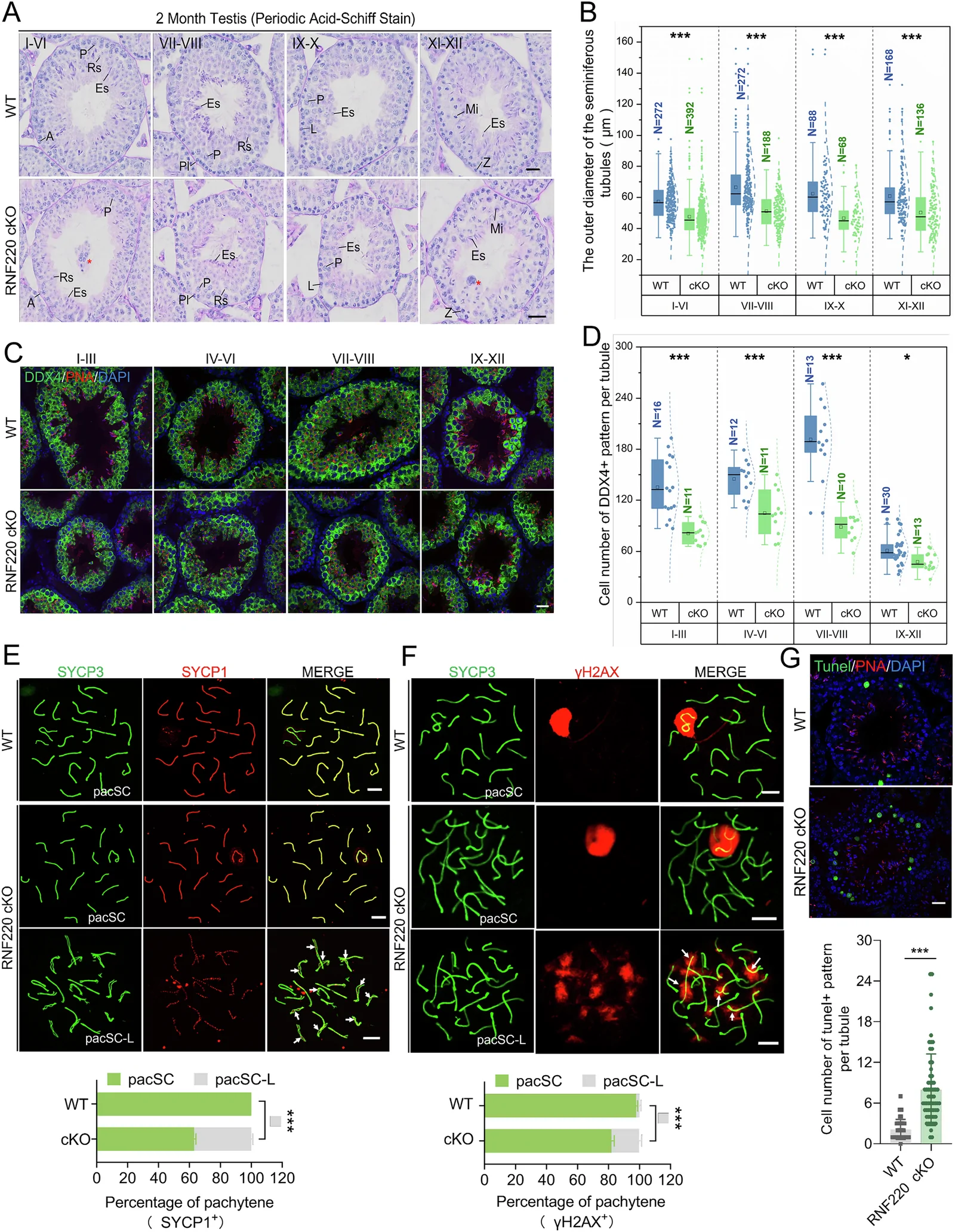
Conditional knockout of *RNF220* disrupts meiotic synapsis, impairs DNA damage repair, and triggers pachytene spermatocyte apoptosis in mice. **A** Representative Periodic Acid-Schiff (PAS) staining images of seminiferous tubules at different stages of the spermatogenic cycle from wild-type (WT) and RNF220 cKO mice. Type A spermatogonia **A**, preleptotene spermatocytes (Pl), leptotene spermatocytes (L), zygotene spermatocytes (Z), pachytene spermatocytes (P), round spermatids (Rs), and elongated spermatids (Es) are indicated. Red asterisks indicate abnormal cell aggregates in the cKO group. Scale bar: 50 μm. **B** Outer diameter of seminiferous tubules at different stages of the spermatogenic cycle from WT and RNF220 cKO mice. (*n* = 3 mice; *N*, counted seminiferous tubules). ***P < 0.001. **C** Representative immunofluorescence staining of DDX4 in seminiferous tubules at different stages of the spermatogenic cycle from WT and RNF220 cKO mice. Triple staining: PNA (acrosome, red), DDX4 (germ cells, green), DAPI (nuclei, blue). Scale bar: 20 μm. **D** Number of DDX4-positive germ cells per seminiferous tubule cross-section at different stages of the spermatogenic cycle from WT and RNF220 cKO mice. (*n* = 3 mice; *N*, counted seminiferous tubules). *P < 0.05, ***P < 0.001. **E** Upper panels: Representative immunofluorescence staining of chromosome synapsis by spermatocyte nuclear spread assay from WT and RNF220 cKO mice. SYCP3 (lateral elements of the synaptonemal complex, green), SYCP1 (transverse filaments of the synaptonemal complex, red). Arrows indicate abnormal synapsis. Scale bar: 5 μm. Lower panel: Quantification of the percentage of SYCP1⁺ pachytene spermatocytes showing normal (pacSC, green) or abnormal (pacSC-L, gray) synapsis. (*n* = 3 mice; counted pachytene spermatocytes (*N*): WT1 = 102, cKO1 = 108; WT2 = 110, cKO2 = 92; WT3 = 111, cKO3 = 112). ***P < 0.001 (WT vs. cKO, % abnormal synapsis). **F** Upper panels: Representative immunofluorescence staining of DNA double-strand breaks (DSBs) by spermatocyte nuclear spread assay from WT and RNF220 cKO mice. SYCP3 (lateral elements of the synaptonemal complex, green), γH2AX (DSB sites, red). Arrows indicate unrepaired DSBs. Scale bar: 5 μm. Lower panel: Quantification of the percentage of γH2AX⁺ pachytene spermatocytes with repaired (pacSC, green) or unrepaired (pacSC-L, gray) DSBs. (*n* = 3 mice; counted pachytene spermatocytes (*N*): WT1 = 115, cKO1 = 131; WT2 = 127, cKO2 = 149; WT3 = 118, cKO3 = 139). ***P < 0.001 (WT vs. cKO, % unrepaired DSBs). **G** Upper panels: Representative TUNEL staining for apoptosis in testicular tissue from WT and RNF220 cKO mice. TUNEL (apoptotic cells, green), PNA (acrosome, red), DAPI (nuclei, blue). Scale bar: 20 μm. Lower panel: Quantification of TUNEL-positive apoptotic cell number per seminiferous tubule cross-section. (*n* = 3 mice; counted seminiferous tubules (*N*): WT = 45, cKO=65). ***P < 0.001 (WT vs. cKO).

To investigate the underlying causes of impaired male fertility in *RNF220* knockout mice, we first performed morphological analysis of adult testicular tissues. Periodic acid-Schiff (PAS) staining revealed no overt spermatogenic arrest in the cKO testis, as all spermatogenic cell types remained present (Fig. 3A). However, the radii of the seminiferous tubules were significantly diminished at all stages (Fig. 3B).

To identify the specific cell types affected, we performed spermatogenic wave staging using the acrosome marker peanut agglutinin (PNA) combined with co-staining analysis for different cellular markers. DDX4 (DEAD-box helicase 4) is a canonical marker for germ cells, PLZF (promyelocytic leukemia zinc finger protein) specifically marks spermatogonial stem cells, and WT1 (Wilms tumor protein 1) is a well-characterized marker for Sertoli cells. The results demonstrated a significant reduction in the number of germ cells (DDX4-positive) across all spermatogenic stages in cKO mice (Fig. 3C, D). The populations of spermatogonial stem cells (PLZF-positive) and Sertoli cells (WT1-positive) remained unchanged (Supplementary Fig. S3A–D).

We next employed spermatocyte nuclear spreading assays to closely examine two key meiotic events: chromosomal synapsis and DNA damage repair (Fig. 3E, F). The meiotic prophase I stages (leptotene, zygotene, pachytene, and diplotene) can be distinguished by co-immunostaining of SYCP3 and SYCP1 [37]. The results revealed the presence of pachytene spermatocytes (pacSC) and pachytene-like spermatocytes (pacSC-L) in RNF220 cKO mice. In these pacSC-L cells, although chromosomes were condensed, SYCP1 signals appeared punctate rather than the continuous linear pattern observed in wild-type controls, indicating severe synaptic defects (Fig. 3E, upper panel). Quantification revealed approximately 37% of pachytene spermatocytes from RNF220 cKO mice exhibited defective synapsis, a proportion significantly higher than WT controls (Fig. 3E, lower panel). During prophase I of meiosis, homologous chromosome synapsis is accompanied by DNA double-strand break (DSB) repair, leading to phosphorylation of H2AX into γH2AX. shows diffuse nuclear staining in leptotene and zygotene spermatocytes; following DSB repair, γH2AX signals become restricted to the sex chromosomes in pachytene and diplotene spermatocytes [38, 39]. In the pachytene-like spermatocytes, however, γH2AX signals were ectopically detected on autosomes in addition to sex chromosomes (Fig. 3F, upper panel), indicating defective DSB repair. Quantification revealed approximately 18% of pachytene spermatocytes from RNF220 cKO mice displayed defective DSB repair, a proportion significantly higher than WT controls (Fig. 3F, lower panel). In contrast, spermatocytes at other meiotic stages appeared largely normal (Supplementary Fig. S3E, F). TUNEL (terminal dUTP nick-end labeling) apoptosis assays, validated by positive and negative controls (Supplementary Fig. S4A), revealed abundant apoptotic cells in cKO testes, with apoptotic signals mainly detected in pachytene spermatocytes (Fig. 3G).

These findings collectively demonstrate that RNF220 is essential for maintaining normal chromosomal synapsis and DNA damage repair during meiosis, and its deficiency may activate the pachytene checkpoint, leading to extensive apoptosis of spermatocytes.

### *RNF220* deficiency affects sperm chromatin condensation

**Fig. 4:**
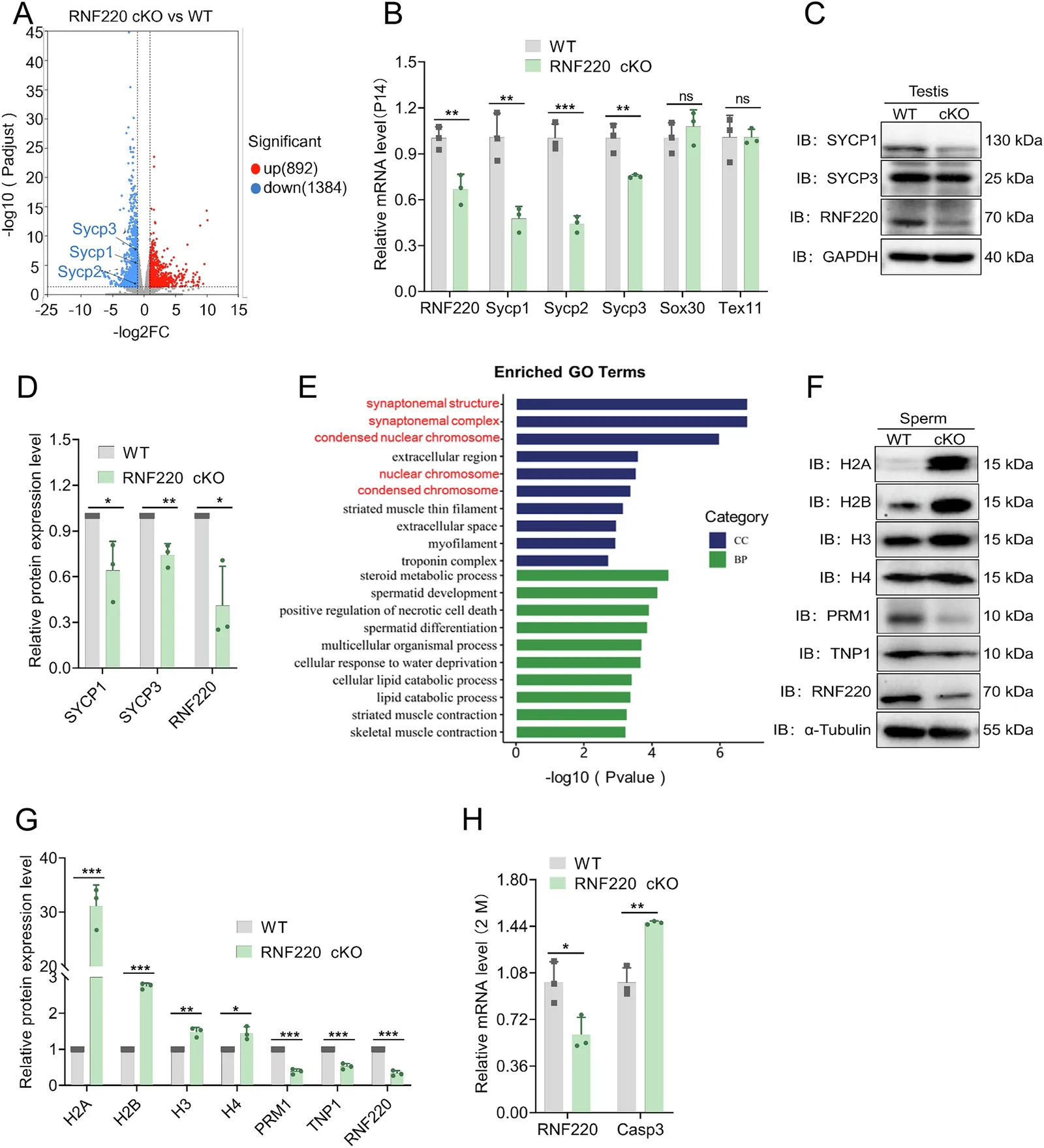
RNA-seq analysis and validation of gene expression changes in RNF220 cKO mice. **A** Volcano plot of differentially expressed genes (DEGs) from RNA-seq analysis of P14 mouse testes in RNF220 cKO versus wild-type (WT) mice. Red dots indicate significantly upregulated genes (*n* = 892), and blue dots indicate significantly downregulated genes (*n* = 1384). **B** Relative mRNA expression levels of *RNF220*, *Sycp1*, *Sycp2*, *Sycp3*, *Sox30*, and *Tex11* in P14 testes validated by RT-qPCR in WT and RNF220 cKO mice. Expression in WT mice was set as 1 (*n* = 3 mice). **P < 0.01; ***P < 0.001; ns, not significant. **C** Representative Western blotting analysis of SYCP1 and SYCP3 protein expression in P14 testes from WT and RNF220 cKO mice. GAPDH was used as the loading control. **D** Quantification of SYCP1, SYCP3 and RNF220 protein levels in testes from 2-month-old WT and RNF220 cKO mice (WB densitometry from Fig. 4C), with WT expression set as 1 (*n* = 3 mice). *P < 0.05, **P < 0.01. **E** Gene Ontology (GO) enrichment analysis of differentially expressed genes (DEGs) from 2-month-old RNF220 cKO versus WT mouse testes. Blue bars represent cellular component (CC) terms, and green bars represent biological process (BP) terms. **F** Representative Western blotting analysis of core histones (H2A, H2B, H3, H4), transition protein 1 (TNP1), protamine 1 (PRM1), and RNF220 protein expression in sperm from 2-month-old WT and RNF220 cKO mice. α-Tubulin was used as the loading control. **G** Quantification of histone and spermiogenesis-related protein levels in mature sperm from 2-month-old WT and RNF220 cKO mice (WB densitometry from Fig. 4-F), with WT expression set as 1 (*n* = 3 mice). *P < 0.05, **P < 0.01, ***P < 0.001. **H** Relative mRNA expression levels of *RNF220* and *Casp3* in 2-month-old testes validated by RT-qPCR in WT and RNF220 cKO mice. Expression in WT mice was set as 1 (*n* = 3 mice). *P < 0.05; **P < 0.001. **Note:** Residual RNF220 bands observed in cKO testis **C** and sperm **E** samples are derived from somatic cells not targeted by Stra8-Cre-mediated germ cell-specific knockout.

*RNF220* knockout resulted in abnormal homologous chromosome synapsis and DNA damage repair in pachytene spermatocytes. To reveal the underlying molecular mechanism, we performed transcriptome sequencing on testes of wild-type and RNF220 cKO mice at postnatal day 14 (P14, pachytene stage). RNA-seq analysis showed that 1384 genes were significantly downregulated and 892 genes were significantly upregulated in cKO mouse testes compared with controls (|log₂FC | > 1, *P* < 0.05) (Fig. 4A). Among these differentially expressed genes, we identified the core synaptonemal complex components *Sycp1*, *Sycp2* and *Sycp3*. Consistent with the transcriptome data, RT‑qPCR analysis showed that the mRNA levels of *Sycp1*, *Sycp2* and *Sycp3* were significantly decreased in RNF220 cKO testes (Fig. 4B), whereas the expression levels of the meiotic regulators *Sox30* and *Tex11* remained unchanged at this stage (Fig. 4B). We further confirmed by Western blotting that the protein expression levels of SYCP1 and SYCP3 were significantly reduced in cKO testes (Figs. 4C, D).

In addition, RNF220 cKO mice also exhibited abnormal sperm morphology. Therefore, we performed transcriptome sequencing again on testes of 2-month-old (sexually mature) mice. The results showed that 110 genes were significantly downregulated and 133 genes were significantly upregulated in cKO mouse testes (|log₂FC | > 1, *P* < 0.05). GO enrichment analysis revealed that the differentially expressed genes were significantly enriched not only in pathways related to chromosome synapsis, but also in pathways associated with chromatin condensation (Fig. 4E). Given that the replacement of histones by protamines is a key step in chromatin condensation [40], we further examined the expression levels of related proteins in cauda epididymal sperm, the purity of which was microscopically validated (Supplementary Fig. S5). Western blot analysis showed that the expression of core histones (H2A, H2B, H3, and H4) was significantly increased, whereas the expression of transition protein TNP1 and protamine PRM1 was significantly decreased in cKO mouse testes (Figs. 4F, G), suggesting that the process of chromatin condensation was severely impaired. Meanwhile, we performed RT-qPCR on testes from 2-month-old mice and found that the mRNA expression level of *Casp3* was significantly increased (Fig. 4H), consistent with the TUNEL results.

In summary, *RNF220* deficiency also impairs chromatin condensation during spermiogenesis.

### RNF220 antagonizes K48-linked ubiquitination and degradation of SYCE1

**Fig. 5:**
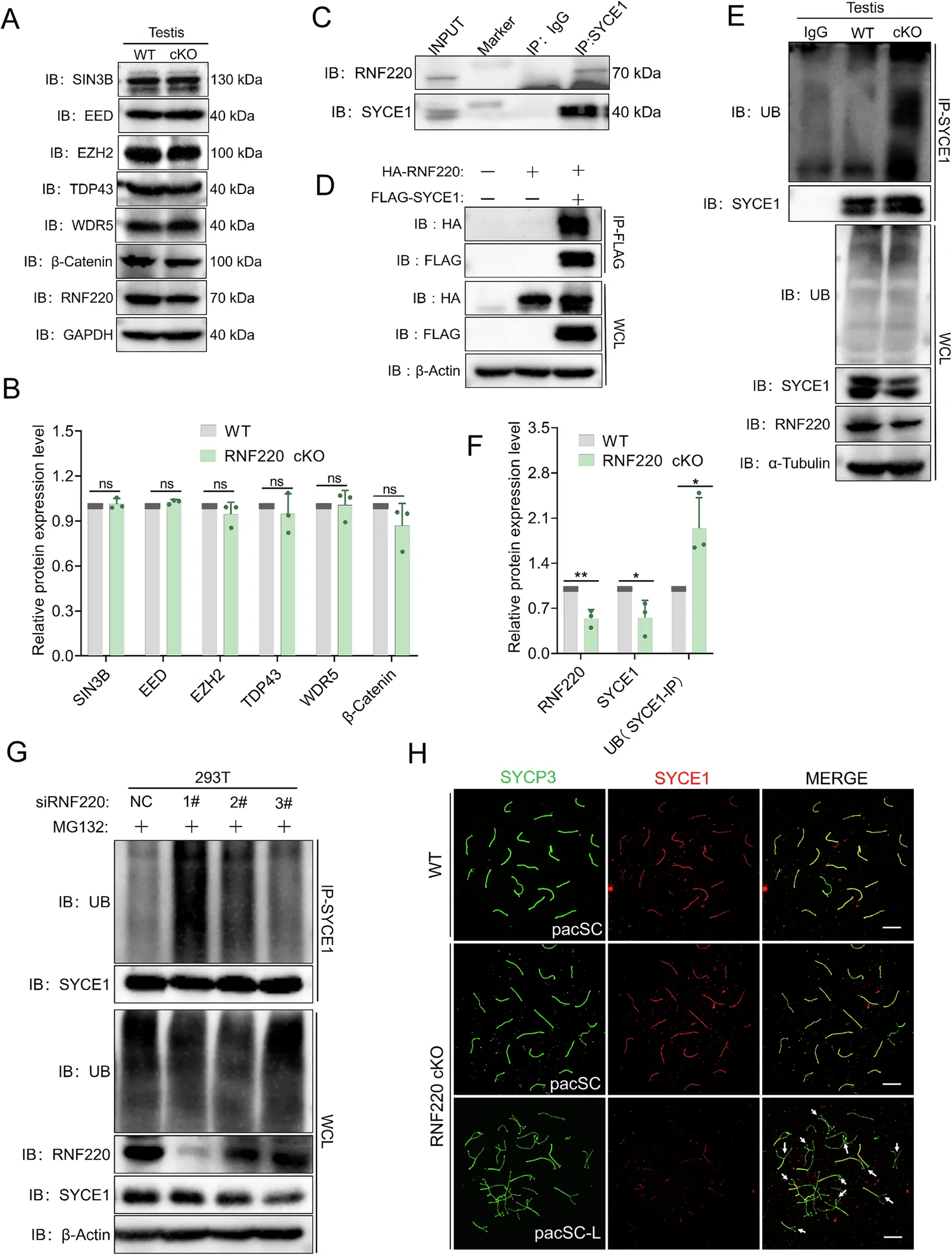
RNF220 interacts with SYCE1 and stabilizes it. **A** Representative Western blotting analysis of SIN3B, EED, EZH2, TDP43, WDR5, β‑catenin, and RNF220 protein expression in testes from 2-month-old wild‑type (WT) and RNF220 cKO mice. GAPDH was used as the loading control. **B** Quantification of known target protein levels in testes from 2-month-old WT and RNF220 cKO mice (WB densitometry from Fig. 5. **A**), with WT expression set as 1 (*n* = 3 mice). ns, no significant difference. **C** Co‑immunoprecipitation (Co‑IP) analysis of the interaction between endogenous RNF220 and SYCE1 in mouse testes. Immunoprecipitation was performed using an SYCE1 antibody, with IgG as the negative control. **D** Co‑immunoprecipitation (Co‑IP) validation of the interaction between RNF220 and SYCE1 in 293 T cells. Cells were co‑transfected with HA‑tagged RNF220 (HA‑RNF220) and Flag‑tagged SYCE1 (Flag‑SYCE1) plasmids, followed by immunoprecipitation with Flag beads. β-Actin was used as the loading control for whole-cell lysates (WCL). **E** Ubiquitination analysis of SYCE1 in testes from 2-month-old wild‑type (WT) and RNF220 cKO mice. Immunoprecipitation (IP) was performed using an SYCE1 antibody, with IgG as the negative control. Upper panels show ubiquitination levels of immunoprecipitated SYCE1; lower panels show protein expression in whole‑cell lysates (WCL), with α‑tubulin as a loading control. **F** Quantification of RNF220, SYCE1 and ubiquitinated SYCE1 (Ub) levels in testes from 2-month-old WT and RNF220 cKO mice (WB densitometry from Fig. 5. **E**), with WT expression set as 1 (*n* = 3 mice). *P < 0.05, **P < 0.01. **G** Ubiquitination analysis of SYCE1 in 293 T cells following *RNF220* knockdown. Cells were transfected with control siRNA (siNC) or RNF220‑targeting siRNA (siRNF220). Immunoprecipitation (IP) was performed using an SYCE1 antibody, with IgG as the negative control. Upper panels show ubiquitination levels of immunoprecipitated SYCE1; lower panels show protein expression levels in whole-cell lysates (WCL), with β-Actin as a loading control. **H** Representative immunofluorescence staining of synapsis by spermatocyte nuclear spread assay from WT and RNF220 cKO mice. SYCP3 (lateral elements of the synaptonemal complex, green), SYCE1 (central element of the synaptonemal complex, red). Arrows indicate unsynapsed chromosomal regions. Scale bar: 10 μm.

As an ubiquitin E3 ligase, RNF220 regulates various developmental processes through different substrates, such as Sin3B, EED, WDR5, and β-catenin [17, 18, 23, 41]. Notably, the level of Sin3B has been suggested to be elevated in human spermatozoa from an infertile patient carrying a *RNF220* mutation [24]. However, the expression levels of the above known target proteins remained unchanged in the RNF220 cKO testis (Figs. 5A, B). The synaptonemal complex central element protein SYCE1 has been identified as an interactor of zinc finger C4H2-type containing protein (ZC4H2) [42, 43], which is a binding partner of RNF220 [44]. We therefore examined the interaction between RNF220 and SYCE1 both in vivo and in vitro. Co‑immunoprecipitation (Co‑IP) assays verified their endogenous interaction in mouse testis (Fig. 5C). Consistently, in vitro overexpression Co‑IP using 293 T cells further confirmed this binding, as SYCE1 efficiently pulled down RNF220 (Fig. 5D). Interestingly, the protein level of SYCE1 was reduced in the whole cell lysates of the RNF220 cKO testes, while the polyubiquitination level of SYCE1 was markedly increased (Figs. 5E, 5F). Knocking down RNF220 in 293 T cells also enhanced SYCE1 ubiquitination (Fig. 5G). Spermatocyte nuclear spreading staining showed that, in the synapsis-abnormal pachytene-like spermatocytes, the expression of SYCE1 was greatly reduced (Fig. 5H).

**Fig. 6:**
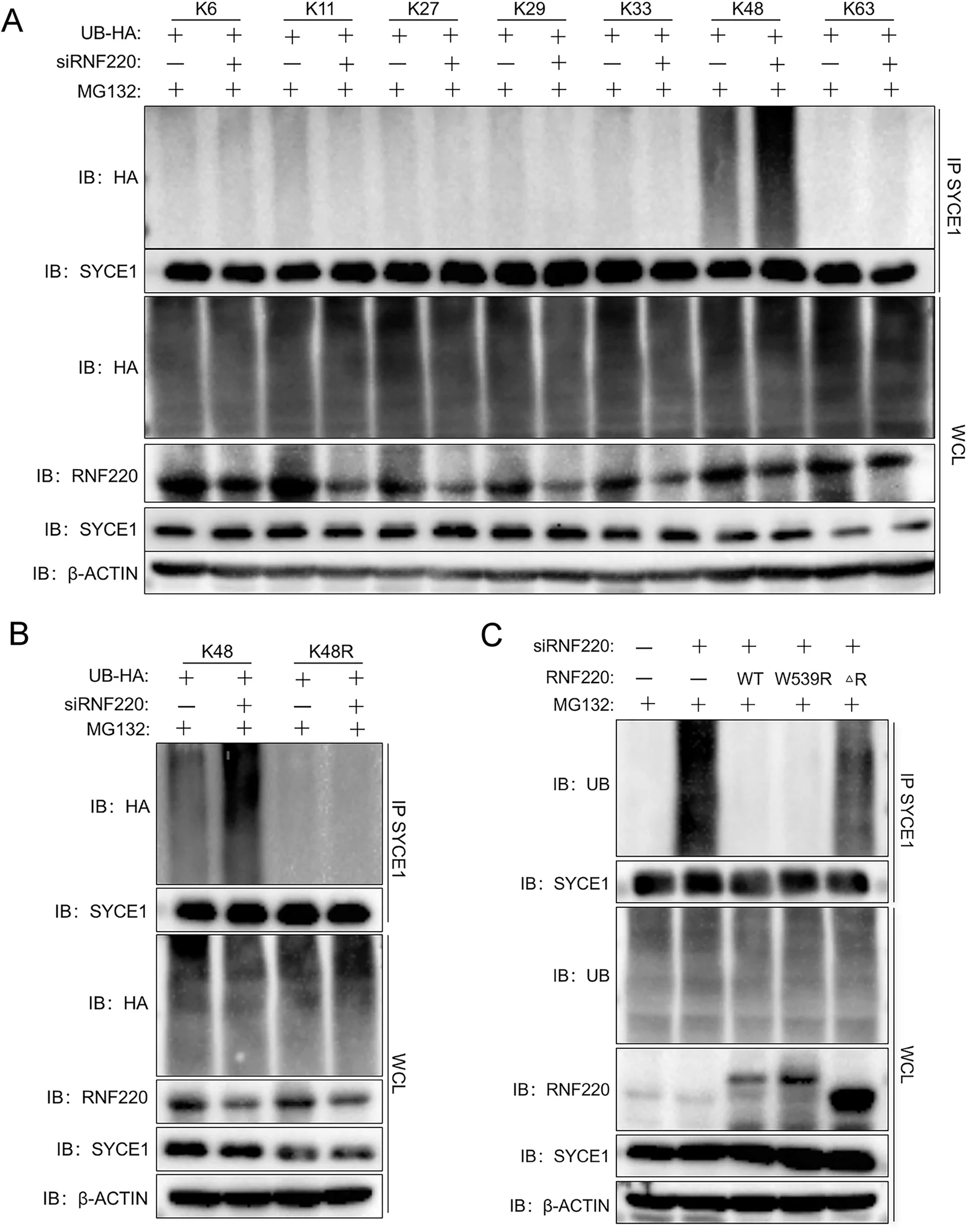
RNF220 antagonizes K48-linked ubiquitination and degradation of SYCE1 via its RING domain. **A** Ubiquitin chain linkage analysis of SYCE1 in 293 T cells. Cells were co-transfected with HA-tagged ubiquitin K-only mutants (K6, K11, K27, K29, K33, K48, K63) along with control siRNA (siNC) or RNF220-targeting siRNA (siRNF220), and treated with MG132. Immunoprecipitation (IP) was performed using a SYCE1 antibody, followed by immunoblotting (IB) with a HA antibody to detect specific ubiquitin chain types. β-Actin was used as the loading control. **B** Ubiquitination analysis of SYCE1 in 293 T cells transfected with HA-tagged ubiquitin (K48) or the K48R mutant, together with siNC or siRNF220. IP was performed using a SYCE1 antibody, and IB was detected with a HA antibody. β-Actin served as the loading control. **C** Functional domain analysis of RNF220 in SYCE1 ubiquitination. 293 T cells were transfected with siNC or siRNF220, and reconstituted with empty vector, wild-type RNF220 (RNF220-WT), RNF220-W539R, or RNF220-ΔRING. IP was performed using a SYCE1 antibody, and IB was detected with a HA antibody. β-Actin was used as the loading control.

Ubiquitination ligases can add different types of ubiquitin chains to their target proteins, with K48-linked chains mostly proteolytic, K63 and other lysine linked chains mostly nonproteolytic [45]. We examined the ubiquitin chain type in SYCE1 using single lysine-only or single lysine to arginine (KR) mutants of HA-tagged ubiquitin in 293 T cells. We observed that the K48-only ubiquitin, but not other mutants was efficiently incorporated into ubiquitinated SYCE1, which was further increased in RNF220 knockdown cells (Fig. 6A). Conversely, the K48-R mutant of ubiquitin failed to support the increase of SYCE1 ubiquitylation in RNF220 knockdown cells (Fig. 6B). These data indicate that SYCE1 is mostly ubiquitinated by K48 polyubiquitin chains, which most likely lead to proteasomal degradation of SYCE1.

The above data suggest that SYCE1 is likely ubiquitinated by another endogenous E3 ligase that is inhibited by RNF220. We tested whether the E3 ligase activity of RNF220 is required to inhibit SYCE1 ubiquitination. In cells with endogenous RNF220 knocked-down, re-introduction of wild-type RNF220 (RNF220-WT) effectively reversed the excessive ubiquitination of SYCE1 (Fig. 6C, lane 3). Crucially, re-introduction of the E3 ligase activity-dead mutant (RNF220-W539R) also inhibited SYCE1 ubiquitination, with an effect comparable to that of the wild-type (Fig. 6C, lane 4). However, the mutant lacking the entire RING domain (RNF220-ΔR) showed weaker protective capacity (Fig. 6C, lane 5). These data showed that the full ability of RNF220 to inhibit SYCE1 ubiquitination depends on the presence of its RING domain but not the intrinsic catalytic activity of this domain.

## Discussion

In this study, we identify RNF220 as a non-canonical stabilizer of SYCE1, essential for meiotic synapsis and male fertility. Its loss leads to synaptic failure, persistent DNA damage, and subsequent apoptosis of pachytene spermatocytes.

SYCE1 is a core component of the central element of the synaptonemal complex (SC) [13]. Mechanistically, RNF220 directly binds SYCE1 and protects it from K48-linked ubiquitination independent of its intrinsic E3 ligase activity. This adaptor role is analogous to RNF220-dependent β-catenin stabilization via USP7 recruitment [23]. Whether RNF220 recruits deubiquitinases to stabilize SYCE1 awaits further investigation. Another possibility is that RNF220 sterically hinders other E3 ligases to prevent SYCE1 ubiquitination and subsequent degradation.

E3 ligases exert versatile essential functions during meiotic progression. In mice, the ubiquitin E3 ligases HEI10 and RNF212B, as well as the SUMO ligase RNF212, are crucial for crossover formation, meiotic recombination and thus proper synapsis [46, 47]. In Drosophila oocytes, the E3 ubiquitin ligase SINA is required for the correct assembly of the SC polycomplex [48]. Here we show that RNF220 ablation triggers severe synapsis disruption at pachytene and germ cell apoptosis, thereby recapitulating the phenotype of *Syce1* haploinsufficient mice [13]. These data suggest that RNF220 sustains the structural integrity of the SC central element via SYCE1 stabilization; whether RNF220 additionally participates in late-stage crossover control remains unresolved.

Whether RNF220 also plays an independent role during spermiogenesis remains an open question. In humans, RNF220 is expressed in the sperm head and flagellum, and mutations in RNF220 have been linked to small‑head sperm morphology and male infertility [24]. In mice, although RNF220 mRNA and protein levels in spermatids were lower than those in spermatogonia and spermatocytes (Figs. 1D, E), RNF220 protein was detected in mature sperm from the cauda epididymidis and was significantly reduced in cKO mice (Fig. 4F). In our cKO mice, we observed sperm malformations (Figs. 2K, L) and defects in chromatin remodeling during spermiogenesis (Fig. 4E). Whether these defects result indirectly from impaired meiotic synapsis and DSB repair, or instead reflect an independent function of RNF220 in spermiogenesis, remains to be determined.

Several limitations of the present study should be acknowledged. First, we verified the regulatory effect of RNF220 on SYCE1 abundance in cKO testes mainly via western blotting. Combining immunohistochemical staining with comprehensive biochemical assays in future work will help dissect how RNF220 modulates SYCE1 stability, subcellular localization and synaptonemal complex dynamics. Second, the endogenous E3 ubiquitin ligases and relevant deubiquitinases governing SYCE1 ubiquitination remain unidentified, precluding a complete mechanistic delineation of RNF220 function, including steric hindrance against competing E3 ligases or deubiquitinase recruitment. Third, RNF220 deletion was achieved exclusively using the Stra8-Cre driver for germ cell-specific ablation. Consequently, RNF220’s physiological function within testicular somatic compartments remains unexplored, and residual RNF220 signals detected in spermatids are attributable to incomplete Cre-mediated knockout, which may attenuate the phenotypic penetrance in mutant animals.

In summary, our study establishes RNF220 as a vital non-canonical regulator of meiotic synapsis and male fertility. By stabilizing SYCE1 independent of its E3 ligase activity, RNF220 safeguards SC structural integrity and meiotic DNA damage repair. These findings enrich our understanding of the ubiquitin-mediated regulatory network underlying mammalian spermatogenesis and provide a novel mechanistic basis for explaining *RNF220* mutation-associated male infertility.

## Supplementary information

Supplementary Materials

Uncropped Western Blots

## Data Availability

All relevant processed data are included in the published article and its Supplementary Information files. Additional supporting data are available from the corresponding authors upon reasonable request.
