# Supplementary material for: RNF220 safeguards meiotic synapsis and male fertility by antagonizing SYCE1 ubiquitination
Source: Cell Death Dis. 2026 Jul 24;17(1):895. doi: 10.1038/s41419-026-09128-8 (PMC13683351; doi:10.1038/s41419-026-09128-8)
Supplement: Supplementary file 1 [file 41419_2026_9128_MOESM2_ESM.pdf]

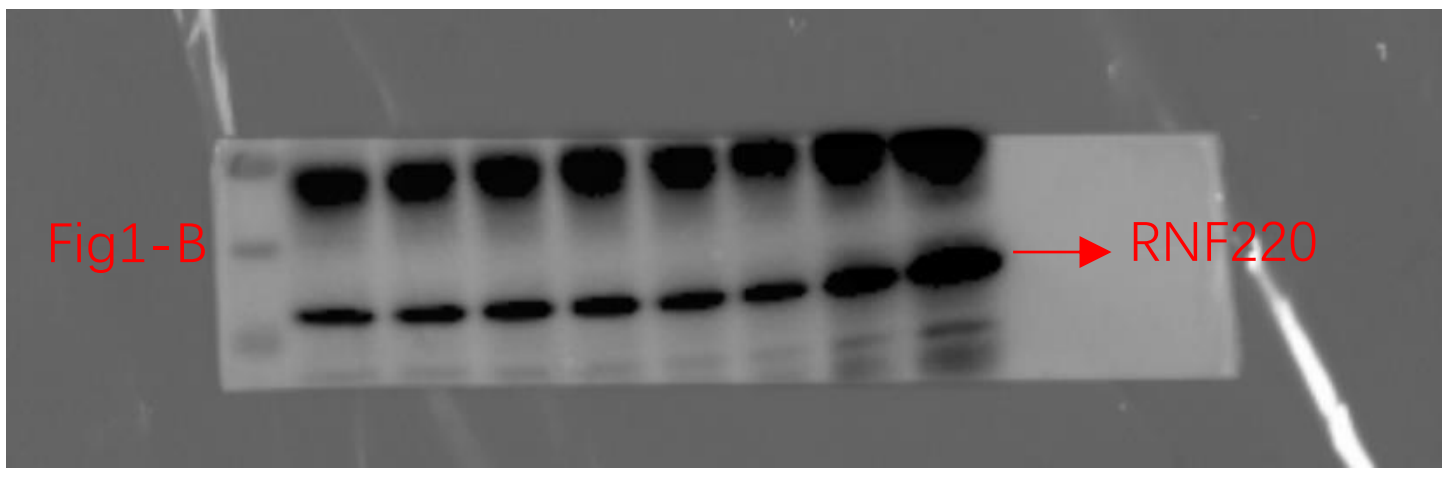
Fig1-B
RNF220

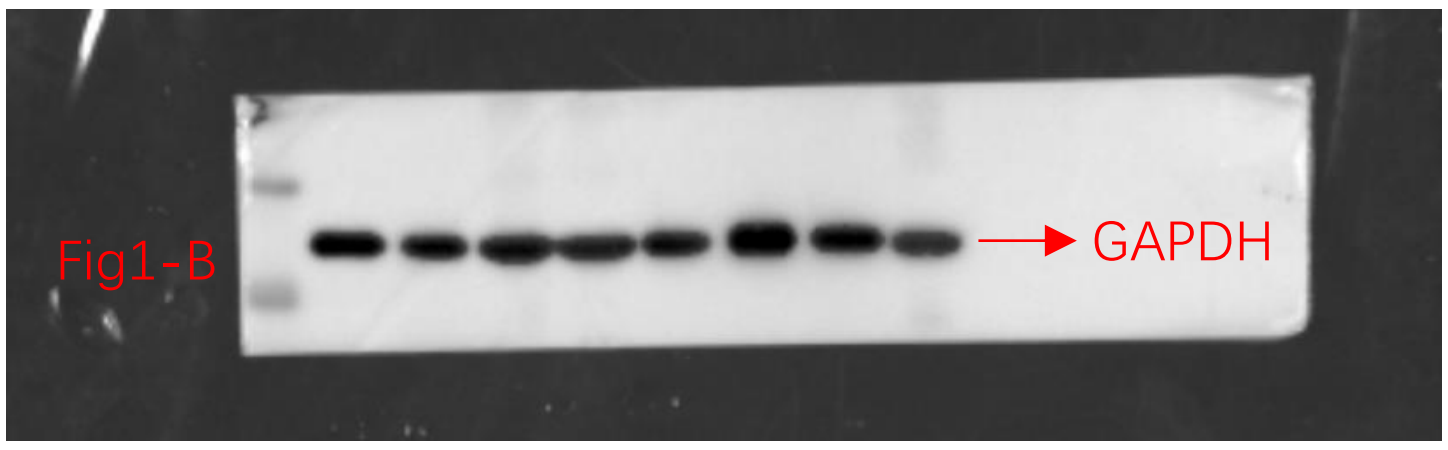
Fig1-B
GAPDH

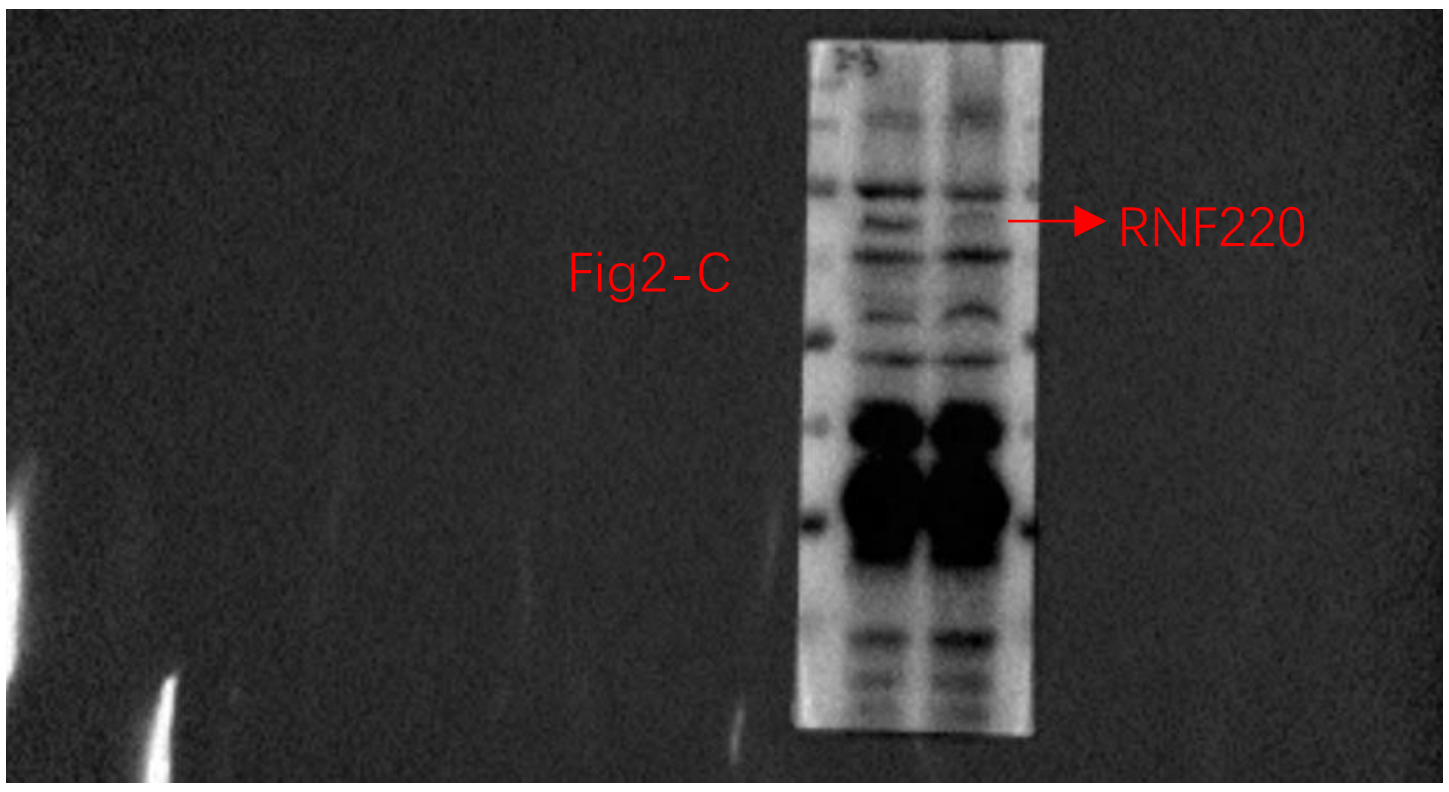
Fig2-C
RNF220

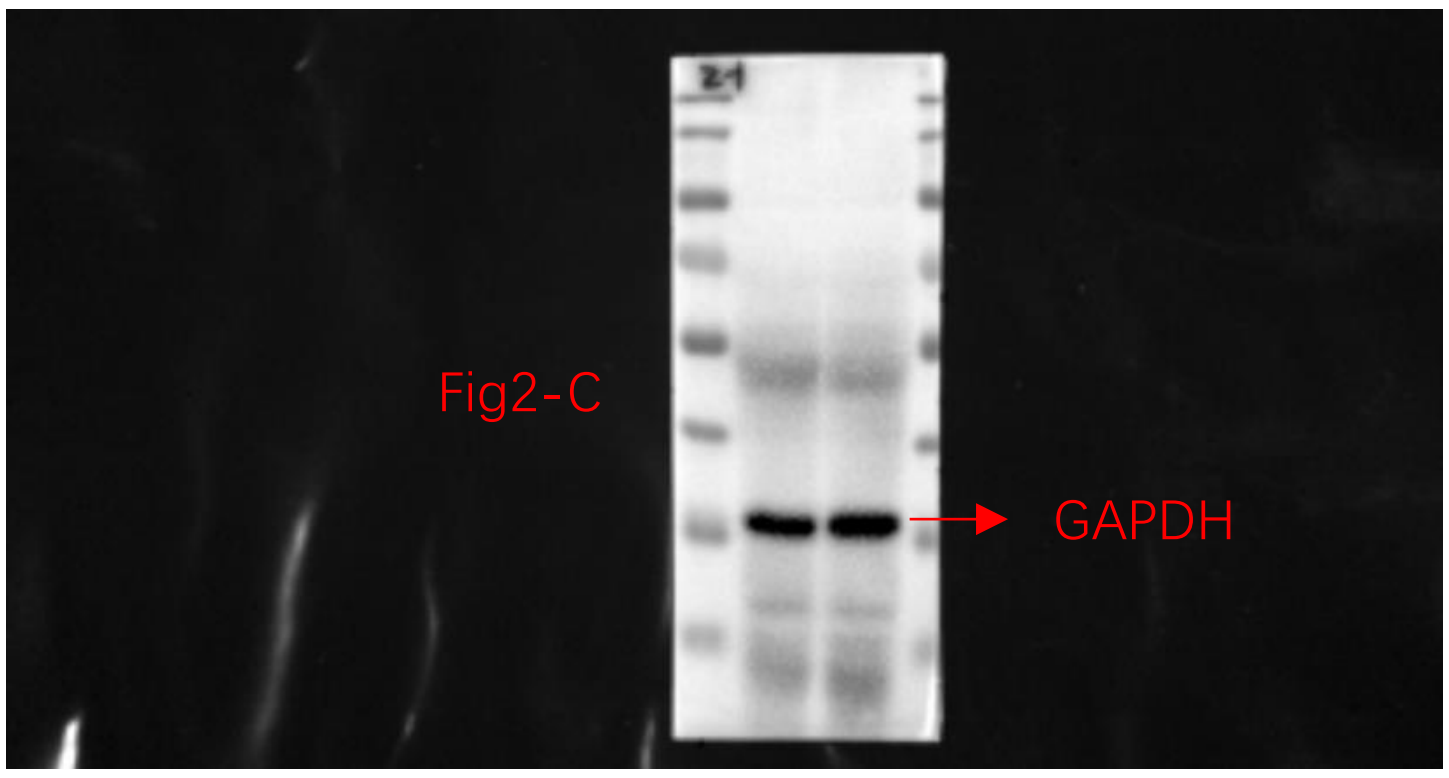
Fig2-C
GAPDH

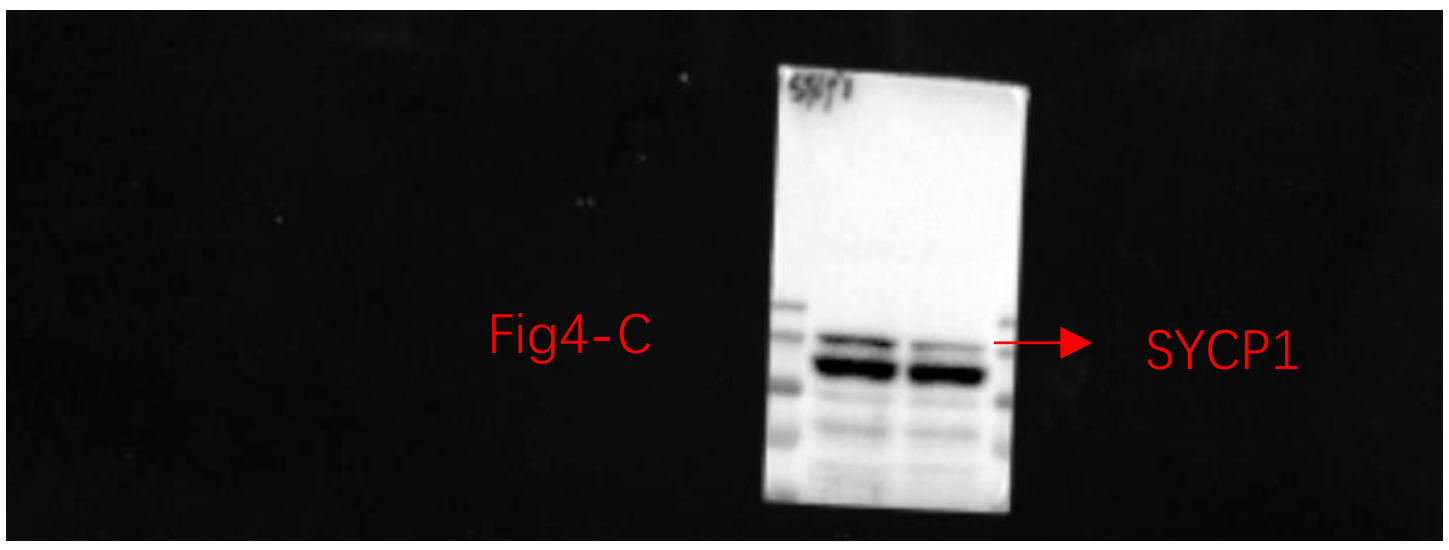
Fig4-C
SYCP1

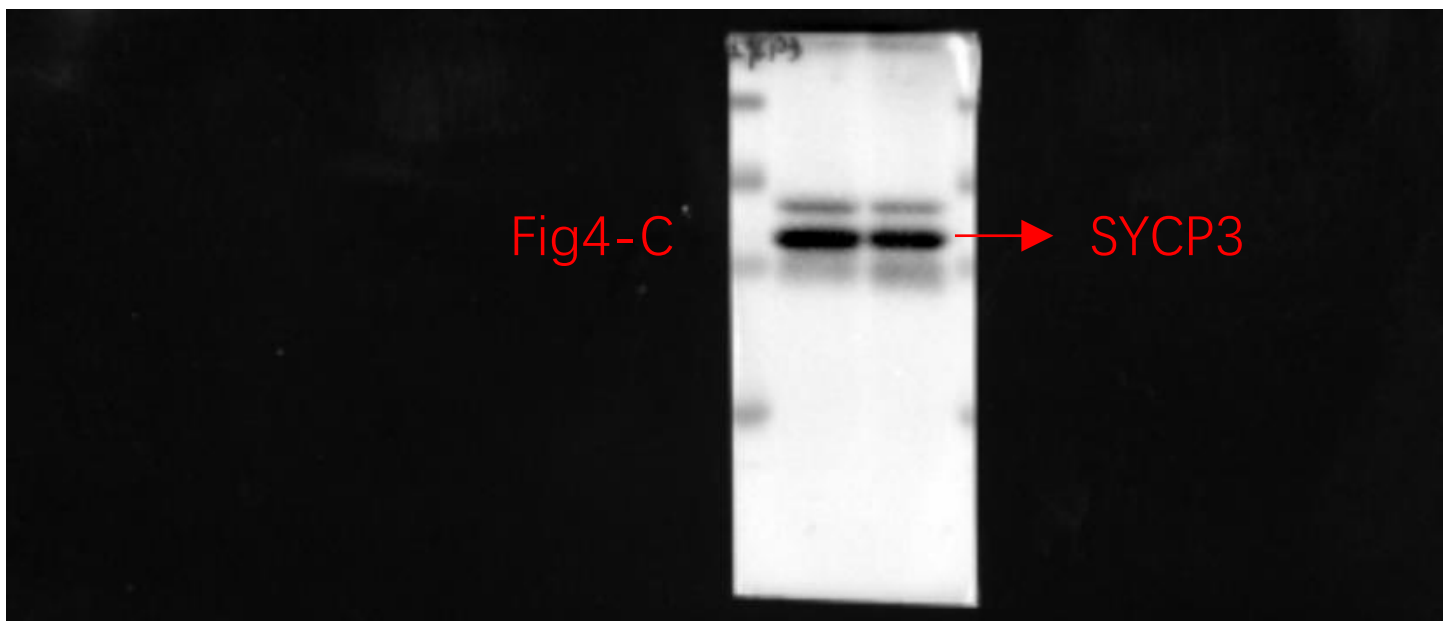
Fig4-C
SYCP3

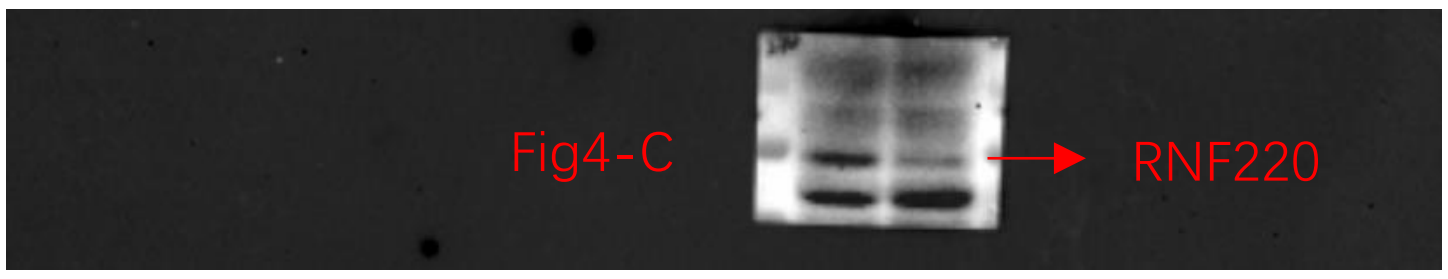
Fig4-C
RNF220

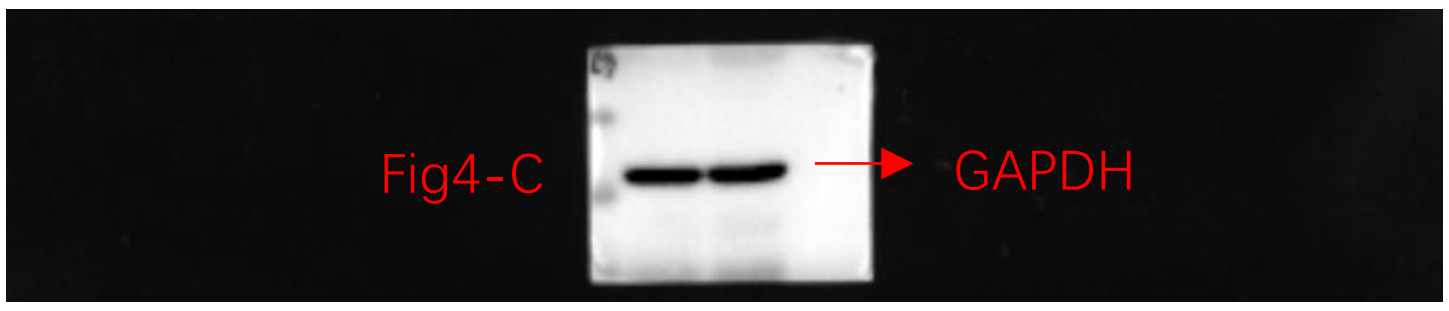
Fig4-C
GAPDH

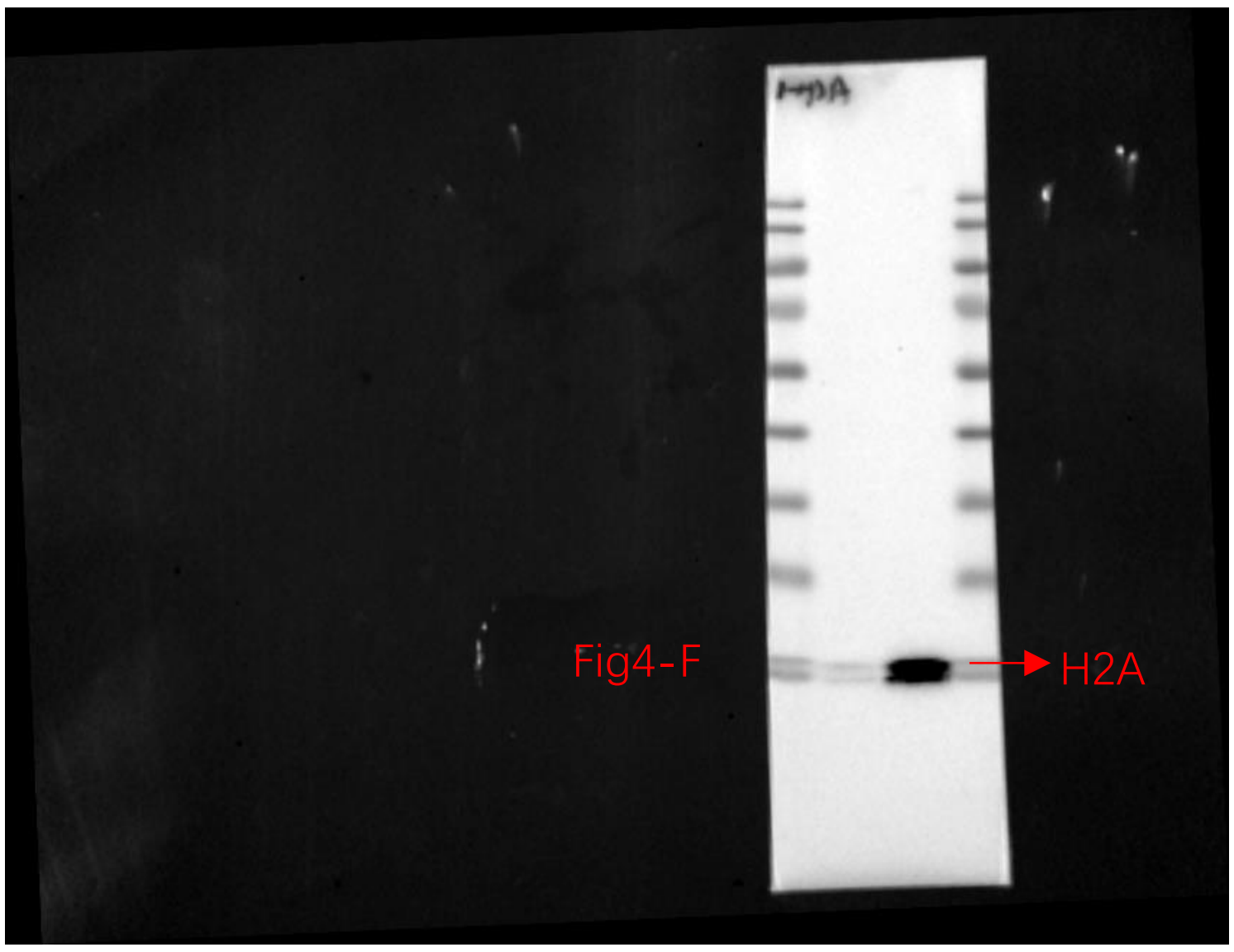
Fig4-F
H2A

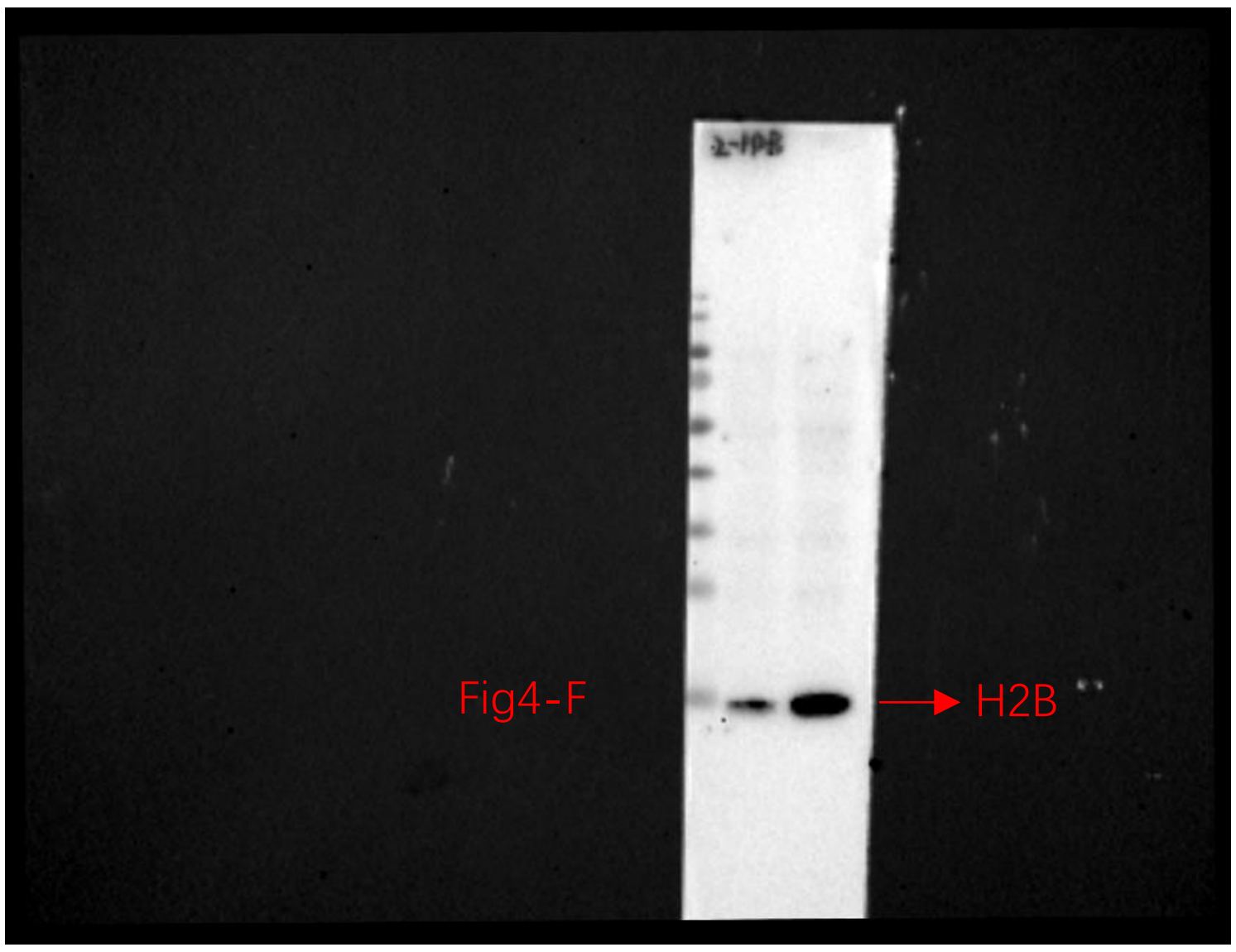
Fig4-F
H2B

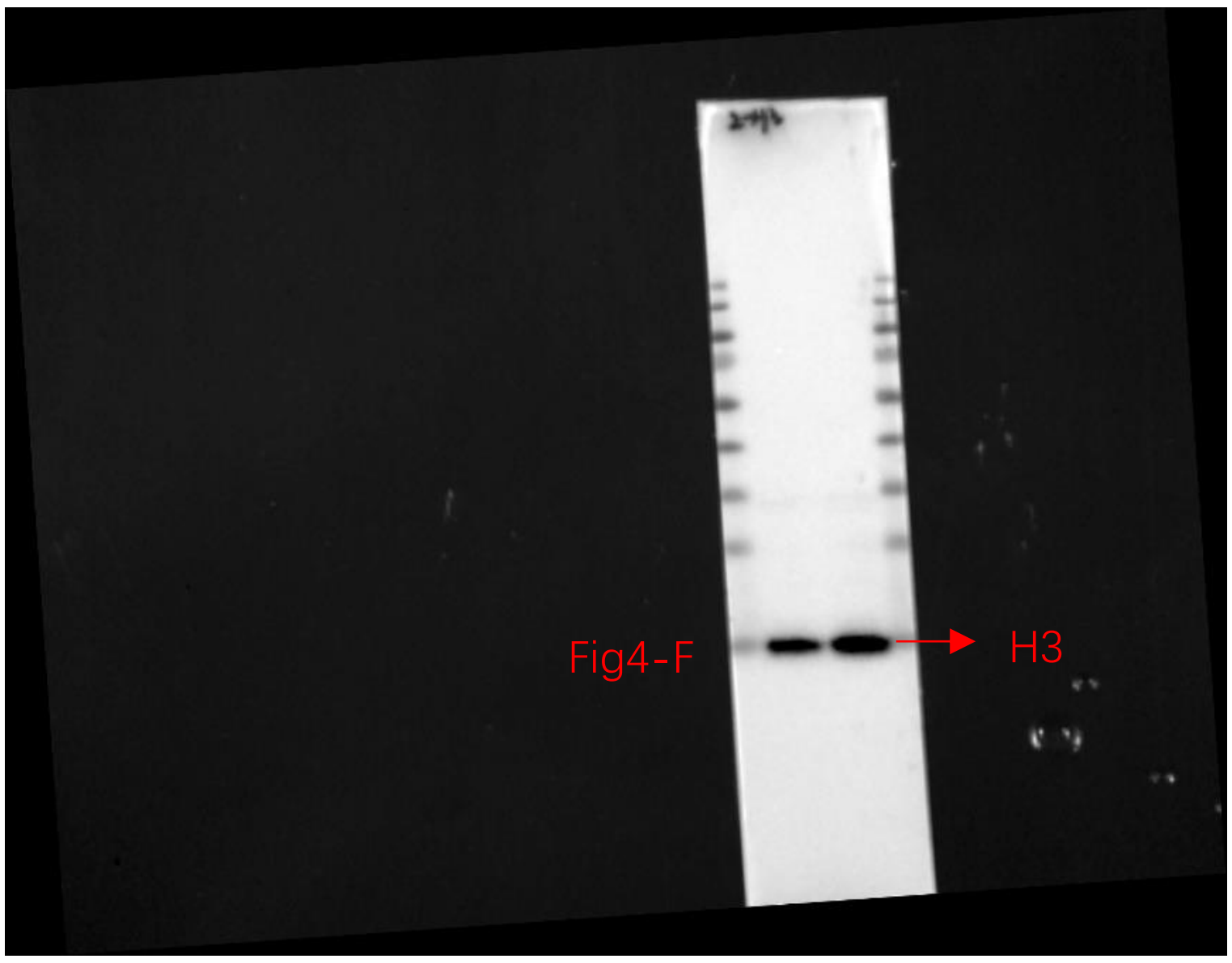
Fig4-F
H3

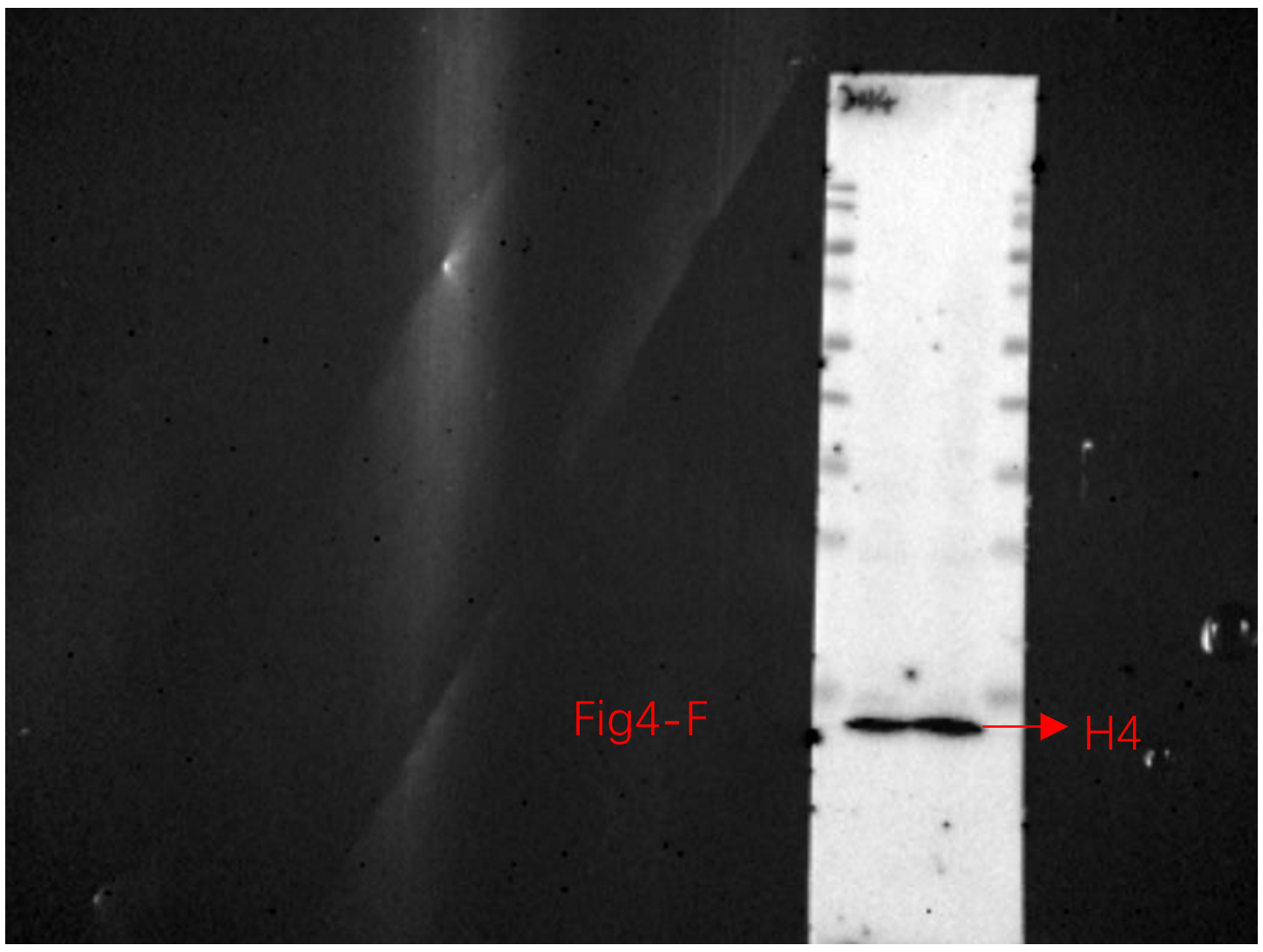
Fig4-F
H4

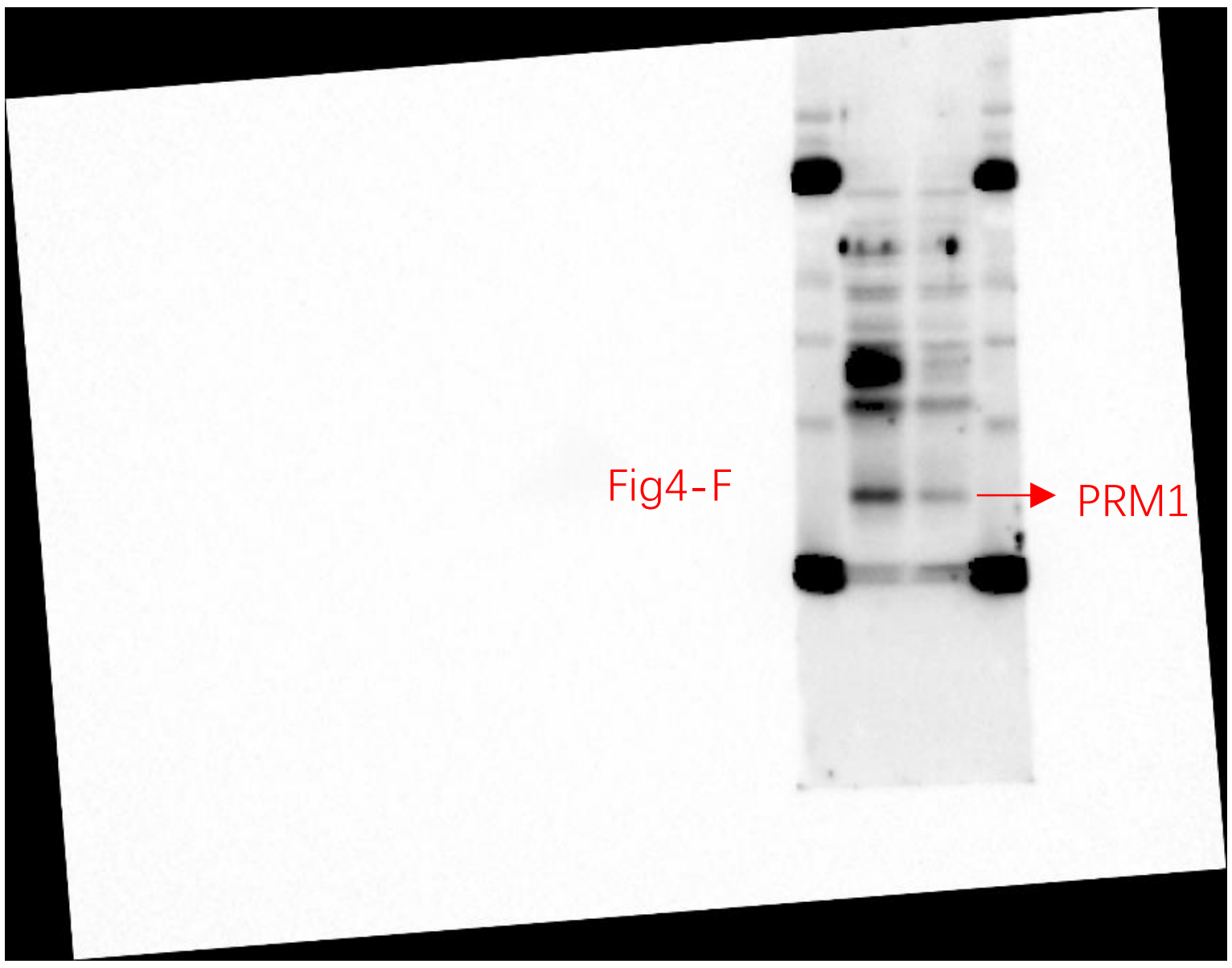
Fig4-F
PRM1

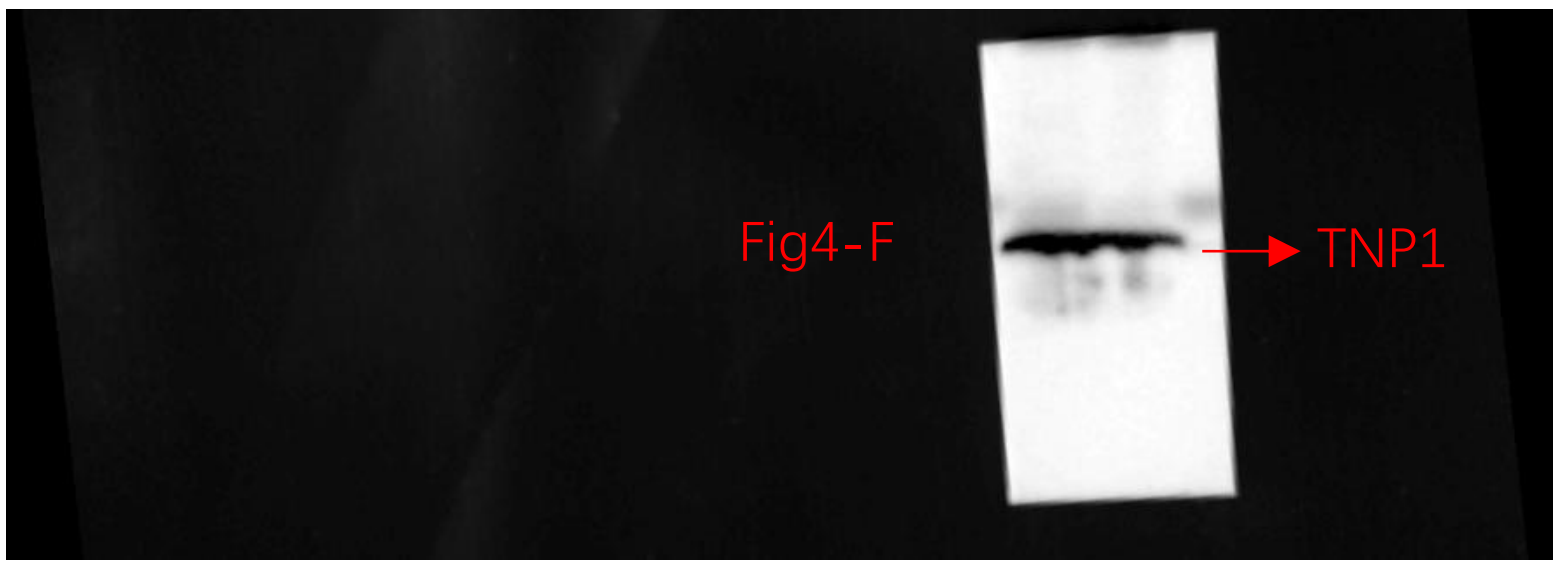
Fig4-F
TNP1

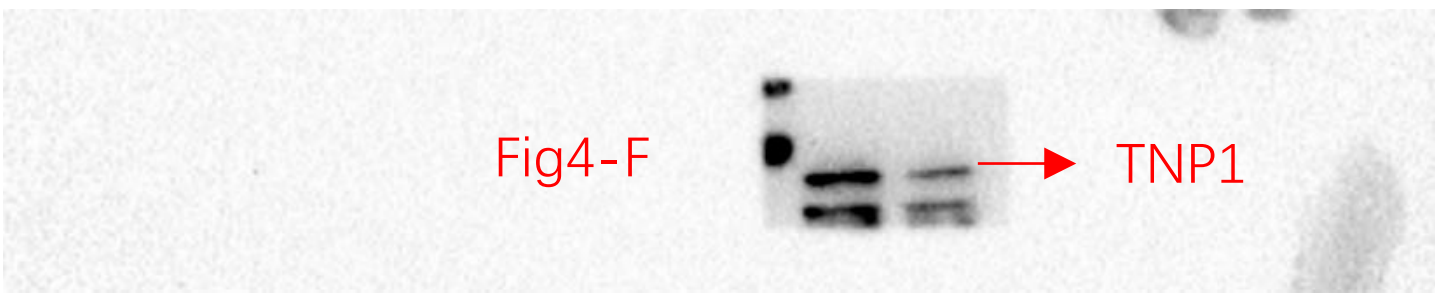
Fig4-F
TNP1

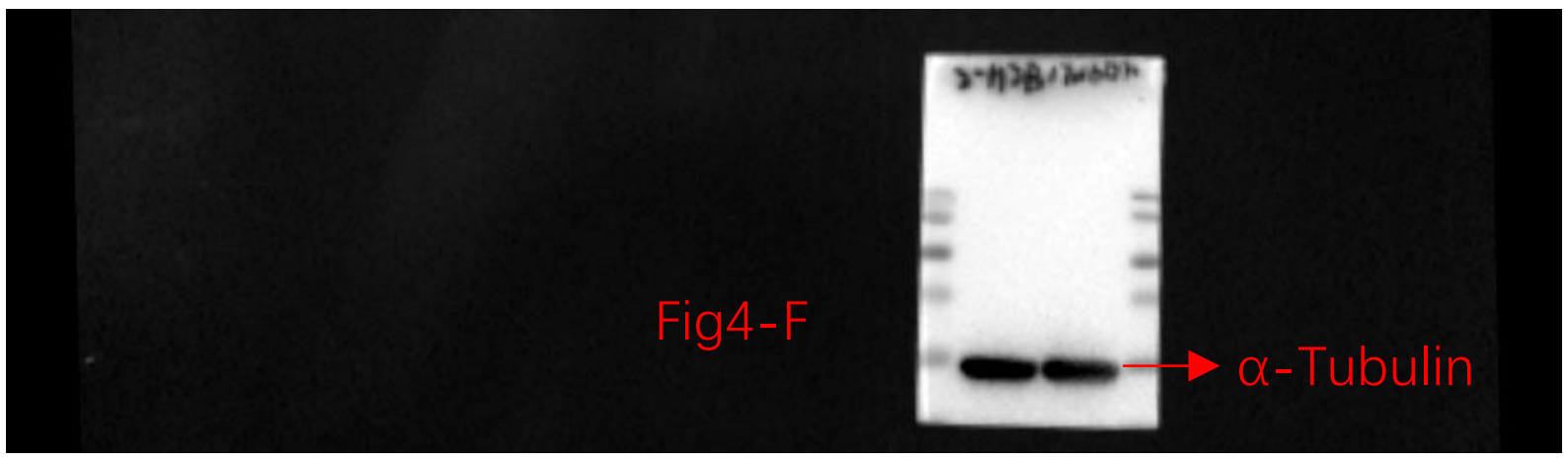
Fig4-F
α-Tubulin

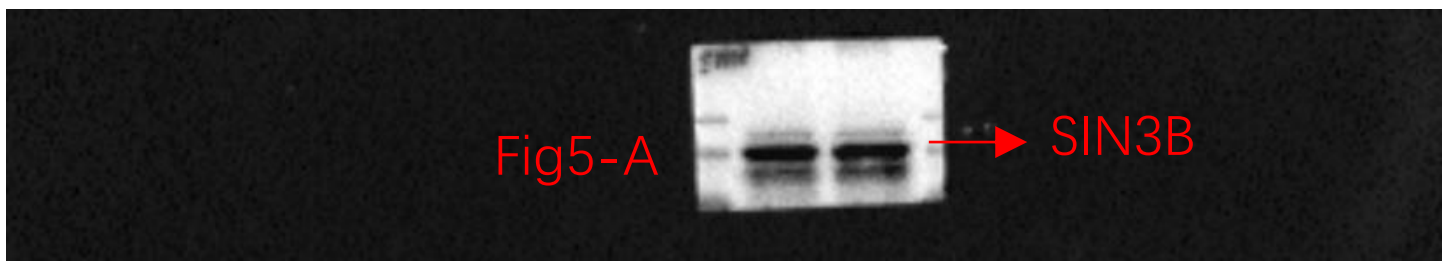
Fig5-A
SIN3B

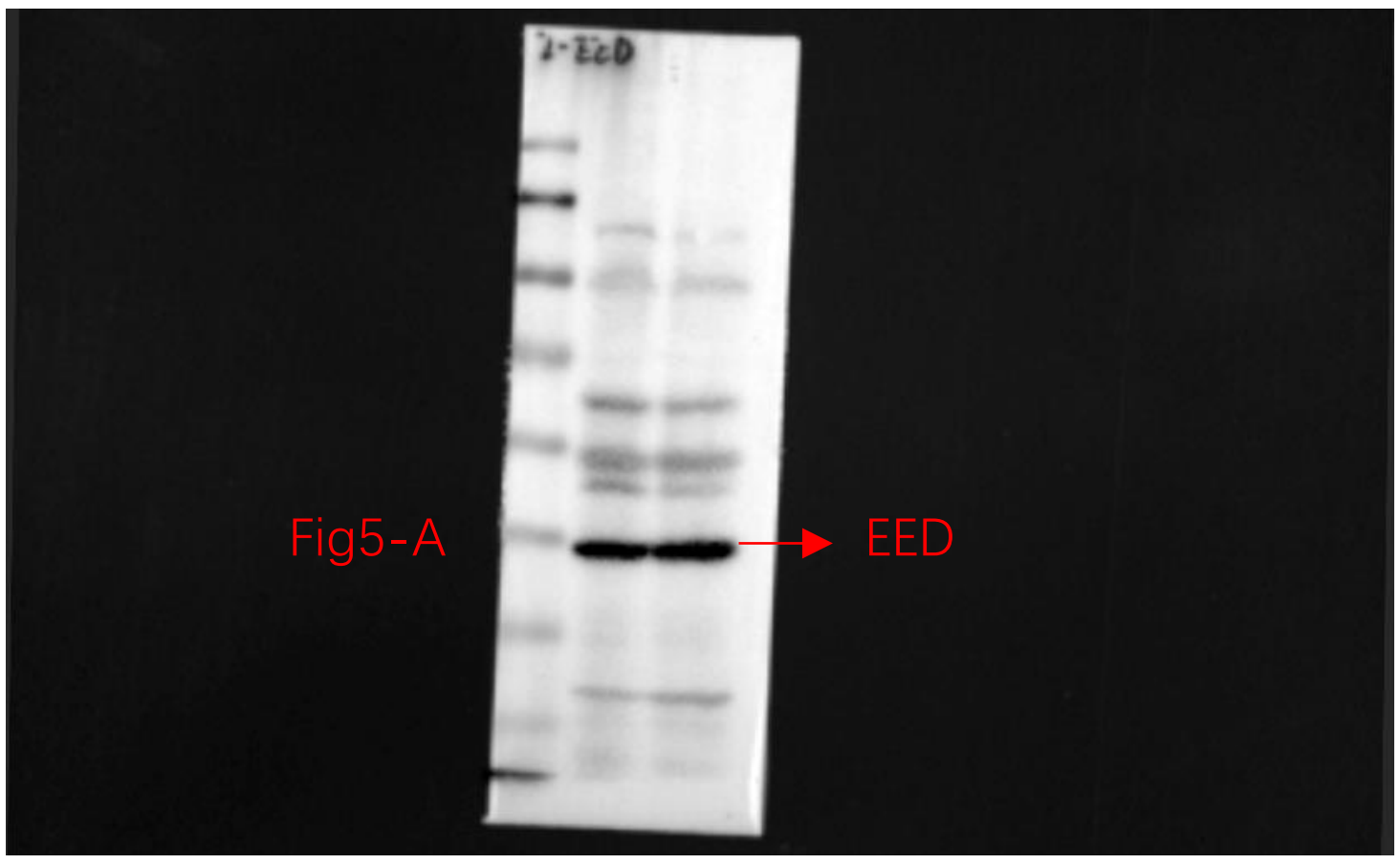
Fig5-A
EED

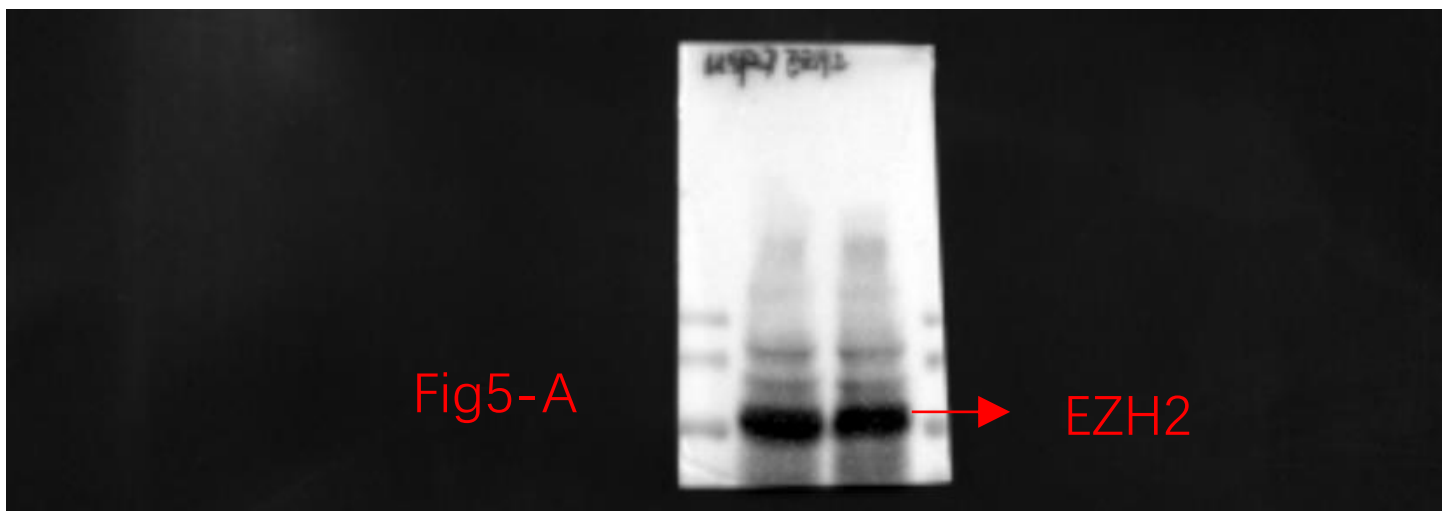
Fig5-A
EZH2

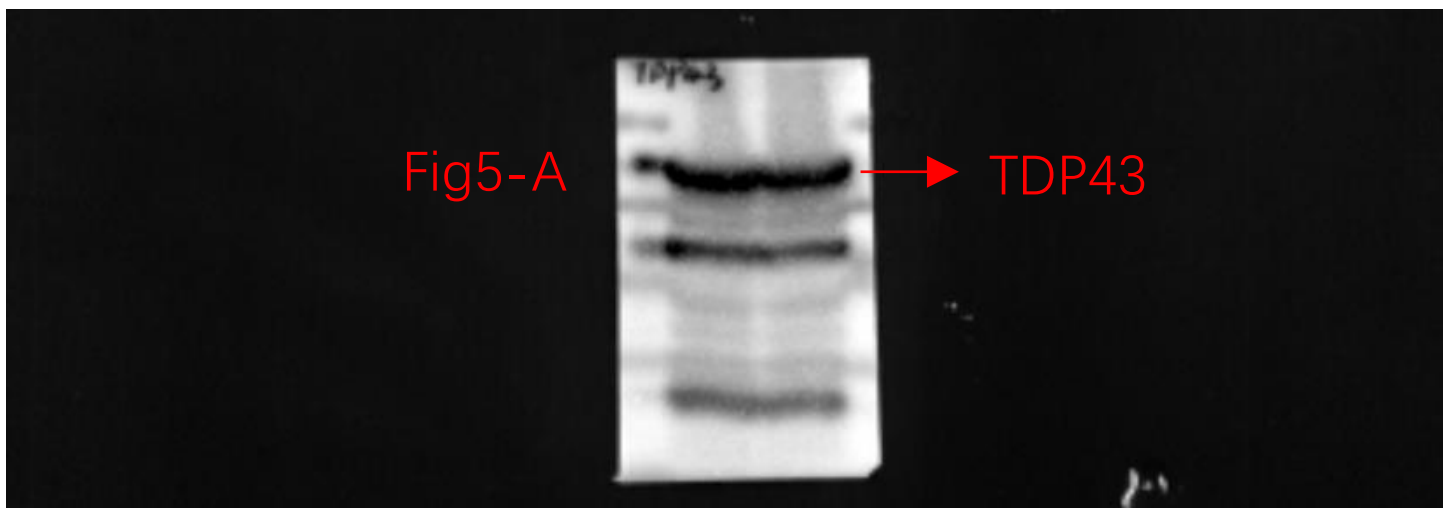
Fig5-A
TDP43

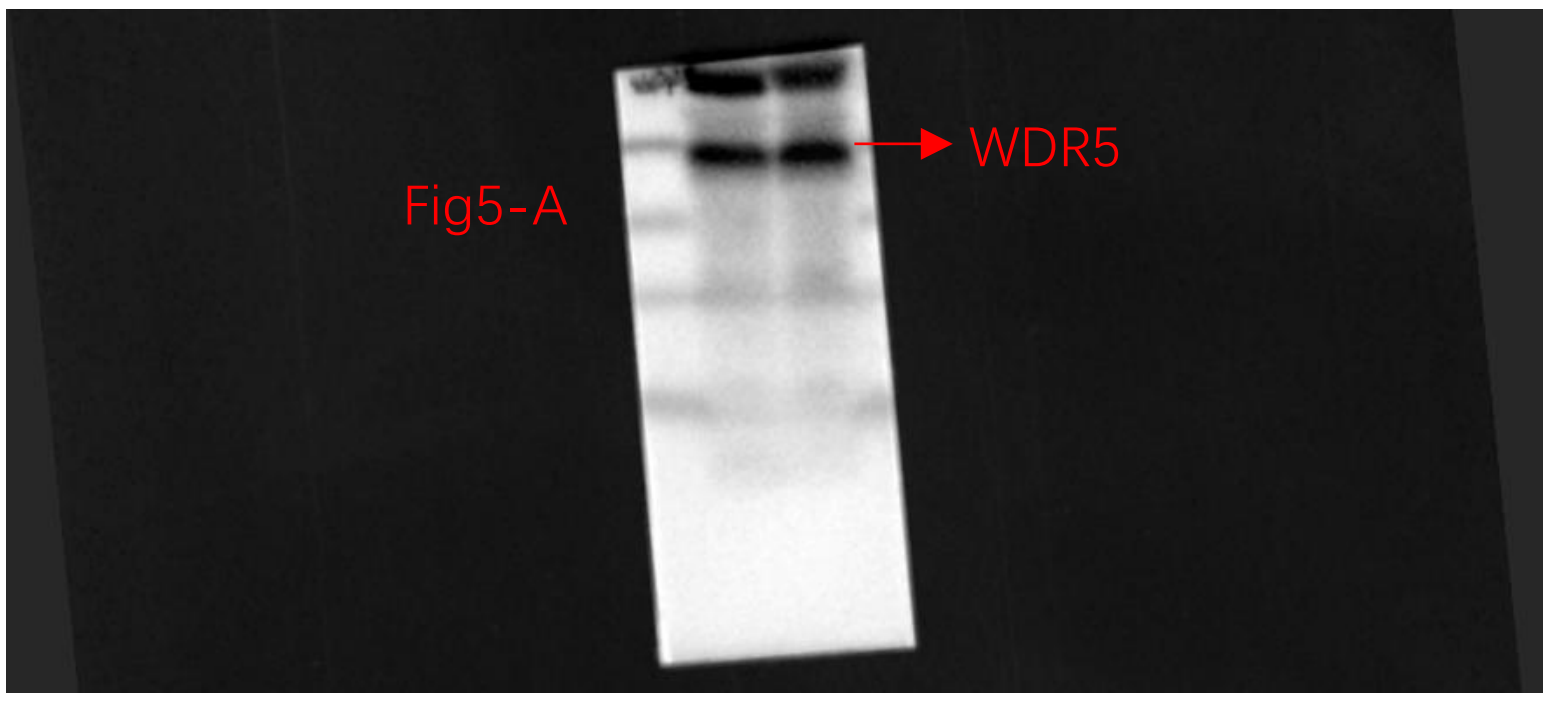
Fig5-A
WDR5

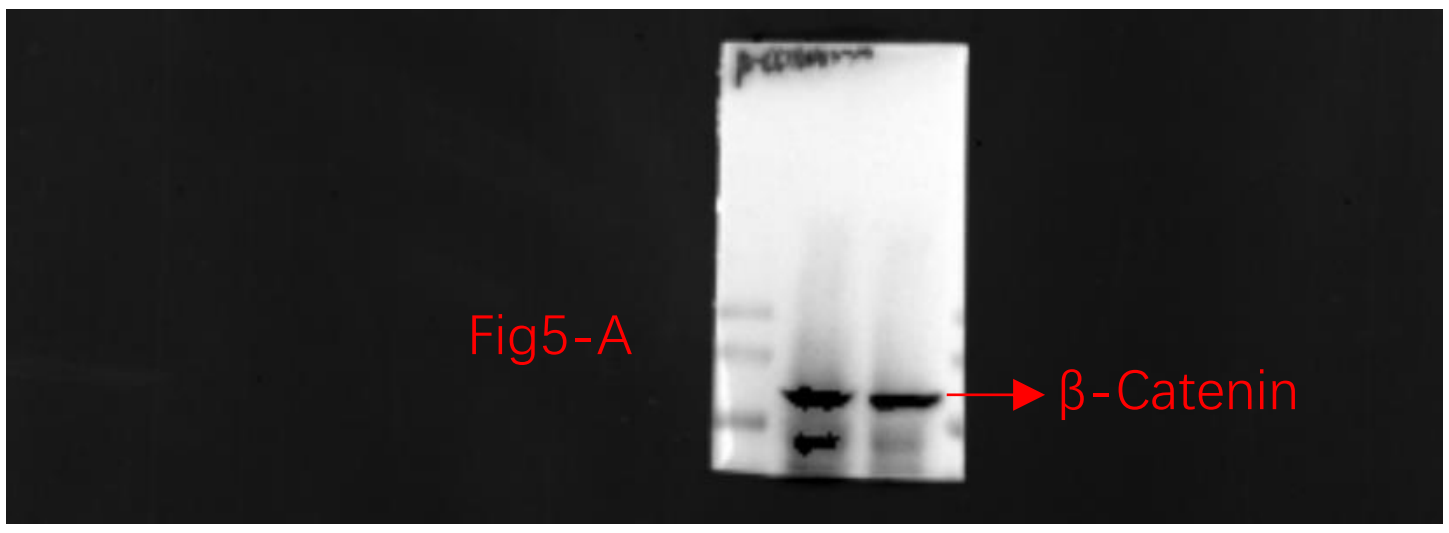
Fig5-A
β-Catenin

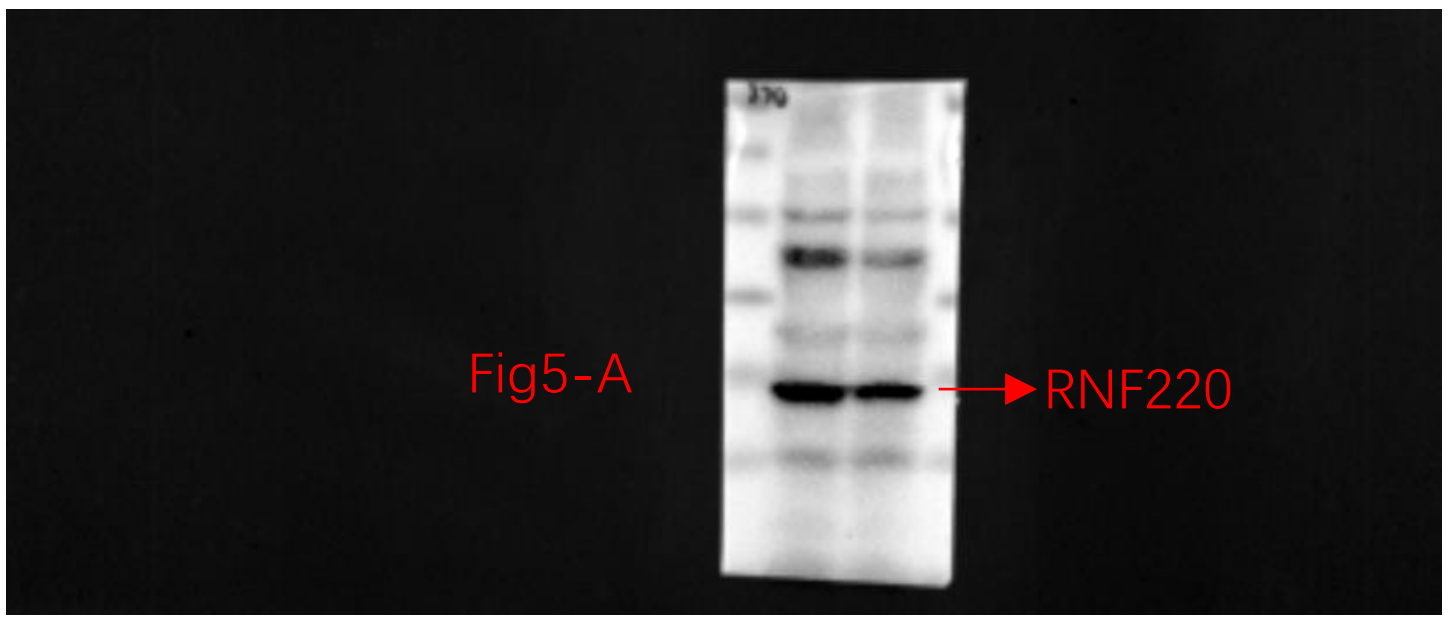
Fig5-A
RNF220

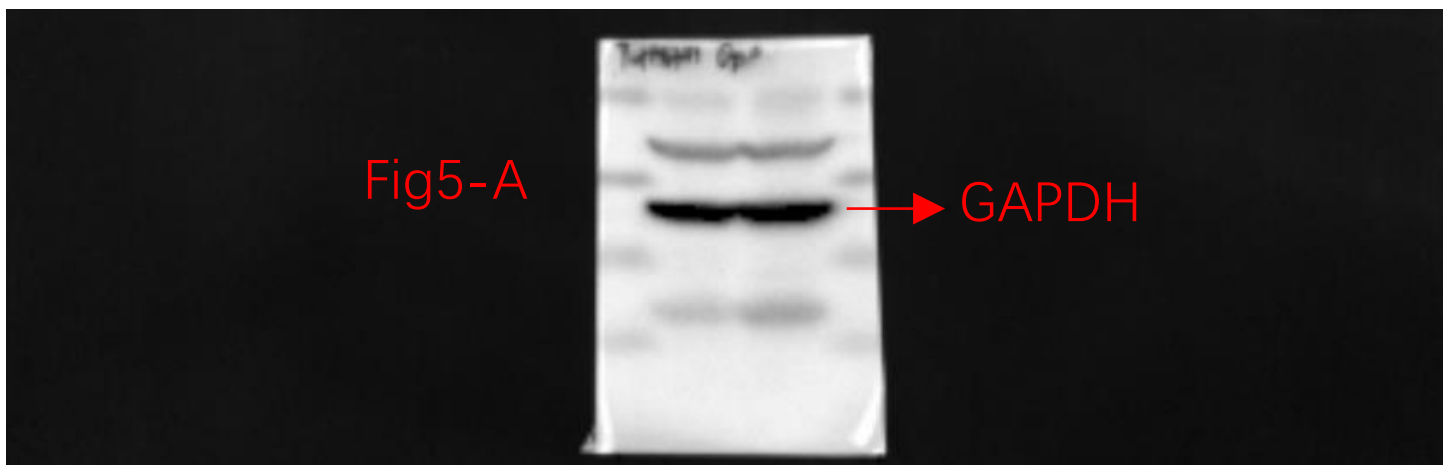
Fig5-A
GAPDH

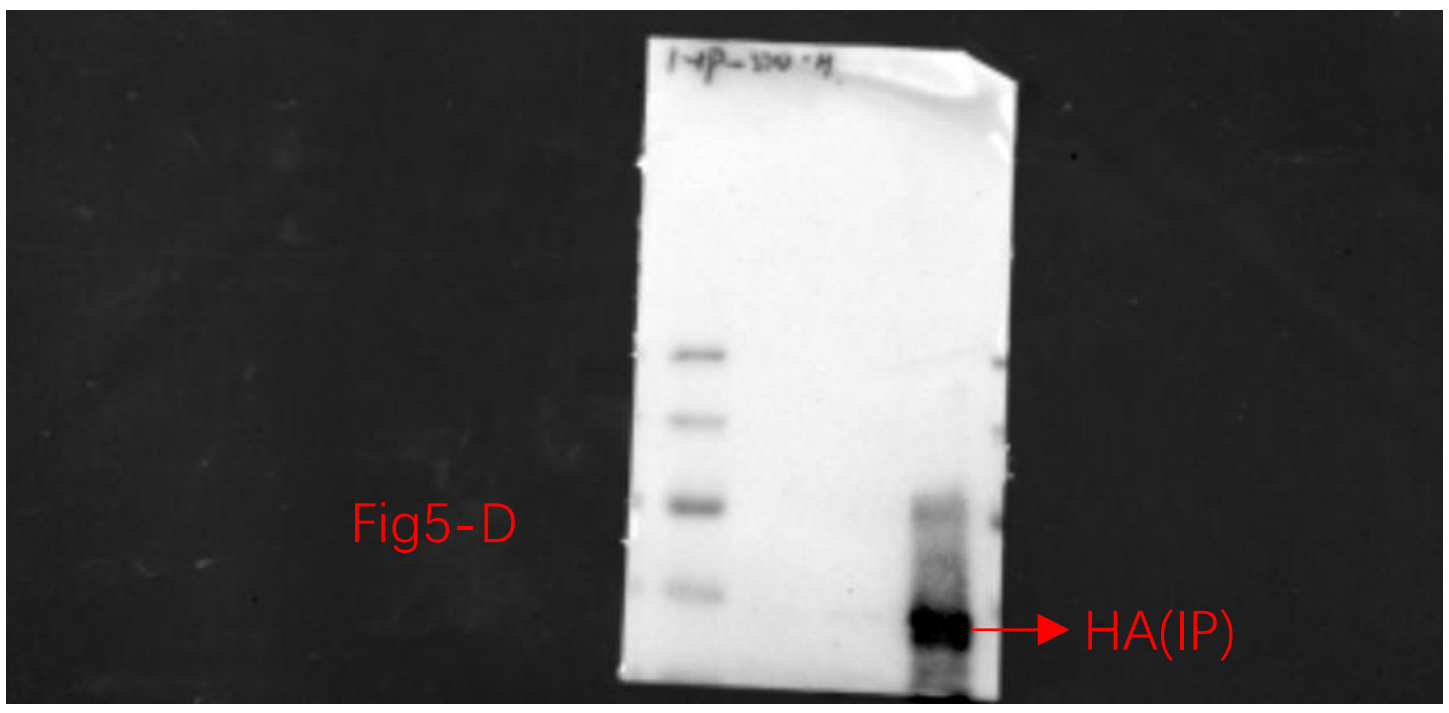
Fig5-D
HA(IP)

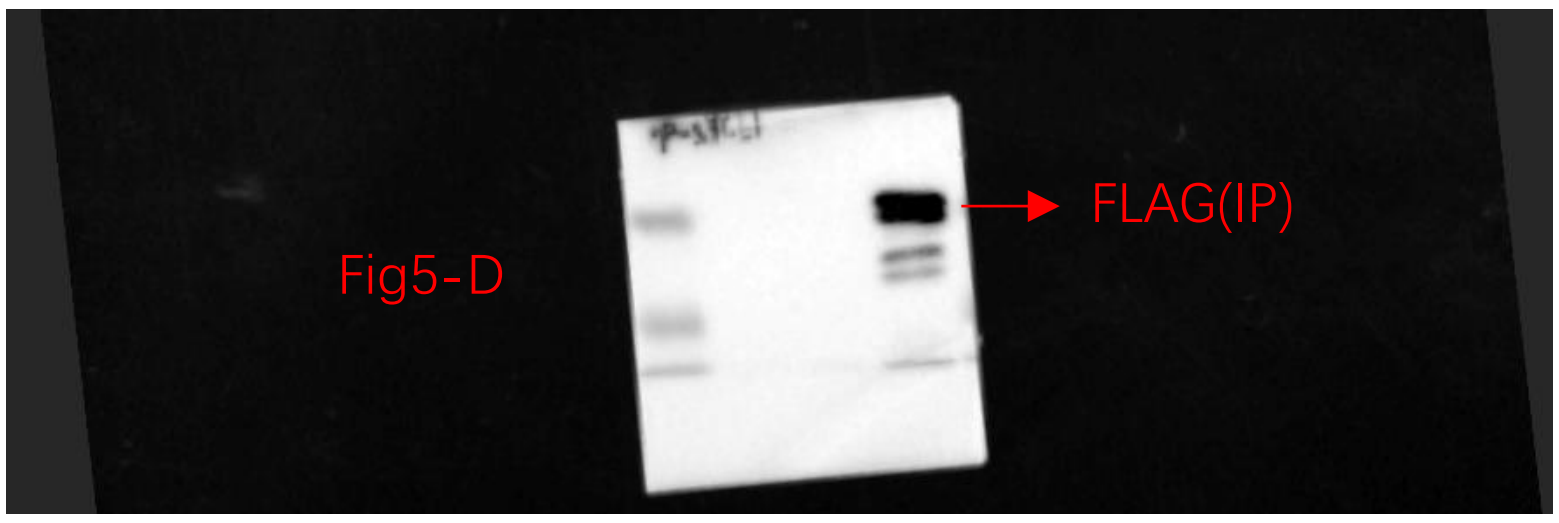
Fig5-D
FLAG(IP)

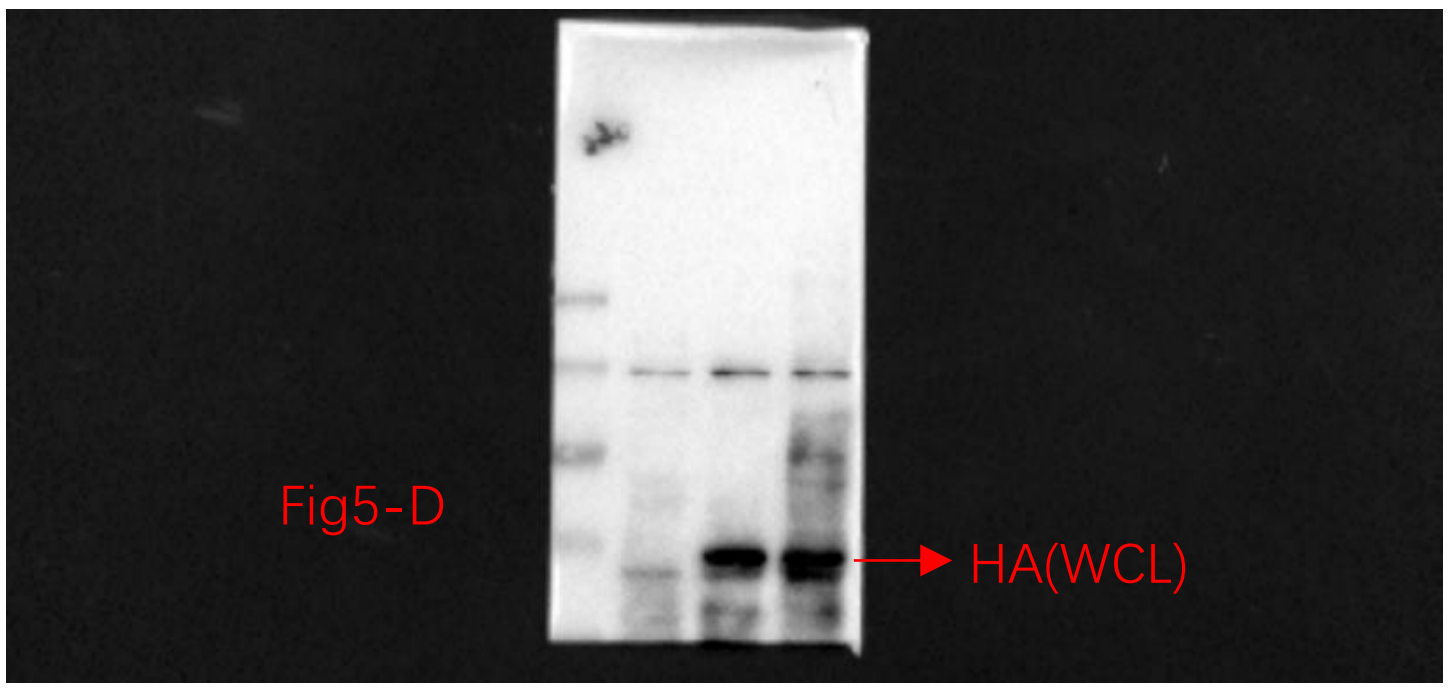
Fig5-D
HA(WCL)

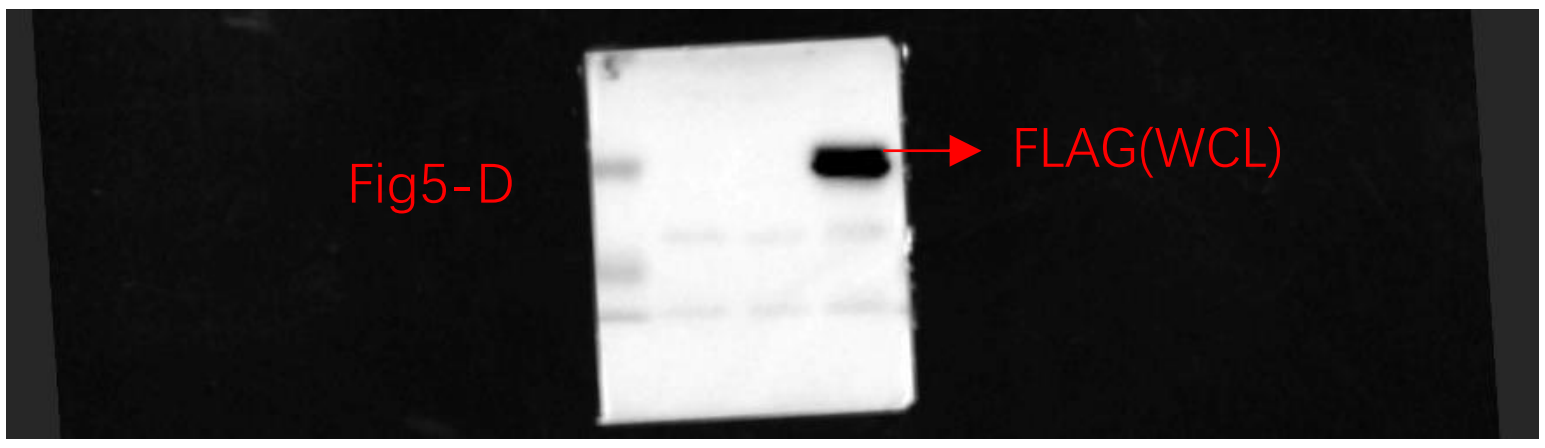
Fig5-D
FLAG(WCL)

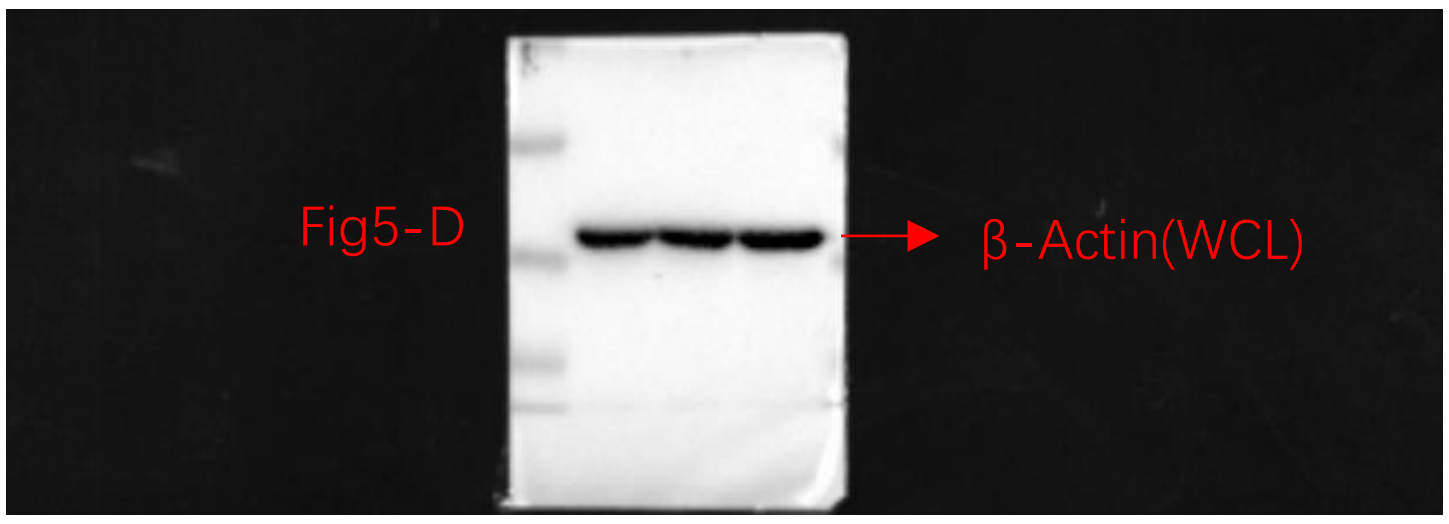
Fig5-D
β-Actin(WCL)

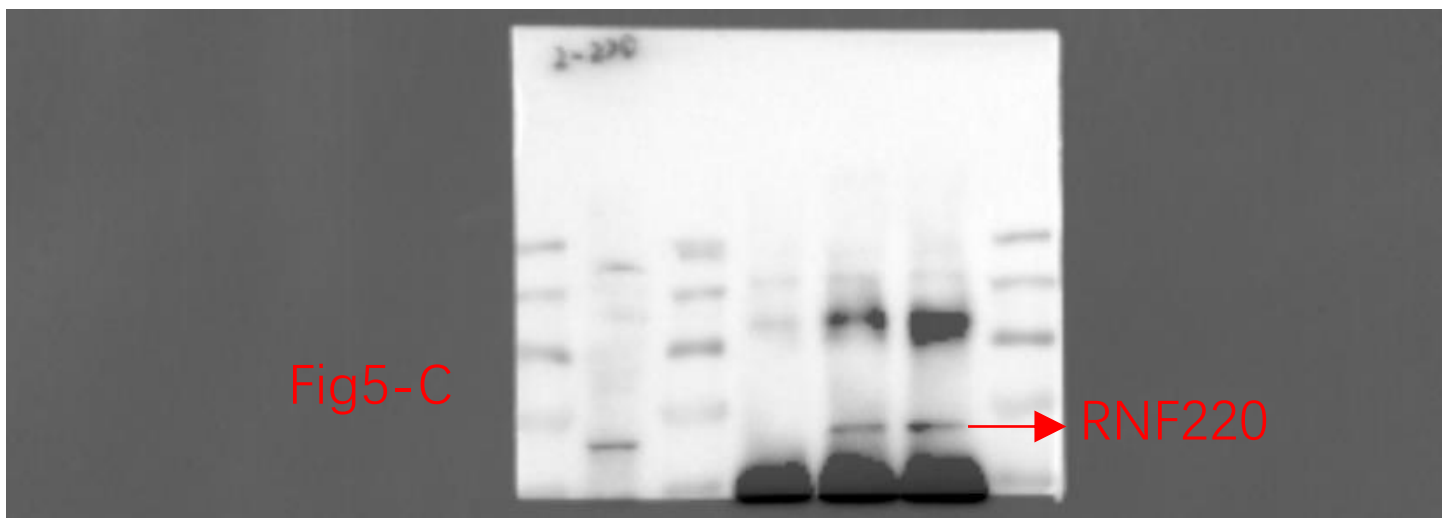
Fig5-C
RNF220

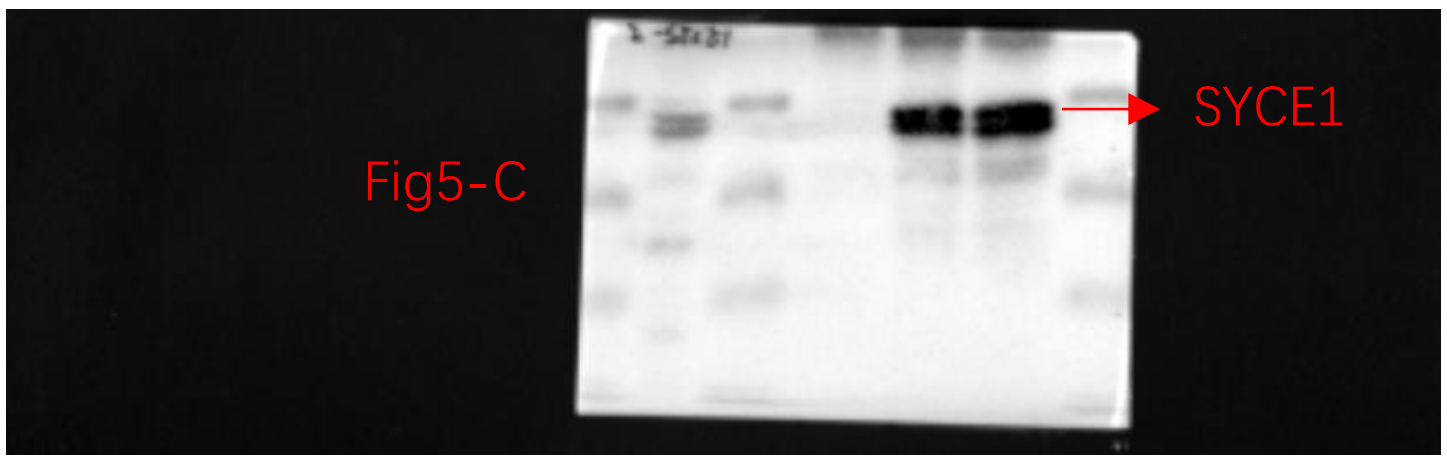
Fig5-C
SYCE1

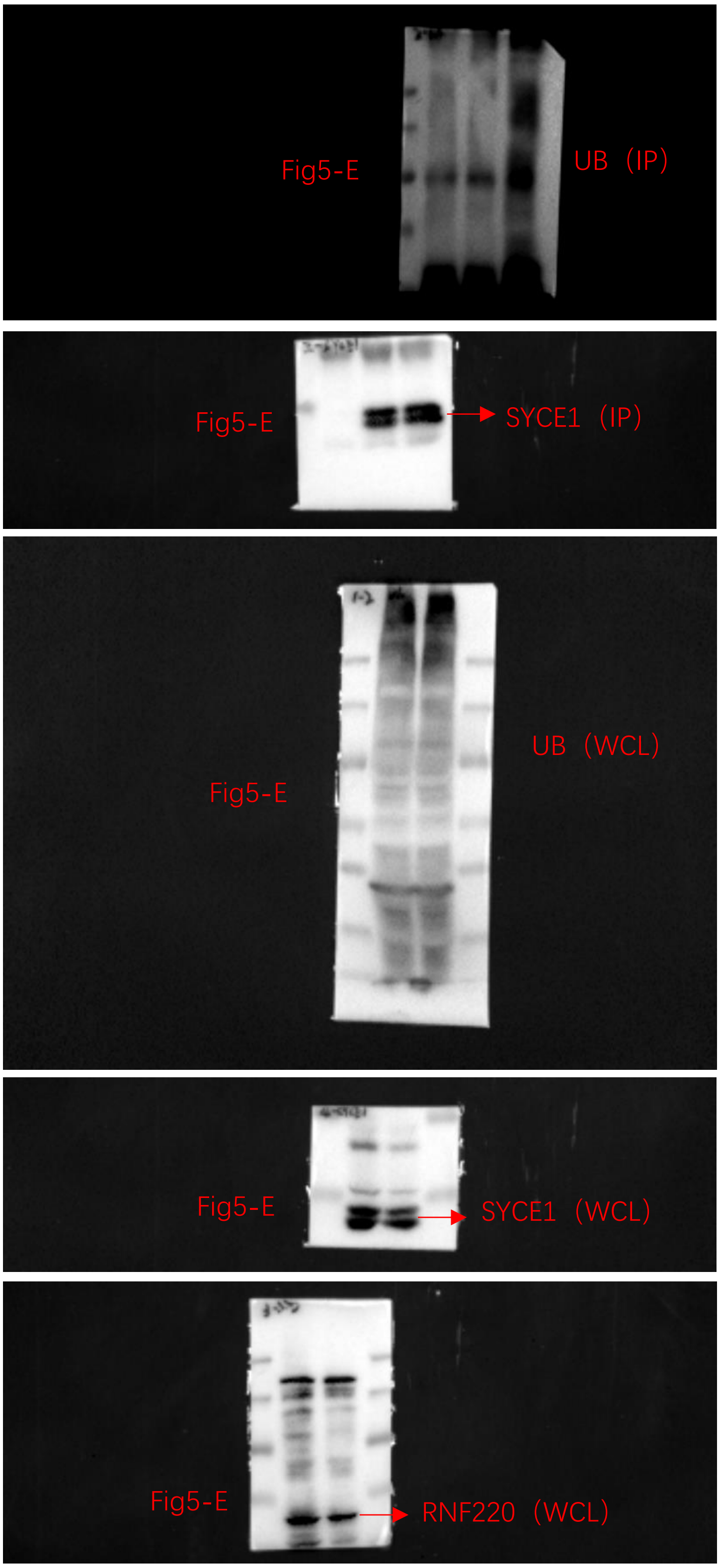
Fig5-E
UB（IP）
Fig5-E
SYCE1（IP）
Fig5-E
UB（WCL）
Fig5-E
SYCE1（WCL）
Fig5-E
RNF220（WCL）

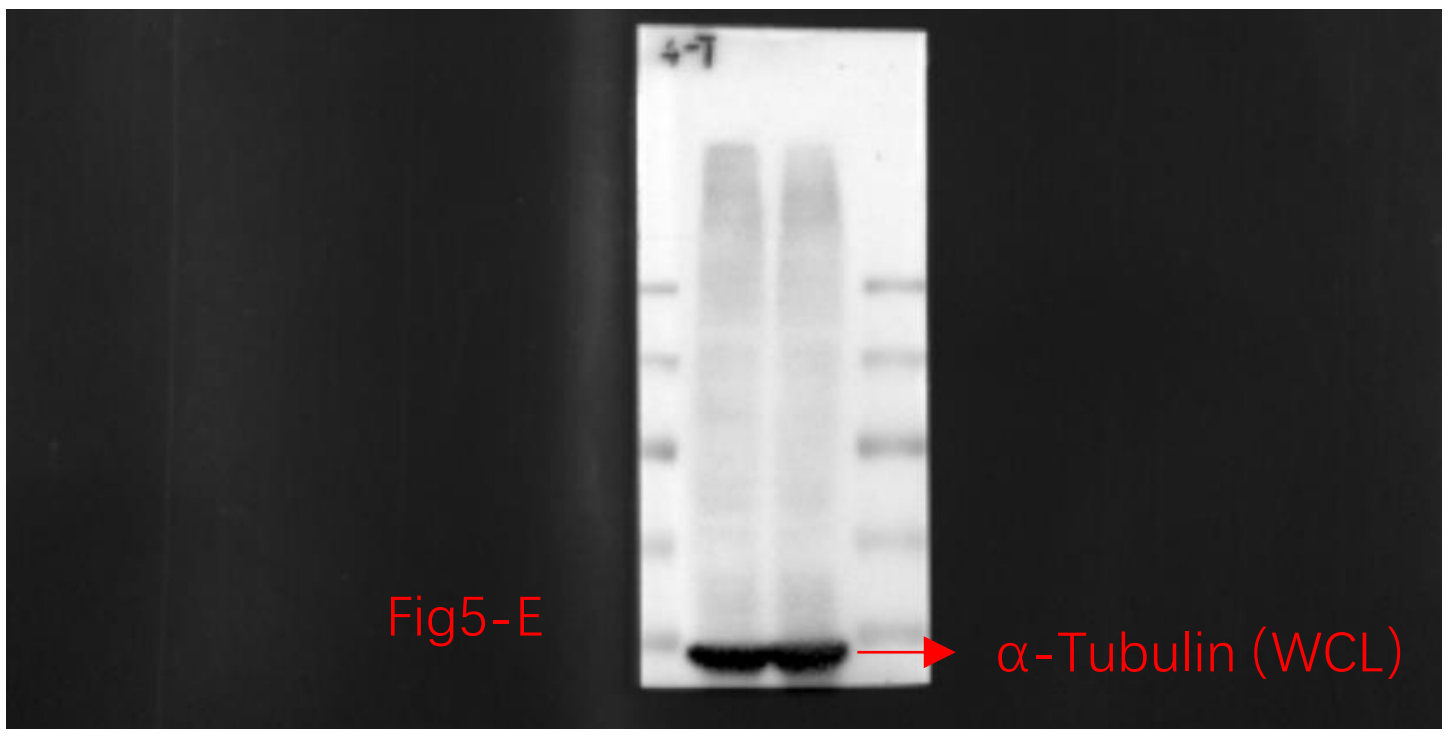
4-T
Fig5-E
α-Tubulin (WCL)

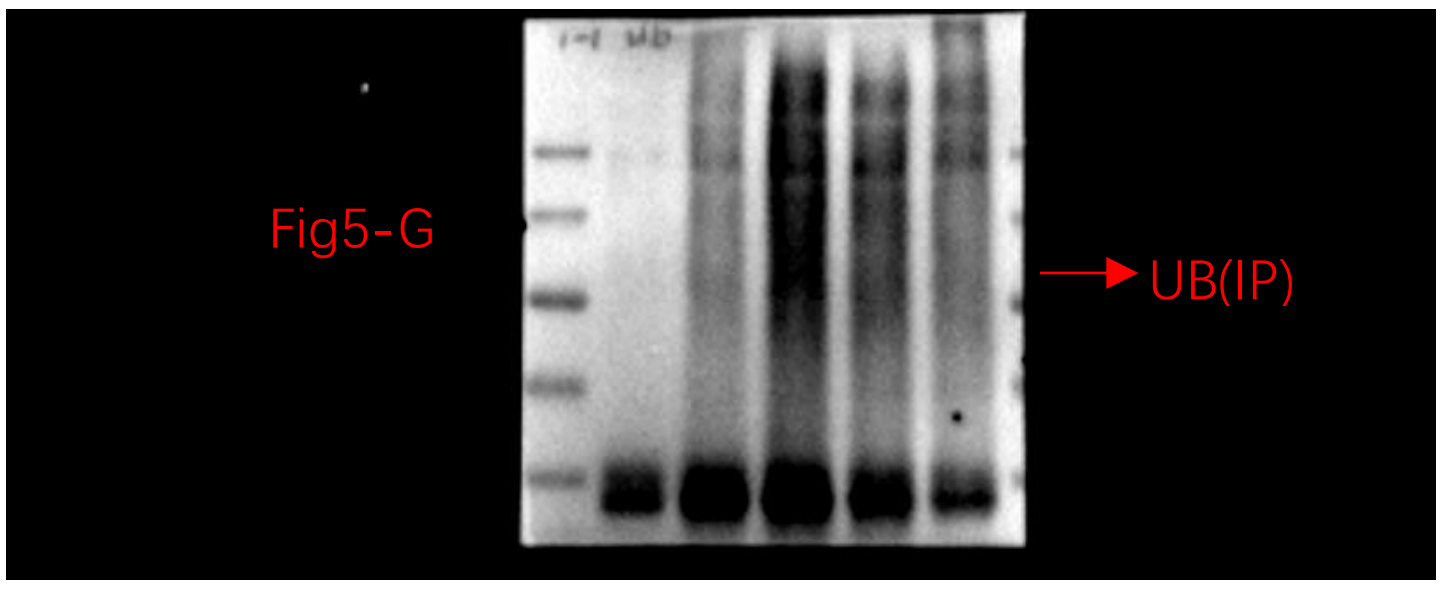
Fig5-G
UB(IP)

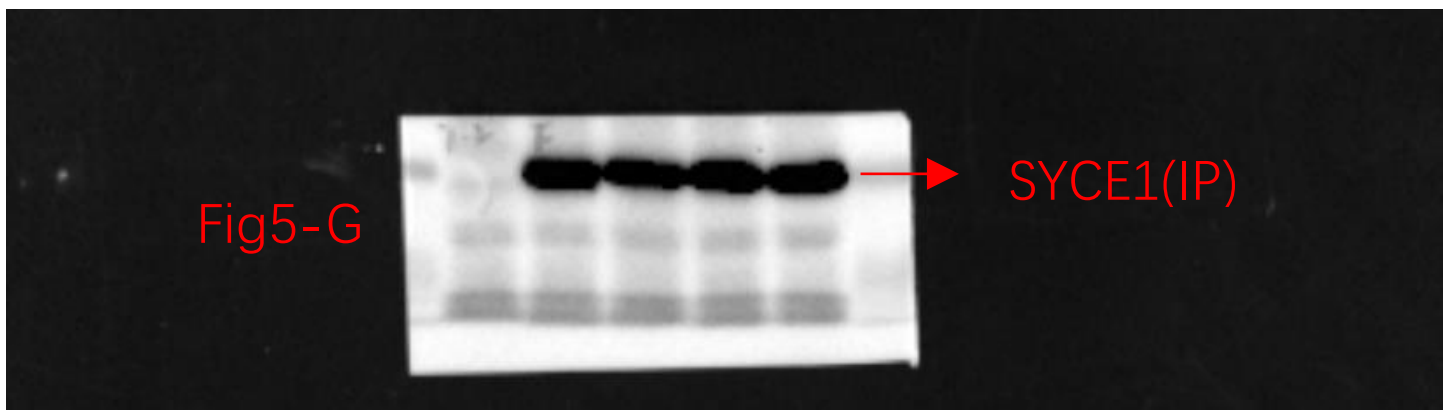
Fig5-G
SYCE1(IP)

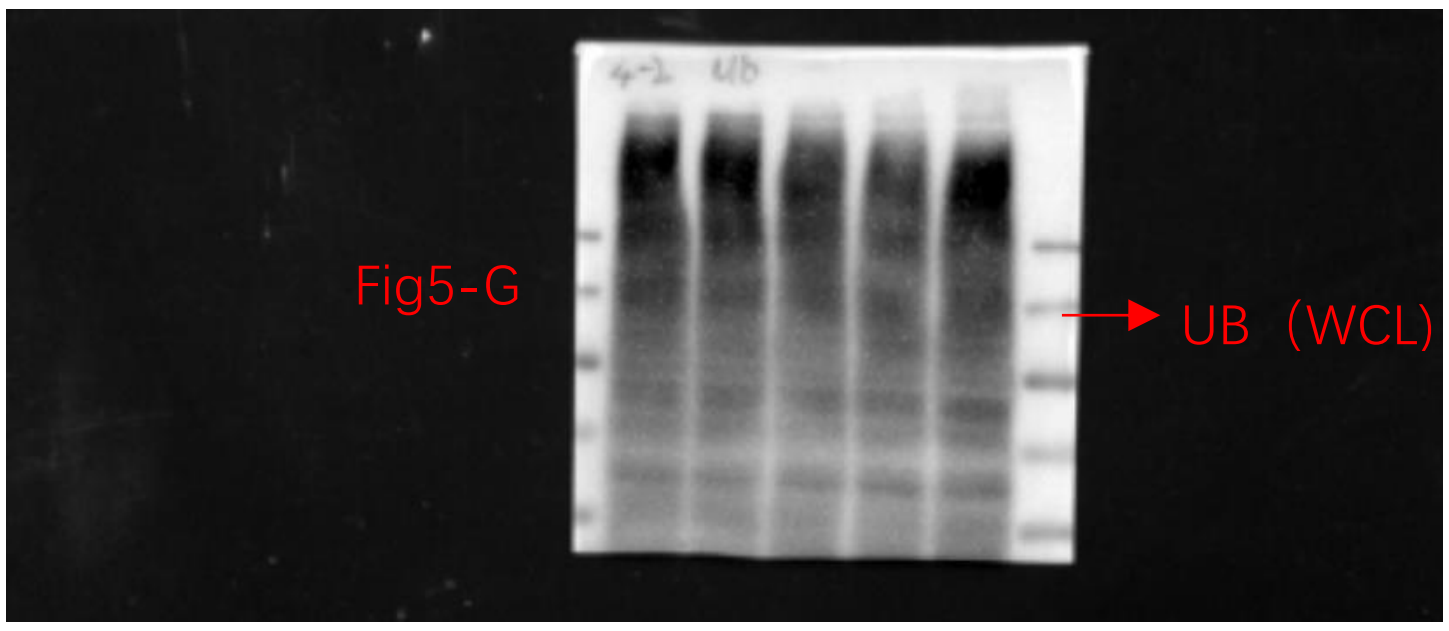
Fig5-G
UB（WCL)

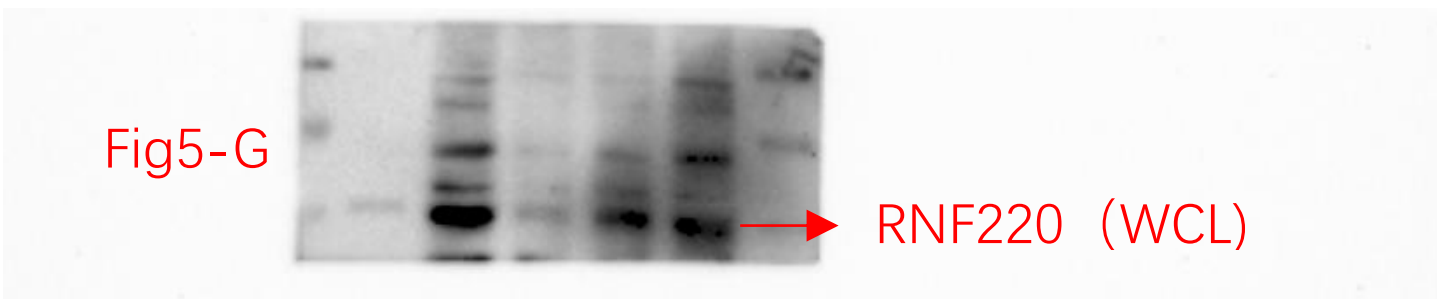
Fig5-G
RNF220（WCL)

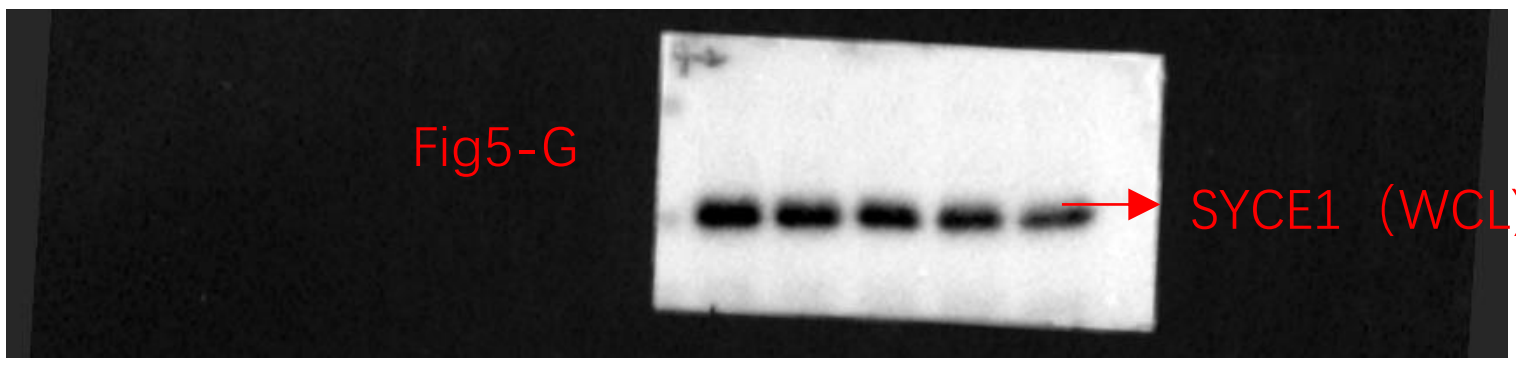
Fig5-G
SYCE1（WCL)

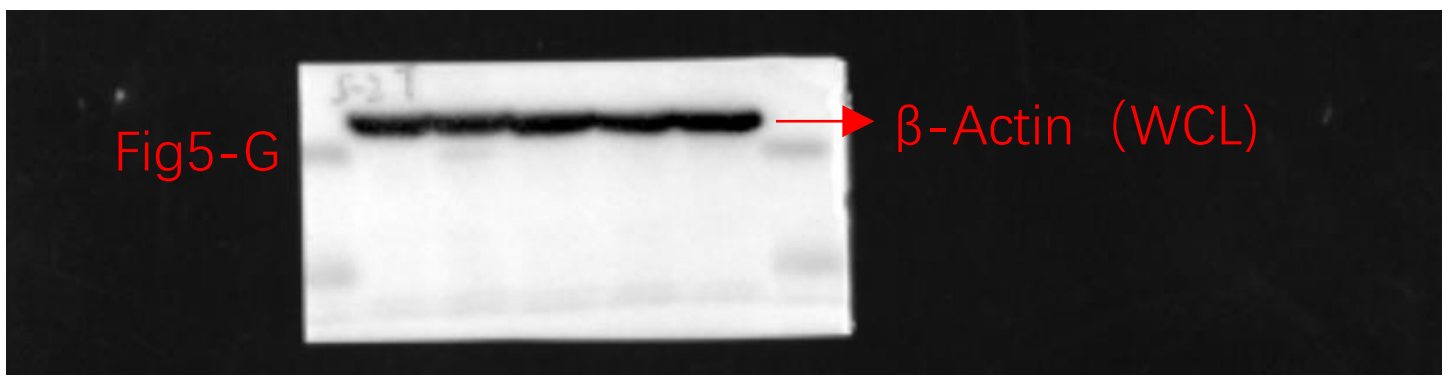
Fig5-G
β-Actin（WCL)

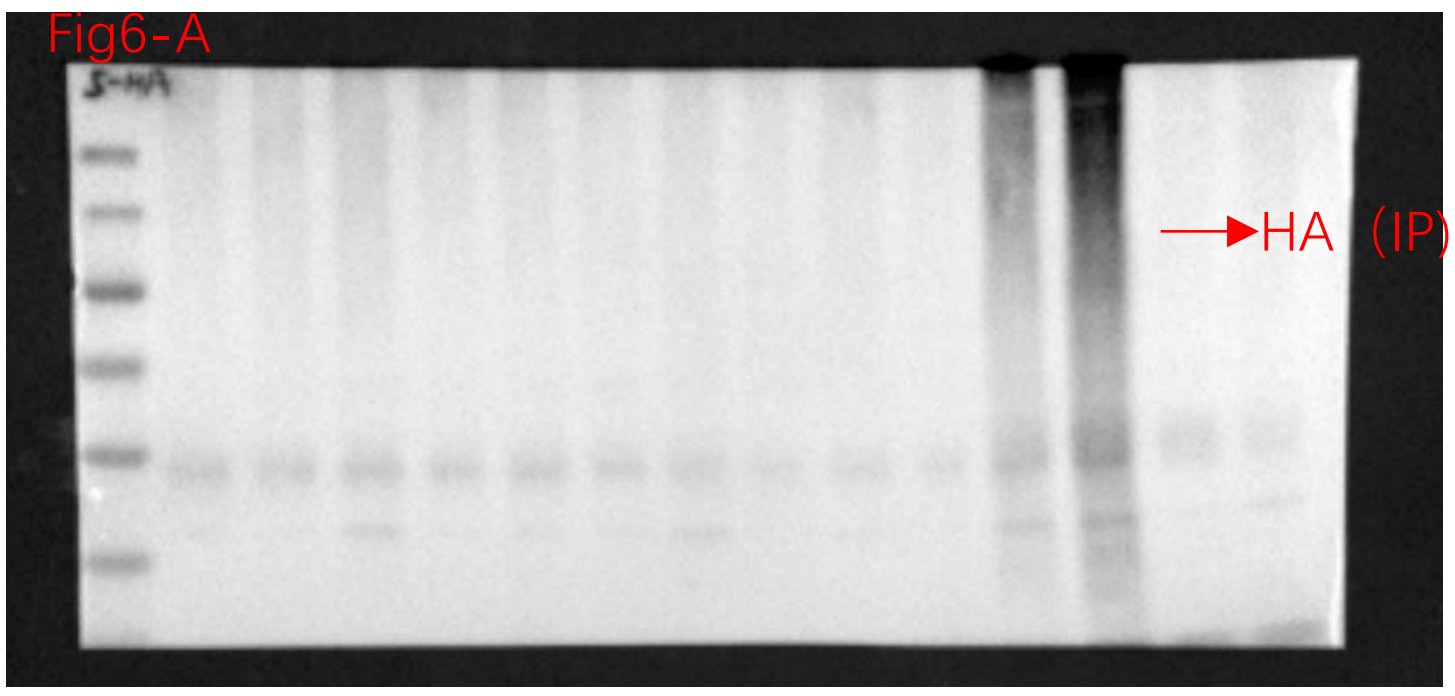
Fig6-A
HA（IP)

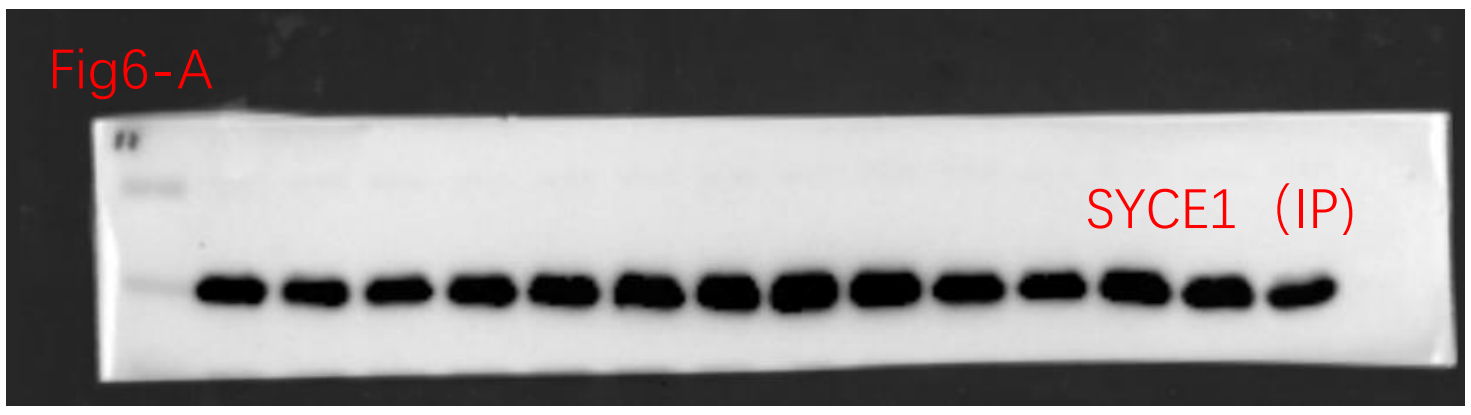
Fig6-A
SYCE1（IP)

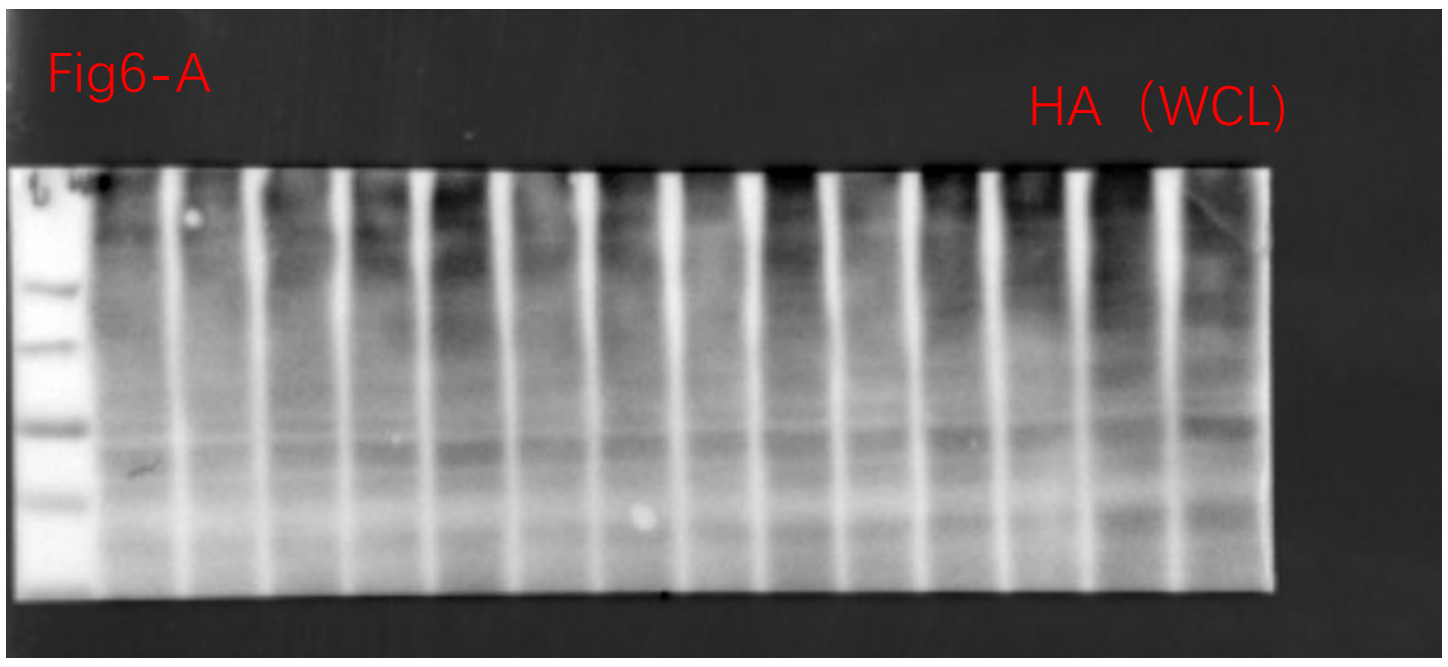
Fig6-A
HA（WCL)

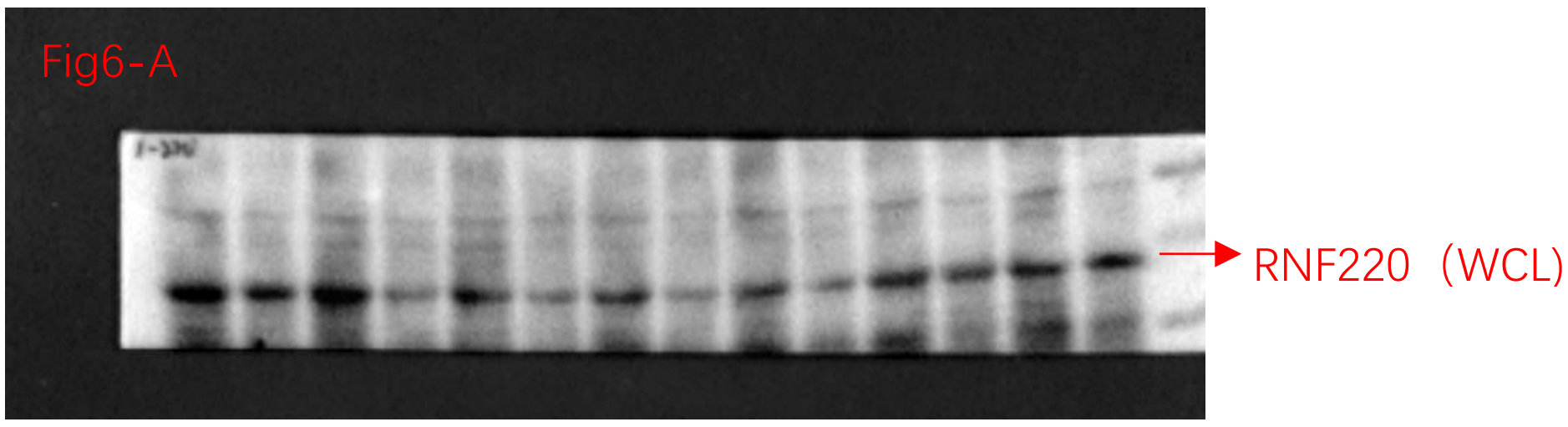
Fig6-A
RNF220（WCL)

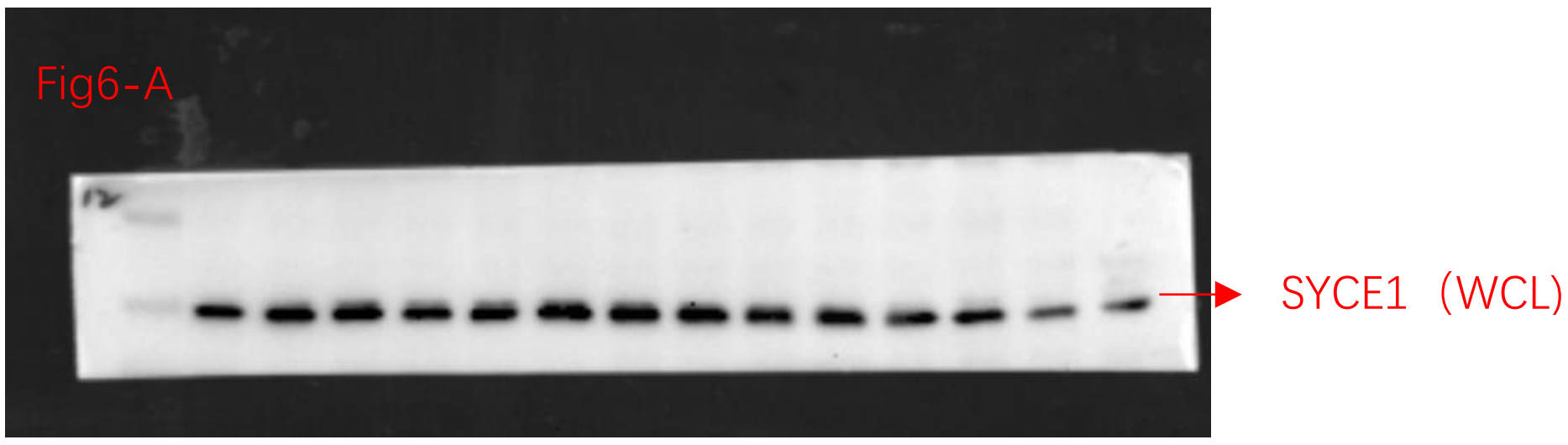
Fig6-A
SYCE1（WCL)

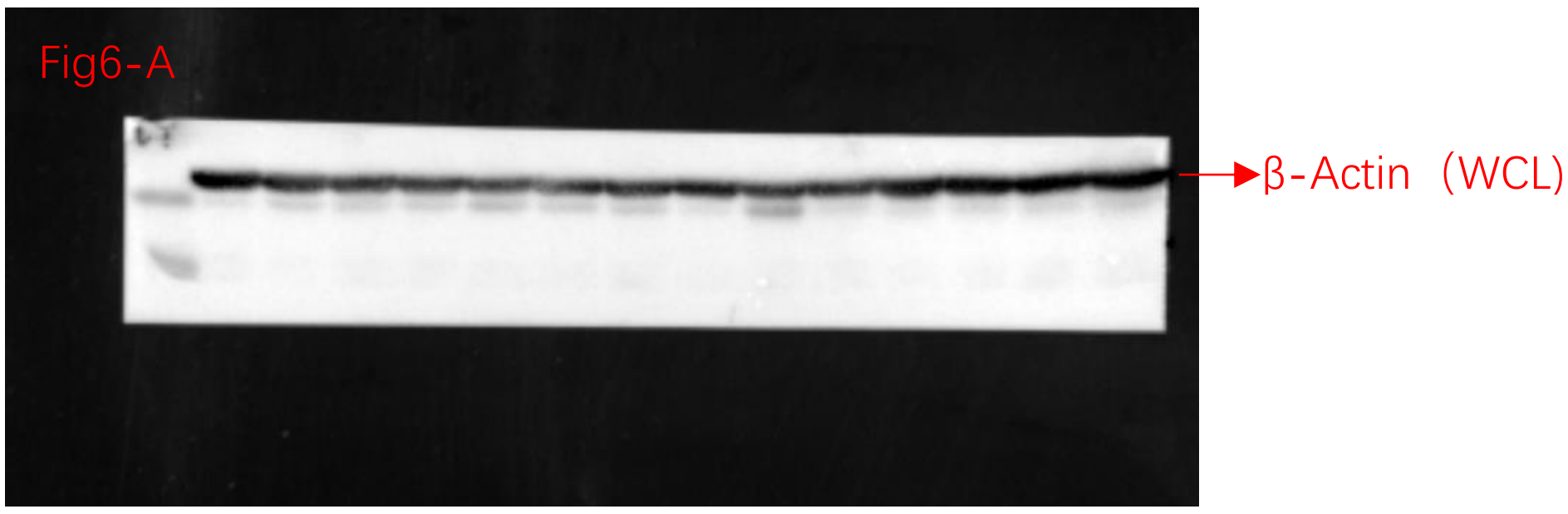
Fig6-A
β-Actin（WCL)

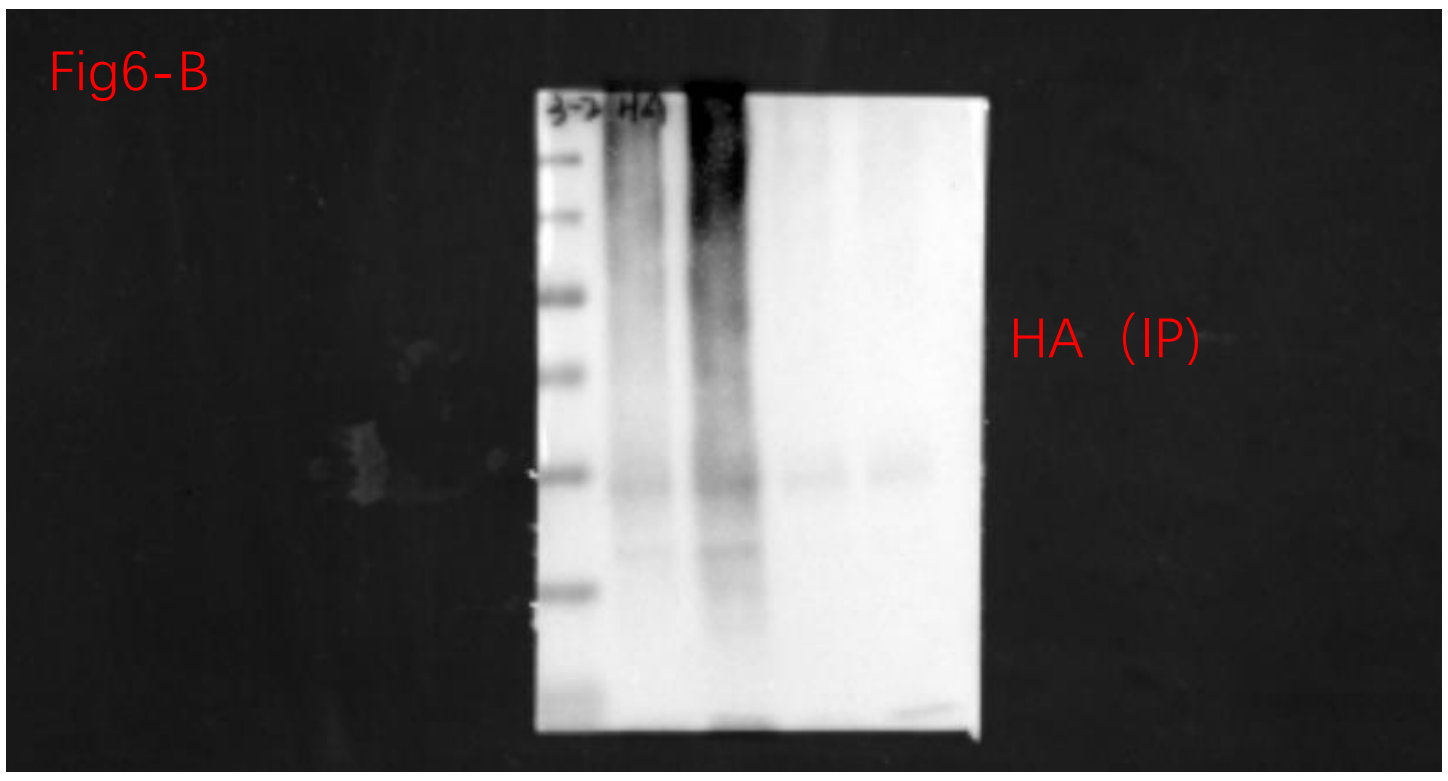
Fig6-B
HA（IP)

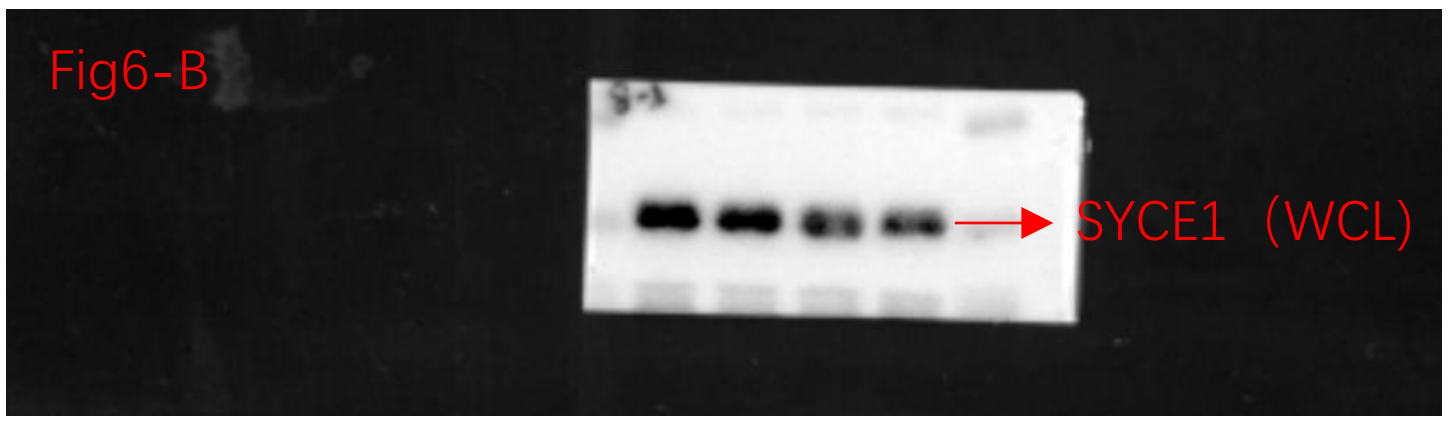
Fig6-B
SYCE1 （WCL)

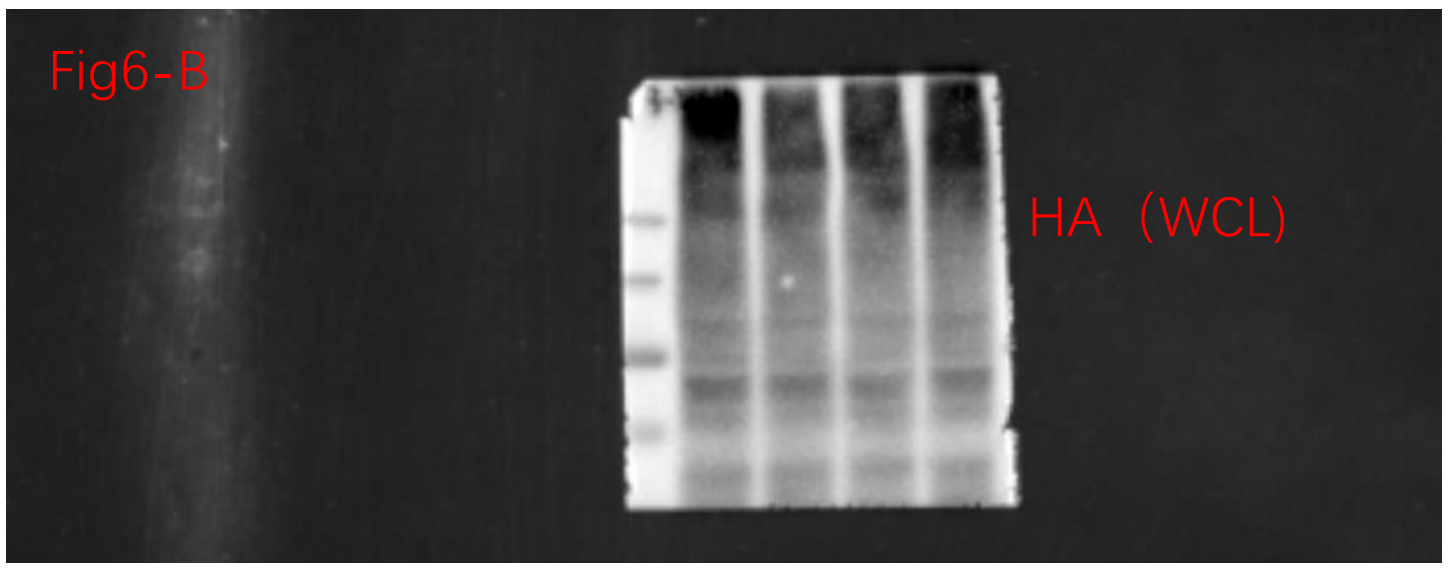
Fig6-B
HA （WCL)

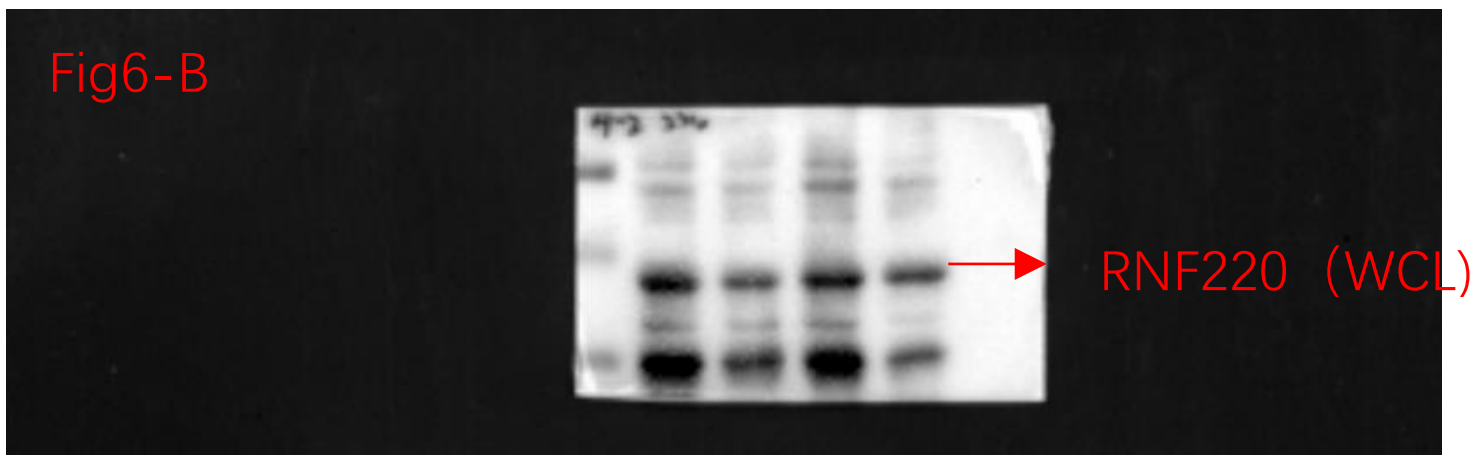
Fig6-B
RNF220 （WCL)

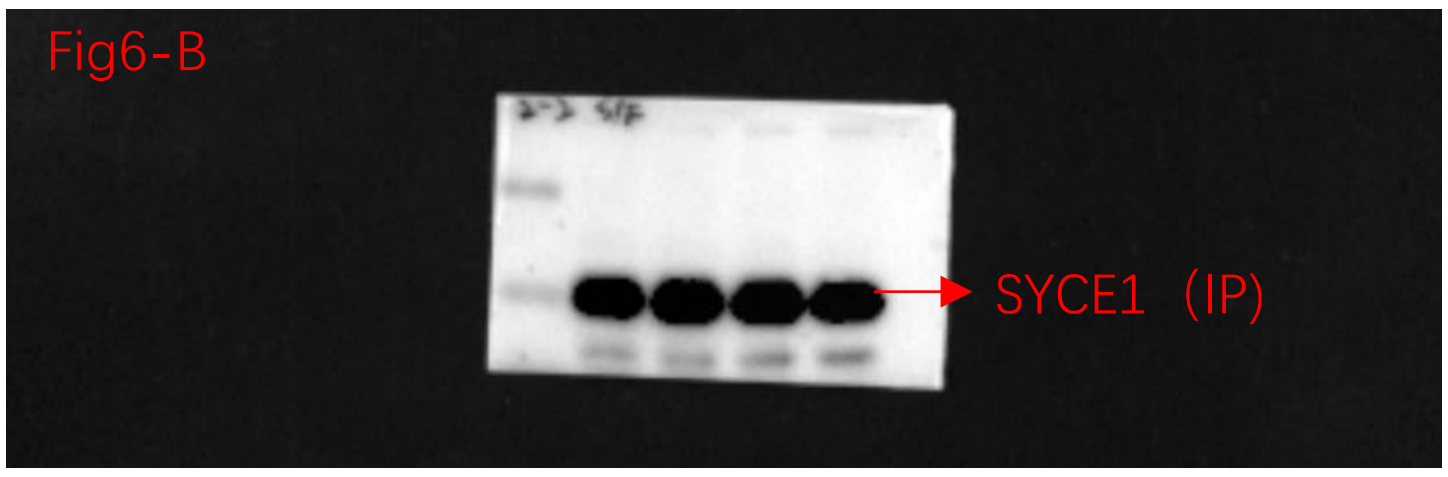
Fig6-B
SYCE1 （IP)

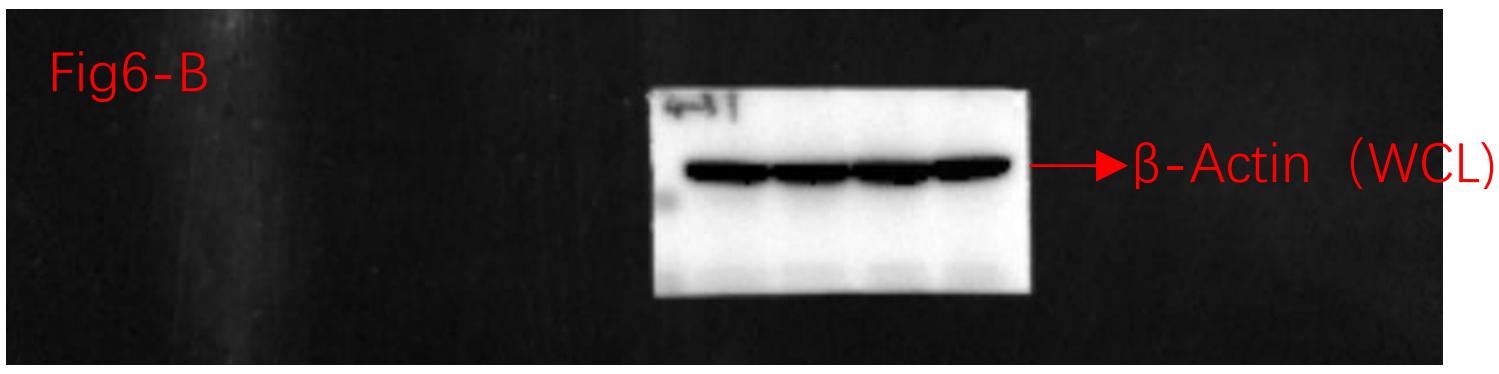
Fig6-B
β-Actin （WCL)

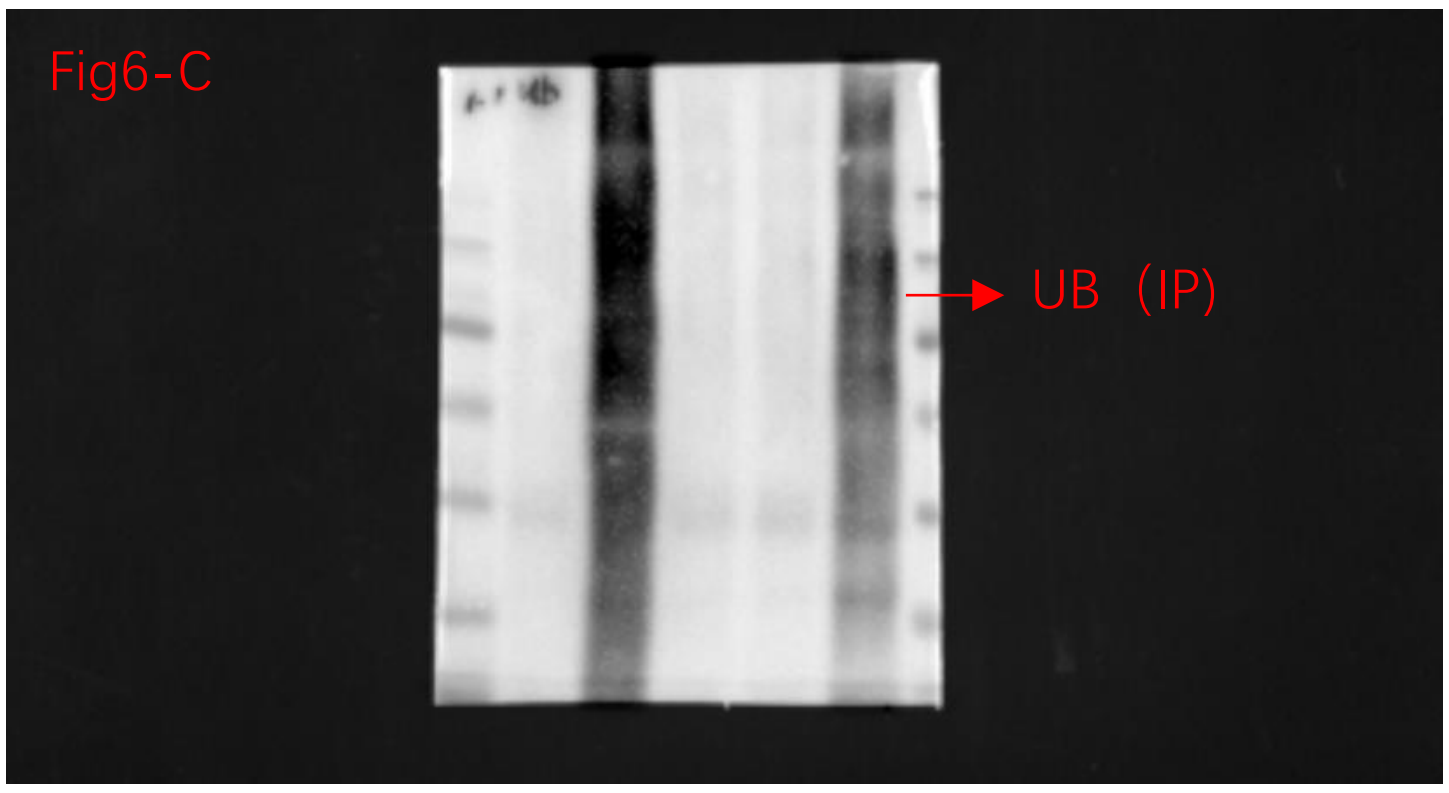
Fig6-C
UB （IP)

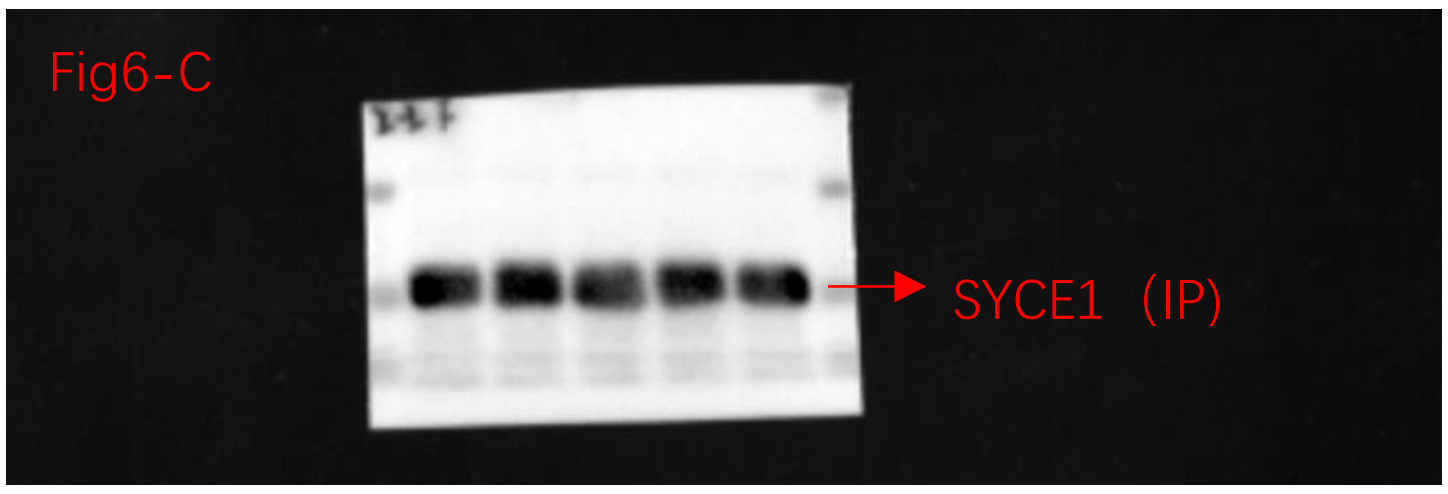
Fig6-C
SYCE1（IP)

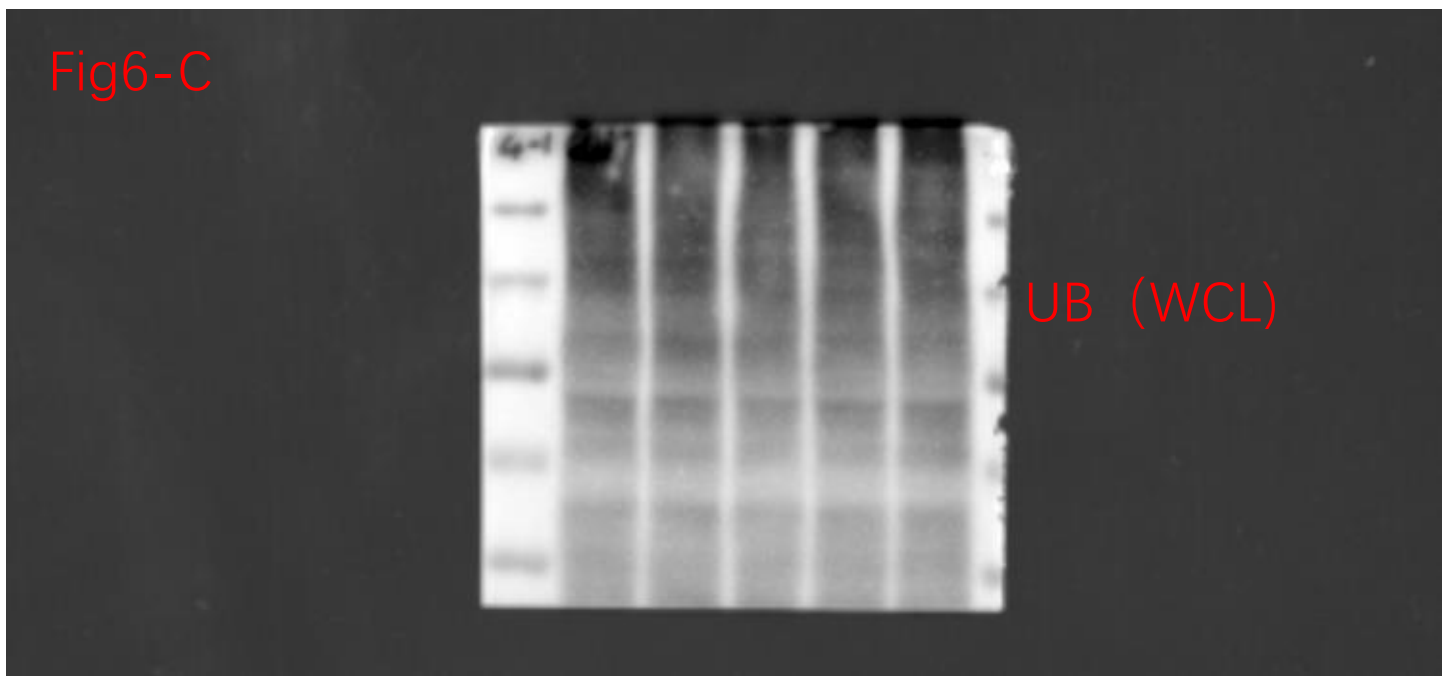
Fig6-C
UB（WCL)

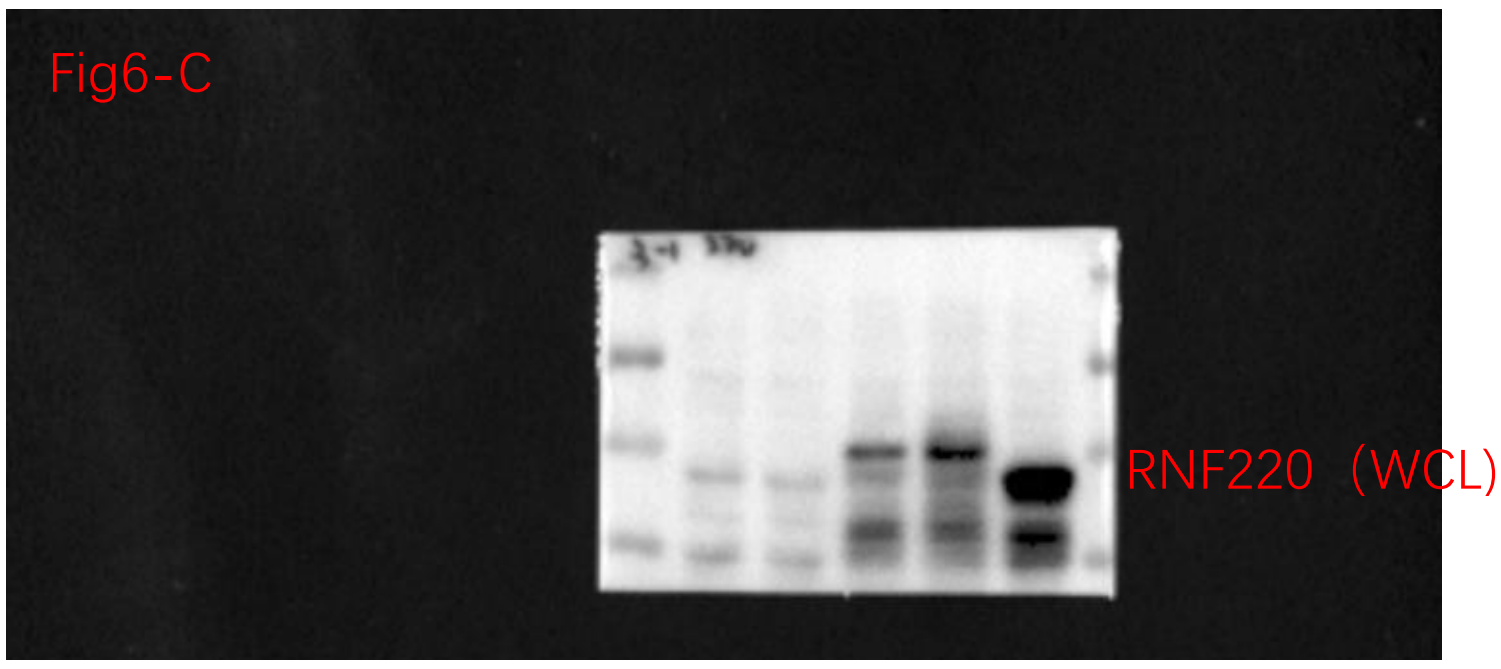
Fig6-C
RNF220（WCL)

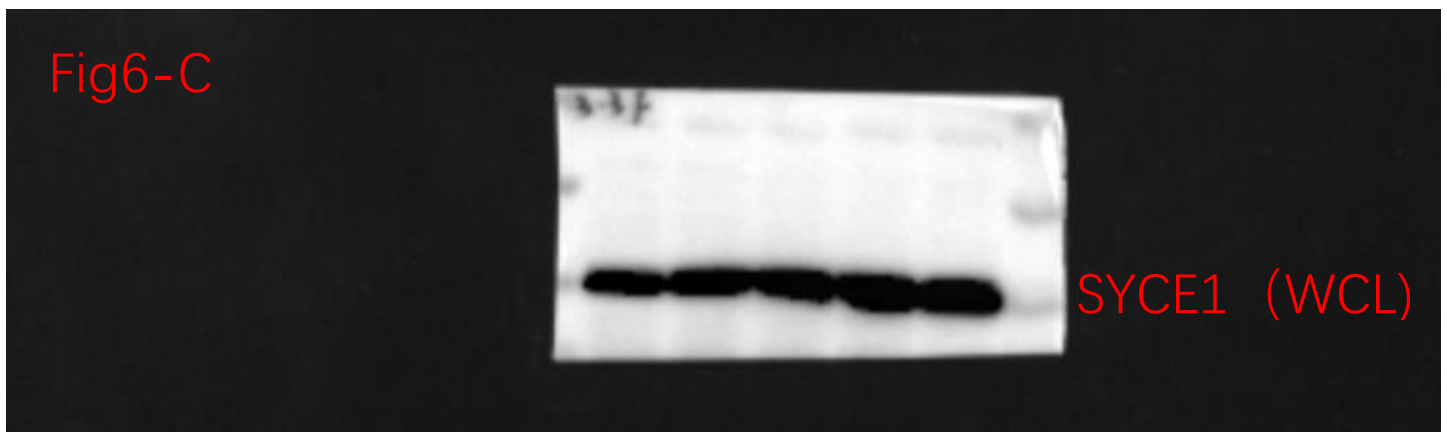
Fig6-C
SYCE1（WCL)

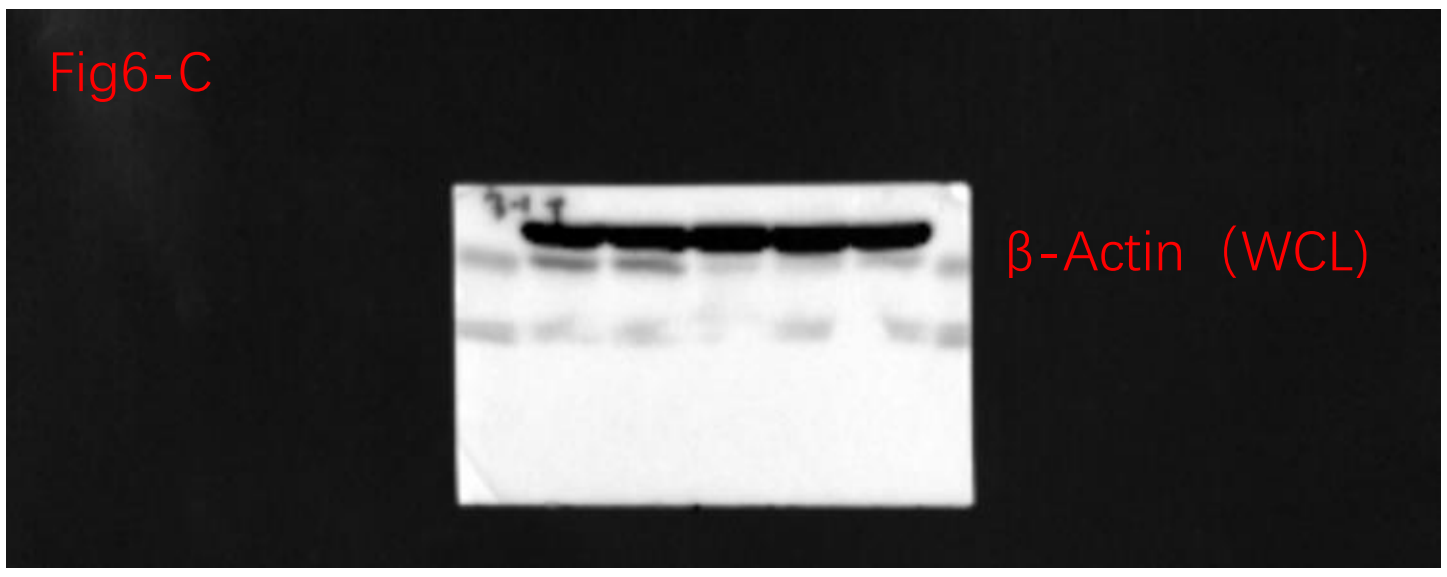
Fig6-C
β-Actin（WCL)
