# Supplementary material for: RNF220 safeguards meiotic synapsis and male fertility by antagonizing SYCE1 ubiquitination
Source: Cell Death Dis. 2026 Jul 24;17(1):895. doi: 10.1038/s41419-026-09128-8 (PMC13683351; doi:10.1038/s41419-026-09128-8)
Supplement: Supplementary file 2 [file 41419_2026_9128_MOESM1_ESM.docx]

**Supplementary Materials**

**RNF220 safeguards meiotic synapsis and male fertility by antagonizing SYCE1 ubiquitination**

Jingmei Cha¹^,^³, Huijuan Lin¹, Yingqian Xia¹, Jianlin Xu¹^,^³, Pengcheng Ma¹^,^⁴^,⁎^, Shuhua Zhao²^,⁎^, Bingyu Mao¹^,^⁴^,⁎^

Supplementary Figure S1. Flow cytometric sorting and purity validation of testicular spermatogenic cells.

Supplementary Figure S2. Generation and fertility phenotype analysis of RNF220 cKO mice.

Supplementary Figure S3. Effects of RNF220 cKO on Sertoli cells, spermatogonial stem cells, and other meiotic stage spermatocytes in the testis.

Supplementary Figure S4 . Validation of TUNEL assay specificity.

Supplementary Figure S5. Isolated wild-type mouse epididymal sperm.

Table S1 Antibodies used in this study.

**Supplementary Figure S1. Flow cytometric sorting and purity validation of testicular spermatogenic cells**

(A) Flow cytometric analysis of spermatogenic cells from mouse testes, stained with Hoechst 33342 for DNA content. The gating strategy identifies three distinct populations: haploid (N, spermatids), diploid (2N, spermatogonia / Somatic cells), and tetraploid (4N, spermatocytes).

(B) Purity validation of sorted haploid spermatids. Left: Immunofluorescence staining with the acrosomal marker PNA (red) and DAPI (blue) confirms the identity of sorted cells. Scale bar: 10 μm. Right: Quantification of PNA-positive spermatids in the sorted population (*N*=701 cells counted).

(C) Purity validation of sorted tetraploid spermatocytes. Left: Immunofluorescence staining with the synaptonemal complex lateral element marker SYCP3 (green) and DAPI (blue) confirms the identity of sorted cells. Scale bar: 2 μm. Right: Quantification of SYCP3-positive spermatocytes in the sorted population (*N*=108 cells counted).

**Supplementary Figure S2. Generation and fertility phenotype analysis of RNF220 cKO mice**

(A) Schematic diagram of the breeding strategy for generating RNF220 cKO mice. LoxP sites flank exon 2 of the *RNF220* gene.

(B) Genotyping validation of RNF220 cKO mice. Genomic DNA was amplified using primer pairs F1/R1 (detecting floxed [600 bp] and wild-type [500 bp] alleles), F1/R3 (detecting the knockout [KO] allele, 200 bp), and Stra8-Cre (detecting the Cre transgene, 400 bp).

(C) Fertility assessment of wild-type (WT) and RNF220 cKO male mice. 13 males (8 weeks old) per group were individually mated with WT females at a 1:1 ratio. The total number of litters, total number of pups born, and average litter size were recorded over a 5-month period.

**Supplementary Figure S3. Effects of RNF220 cKO on Sertoli cells, spermatogonial stem cells, and other meiotic stage spermatocytes in the testis.**

(A) Representative immunofluorescence images of Sertoli cells in testes from wild-type (WT) and RNF220 cKO mice. Triple staining: PNA (red, acrosome), WT1 (green, Sertoli cells), and DAPI (blue, nuclei). Scale bar: 20 µm.

(B) Quantitative analysis of WT1-positive Sertoli cells at different stages of the spermatogenic cycle (*n*=3 mice; *N*, counted seminiferous tubules). ns, not significant.

(C) Representative immunofluorescence images of spermatogonial stem cells in testes from WT and RNF220 cKO mice. Triple staining: PNA (red, acrosome), PLZF (green, spermatogonial stem cells), and DAPI (blue, nuclei). Scale bar: 20 µm.

(D) Quantitative analysis of PLZF-positive spermatogonial stem cells at different stages of the spermatogenic cycle (*n*=3 mice; *N*, counted seminiferous tubules). ns, not significant. **P* < 0.05.

(E) Homologous chromosome synapsis in leptotene (lepSC), zygotene (zygSC), and diplotene (dipSC) spermatocytes from WT and RNF220 cKO mice detected by spermatocyte nuclear spread assay. SYCP3 (green, lateral elements of the synaptonemal complex); SYCP1 (red, transverse filaments). Scale bar: 5 μm.

(F) DNA double-strand break (DSB) formation and repair in leptotene (lepSC), zygotene (zygSC), and diplotene (dipSC) spermatocytes from WT and RNF220 cKO mice detected by spermatocyte nuclear spread assay. SYCP3 (green, lateral elements of the synaptonemal complex); γH2AX (red, DNA damage sites). Scale bar: 5 μm.

**Supplementary Figure S4 . Validation of TUNEL assay specificity.**

Representative images of TUNEL staining (green) in mouse testis sections. Positive control sections were pretreated with DNase I to induce DNA breaks, showing robust TUNEL signals. Negative control sections were processed without terminal deoxynucleotidyl transferase (TdT) enzyme, showing no specific TUNEL staining. PNA (red) stains the acrosome of spermatids, and DAPI (blue) stains nuclei. Scale bar: 20 μm.

**Supplementary Figure S5. Isolated wild-type mouse epididymal sperm.**

Representative bright-field micrograph of purified epididymal sperm; white circles indicate sporadic somatic cells and cellular debris. Scale bar: 200 μm.

Table S1 Antibodies used in this study.

| Antibody | Vendor | Catalog No. | Working Dilution |
| --- | --- | --- | --- |
| Anti-RNF220 | Sigma | HPA027578 | WB:1:1000  IF:1:200 |
| Anti-DDX4 | Abcam | ab27591 | IF:1:200 |
| Anti-WT1 | Abcam | ab89901 | IF:1:50 |
| Anti-PLZF | Santa Cruz | sc-28319 | IF:1:50 |
| Anti-SYCP1 | Abcam | ab175191 | WB:1:1000  IF:1:200 |
| Anti-SYCP3 | Abcam | ab97672 | WB:1:1000  IF:1:800 |
| Anti-γH2AX | CST | 9718 | IF:1:100 |
| Anti-SYCE1 | Proteintech | 11063-1-AP | WB:1:1000  IP: 1:200  IF:1:100 |
| Anti-H2A | CST | 12349 | WB:1:1000 |
| Anti-H2B | CST | 12364 | WB:1:1000 |
| Anti-H3 | CST | 9715 | WB:1:1000 |
| Anti-H4 | CST | 13919 | WB:1:1000 |
| Anti-PRM1 | Proteintech | 15697-1-AP | WB:1:200 |
| Anti-TNP1 | Proteintech | 17178-1-AP | WB:1:500 |
| Anti-SIN3B | Santa Cruz | sc-13145 | WB:1:200 |
| Anti-EED | Proteintech | 16818-1-AP | WB:1:1000 |
| Anti-EZH2 | Proteintech | 21800-1-AP | WB:1:1000 |
| Anti-TDP43 | Proteintech | 12892-1-AP | WB:1:5000 |
| Anti-WDR5 | Proteintech | 15544-1-AP | WB:1:1000 |
| Anti-β-Catein | CST | 9561S | WB:1:1000 |
| Anti-HA | Sigma | H3663 | WB:1:5000 |
| Anti-Flag | Sigma | F7425 | WB:1:5000 |
| Anti-ubiquitin | Santa Cruz | sc-8017 | WB:1:1000 |
| Anti-Rabbit IgG | Proteintech | 98136-1-RR | IP:1:200 |
| Anti-GAPDH | Proteintech | 60004-1-Ig | WB:1:5000 |
| Anti-β-Actin | Abcam | ab8227 | WB:1:50000 |
| Anti-α-Tublin | Proteintech | 66031-1-Ig | WB:1:20000 |

Table S1 Antibodies used in this study (continued)

| Antibody | Vendor | Catalog No. | Working Dilution | |
| --- | --- | --- | --- | --- |
| Goat anti-Rabbit IgG (HRP) | Invitrogen | 31460 | WB:1:5000 |  |
| Goat anti-Mouse IgG (HRP) | Invitrogen | 31430 | WB:1:5000 |  |
| Alexa Fluor 488 donkey anti-rabbit immunoglobulin G | Invitrogen | A21206 | IF:1:400 |  |
| Alexa Fluor 555 donkey anti-rabbit immunoglobulin G | Invitrogen | A31572 | IF:1:400 |  |
| Alexa Fluor 488 donkey anti-mouse immunoglobulin G | Invitrogen | A21202 | IF:1:400 |  |
